# Alkaloids with Their Protective Effects Against A*β*_25-35_-Induced PC-12 Cell Injury from the Tubers of *Pinellia pedatisecta* Schott

**DOI:** 10.3390/molecules29215059

**Published:** 2024-10-26

**Authors:** Xu Chen, Yangang Cao, Kaili Ye, Yanling Liu, Fangge Chi, Ying Niu, Di Lu, Bingxian Zhao, Lan Chen, Xiaoke Zheng, Weisheng Feng

**Affiliations:** 1 School of Pharmacy, Henan University of Chinese Medicine, Zhengzhou 450046, China; 18638197038@163.com (X.C.); caoyangang1987@126.com (Y.C.); 18339998609@163.com (K.Y.); liuyl9696@163.com (Y.L.); chfangge@163.com (F.C.); 15516170034@163.com (Y.N.); 17837176971@163.com (D.L.); 15630160504@163.com (B.Z.); 18638931582@163.com (L.C.); zhengxk.2006@163.com (X.Z.); 2 The Engineering and Technology Center for Chinese Medicine Development of Henan Province China, Zhengzhou 450046, China; 3 Co-Construction Collaborative Innovation Center for Chinese Medicine and Respiratory Disease by Henan and Education Ministry of P.R. China, Zhengzhou 450046, China

**Keywords:** *Pinellia pedatisecta* Schott, Araceae, alkaloids, PC-12 cell injury

## Abstract

Seven new alkaloids [**1**, (±)-**2**, (±)-**3**, **4**, and **5**] and one new natural product (**6**), along with eight known analogues, were isolated from the tubers of *Pinellia pedatisecta* Schott. Their structures were determined by a comprehensive analysis of spectroscopic data, including HRESIMS, and electronic circular dichroism (ECD). In addition, the results of the bioactivity evaluation showed that compounds (±)-**3**, **6**, and **9** exhibited significantly protective effects against A*β*_25-35_-induced PC-12 cell injury and ameliorated cell viabilities by decreasing the levels of the reactive oxidative species (ROS) and mitochondrial membrane potential (MMP).

## 1. Introduction

*Pinellia pedatisecta* Schott (Araceae), known as a traditional Chinese herbal medicine, is mainly distributed in the center and north of China, which grows in undergrowth, valleys, riverbanks, or barren grasslands [1]. Previous phytochemical investigations on *P. pedatisecta* have led to the isolation of various types of compounds, including alkaloids, amino acids, terpenoids, and lectins [2,3,4,5]. Additionally, various pharmacological effects of this plant have been reported, including anti-tumour, anti-bacterial, and anti-inflammatory effects [6,7,8].

Alzheimer’s disease (AD) is irreversible, has an insidious onset and is the most common form of dementia in the elderly. The clinical symptoms of AD mainly include progressive cognitive dysfunction and memory loss. The deposition of the A*β* protein and neurofibrillary tangles were the most important pathological features in the brain of AD patients [9,10]. The number of patients with AD has been increasing in recent years; however, there remains a lack of relatively effective treatments for AD. Furthermore, a previous study demonstrated that alkaloids exhibited good therapeutic effects on AD [11,12], which piqued our interest to search for more alkaloids with a potential anti-AD effect from this plant. As a result, seven new alkaloids [**1**, (±)-**2**, (±)-**3**, **4**, and **5**] and one new natural product (**6**), along with eight known compounds, were isolated from the tubers of *P. pedatisecta*, and the protective effects of all compounds on PC-12 cell injury induced by A*β*_25-35_ were evaluated. This paper describes their isolation, structural elucidation and preliminary bioactivities.

## 2. Results and Discussion

### 2.1. Structure Characterization

**Figure 1:**
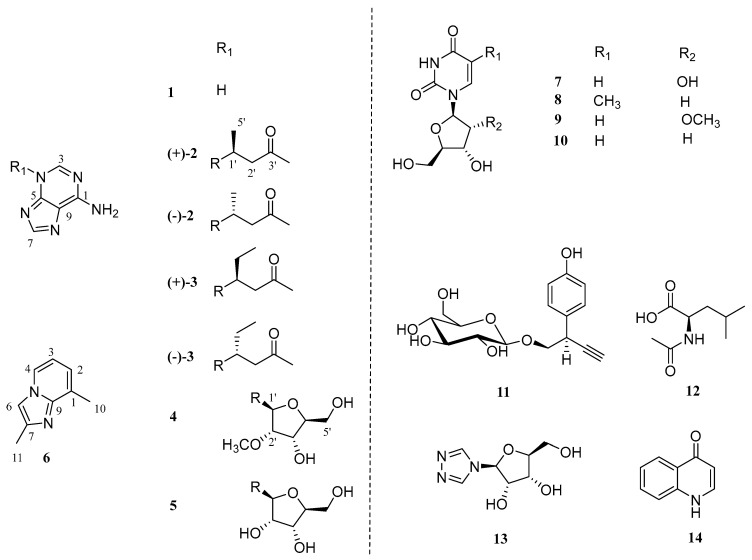
The chemical structures of compounds **1**–**14**.

The chemical investigations on the extract of the tubers of *P. pedatisecta* resulted in the characterization of compounds (**1**–**14**) (Figure 1).

**Figure 2:**
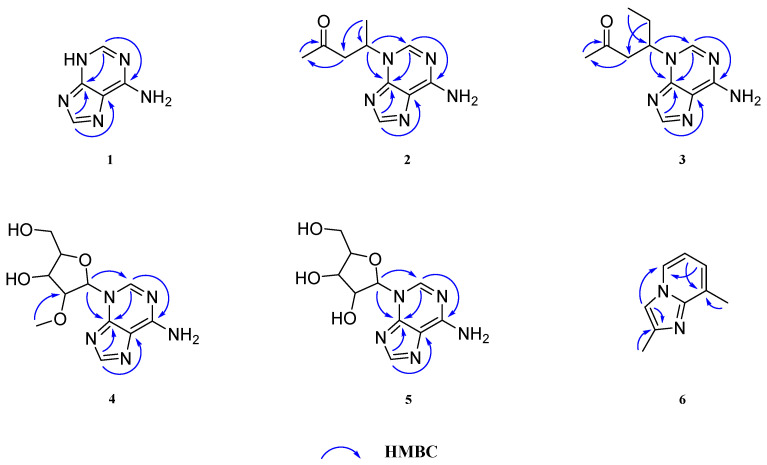
The key HMBC correlations of compounds **1**–**6**.

Compound **1**, a white amorphous powder, was given a molecular formula of C_5_H_5_N_5_ due to the HRESIMS ion at *m/z* 136.0615 [M + H]^+^ (calcd 136.0618). The 1D NMR data of **1** (Table 1) exhibited the signals of five sp^2^ quaternary carbons [*δ*_H_ 8.38 (1H, s, H-3), 8.33 (1H, s, H-7); *δ*_C_ 153.1 (C-1), 151.0 (C-5), 146.3 (C-3), 144.1 (C-7), 117.5 (C-9)]. A comparison of the above-mentioned NMR data of **1** with those of adenine implied that **1** had a similar structure with adenine [13]. The only significant difference between them was the chemical shifts of several carbons, especially the C-1, C-3 and C-7 [*δ*_C_ 153.1 (C-1), 146.3 (C-3), 144.1 (C-7) in **1**; *δ*_C_ 155.7 (C-1), 151.6 (C-3), 142.1 (C-7) in adenine], which indicated that the location of the -NH group in **1** may be different to that in adenine. The combination of the ^13^C NMR data with the HMBC correlations from H-3 to C-1/5 and from H-7 to C-5/9 (Figure 2) supported the idea that the -NH group was located at position 4. Therefore, the structure of **1** was assigned and named pedatisectine V.

**Figure 3:**
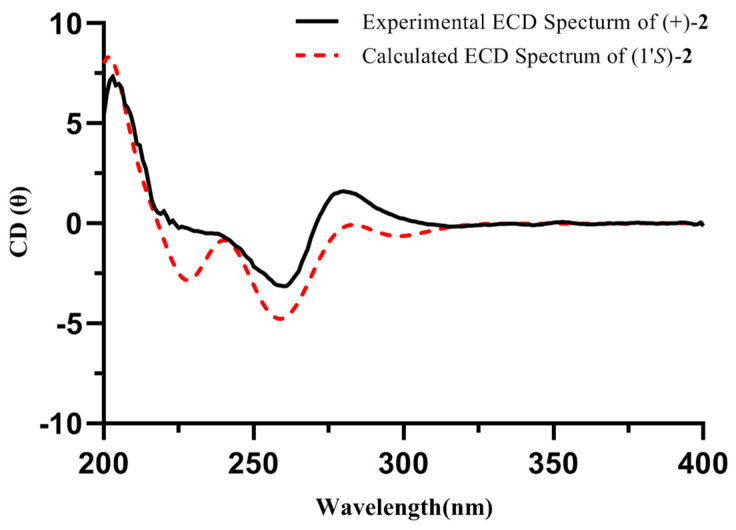
Calculated and experimental ECD spectra of (+)-**2** (both in CH_3_OH).

**Figure 4:**
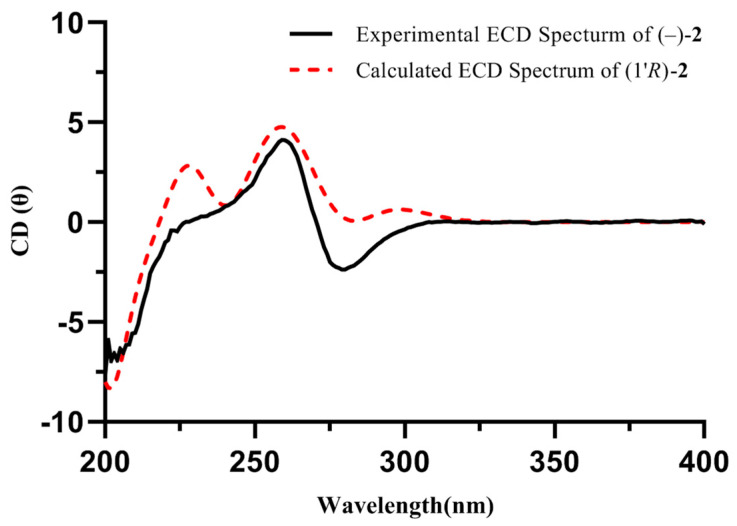
Calculated and experimental ECD spectra of (−)-**2** (both in CH_3_OH).

Compound **2** was a white amorphous powder with the molecular formula of C_10_H_13_N_5_O as deduced from the HRESIMS ion at *m/z* 220.1195 [M + H]^+^ (calcd for 220.1193). The ^1^H and ^13^C NMR data of **2**, as mentioned in Table 1, exhibited similarities to those of **1**. The major discrepancy was due to the presence of a 4-substituted-pentan-2-one unit in **2** [*δ*_H_ 5.14 (1H, m, H-1′) 3.45 (1H, dd, *J* = 18.2, 8.2 Hz, H-2′a), 2.68 (1H, dd, *J* = 18.2, 5.5 Hz, H-2′b), 2.11 (3H, s, H-4′), 1.62 (3H, d, *J* = 6.9 Hz, H-5′); *δ*_C_ 207.5 (C-3′), 49.9 (C-1′), 49.6 (C-2′), 30.0 (C-4′), 20.7 (C-5′)] [14]. Then, the HMBC correlations from H-1′ to C-3/5 indicated that the 4-substituted-pentan-2-one unit was located at the N-4 position. Compound **2** was optically inactive, suggesting that it was isolated as a racemate. Resolution via chiral-phase HPLC afforded the enantiomers (+)-**2** and (−)-**2**. To define the absolute configuration, ECD calculations of (*R*)-**2** and (*S*)-**2** were performed at the TDDFT-B3LYP/6-311+G(2d,p)//B3LYP/6-31G(d,p) in CH_3_OH and compared with the experimental ECD curves of (+)-**2** and (−)-**2** measured in CH_3_OH, whose results suggested that the absolute configurations of (+)-**2** and (–)-**2** were (*S*) and (*R*), respectively (Figure 3 and Figure 4). Thus, the structure of **2** was elucidated and named (±)-pedatisectine W.

Compound **3** was isolated as a white amorphous powder. The HRESIMS peak at *m/z* 234.1352 [M + H]^+^ (calcd for 234.1349) indicated that its molecular formula was C_11_H_15_N_5_O. The 1D NMR data of **3** (Table 2) were similar to those of **2**, except that a methyl group was replaced by an ethyl group at C-1′ in **3**, which was corroborated by the HMBC crosspeaks from H-6′ (*δ*_H_ 0.79) to C-4′/5′ (*δ*_C_ 30.0/28.4) and from H-5′ (*δ*_H_ 2.10/1.93) to C-2′(*δ*_C_ 47.6). Compound **3** was also optically inactive, and enantiomers (+)-**3** and (–)-**3** were obtained from the chiral separation. The absolute configurations of (+)-**3** and (–)-**3** were determined by comparing their optical rotation data with (+)-**2** and (–)-**2,** respectively. Therefore, the structure of **3** was identified and named (±)-pedatisectine X.

Compound **4** was isolated as a white amorphous powder. According to the HRESIMS at *m/z* 282.1196 [M + H]^+^, its molecular formula was inferred as C_11_H_15_N_5_O_4_. An analysis of the 1D NMR data of **4** (Table 2) showed that the most pronounced difference between **4** and **1** was the presence of an extra 2′-*O*-methylribose unit in **4** [*δ*_H_ 6.15 (1H, d, *J* = 5.1 Hz, H-1′), 4.48 (1H, t, *J* = 4.5 Hz, H-3′), 4.34 (1H, t, *J* = 5.0 Hz, H-2′), 4.14 (1H, m, H-4′) 3.89 (1H, d, *J* = 12.4, 2.7 Hz, H-5′a), 3.77 (1H, dd, *J* = 12.4, 3.0 Hz, H-5′b), 3.47 (3H, s, H-6′); *δ*_C_ 88.8 (C-1′), 87.9 (C-4′), 85.2 (C-2′), 70.5 (C-3′), 62.6 (C-5′), 58.8 (C-6′)] [15]. In addition, the correlations from H-1′ (*δ*_H_ 6.15) to C-5/3 (*δ*_C_ 149.8/148.5) supported the idea that the 2′-O-methylribose unit was connected via a C(1′)-N(4) bond in **4**. Thus, the structure of **4** was defined as shown and named pedatisectine Y.

Compound **5**, a white amorphous powder, showed an [M + H]^+^ peak at *m/z* 268.1043 in the HRESIMS data, corresponding to the molecular formula of C_10_H_13_N_5_O_4_, which was 14 Da (-CH_2_-) lower than that of **4**. The 1D NMR data (Table 2) of **5** were similar to those of **4**, except for the presence of a hydroxyl group instead of a methoxy group at C-2′ in **5**. This structural alteration resulted in a downshift of the signal for C-2′ by Δ*δ*_C_ 8.8 in comparison to that of **4**. Thus, the structure of **5** was identified and named pedatisectine Z.

Eight known compounds were identified as uridine (**7**) [16], thymidine (**8**) [17], 2′-*O*-methyluridine (**9**) [18], 2′-deoxyuridine (**10**) [19], (2*R*)-taxiphyllin (**11**) [20], *N*-acetyl-*L*-leucine (**12**) [21], 1,2,4-triazole-nucleosides (**13**) [22], and 2,3-dihydro-4(1*H*)-quinolone (**14**) [23] by comparing their experimental data with the literature data.

### 2.2. Biological Activity

A*β*_25–35_-induced AD models have been widely used. A*β*_25–35_ was reported to be the shortest fragment capable of forming large *β*-sheet fibrils and retaining the toxicity of full-length A*β*(_1–40/42_) peptides [24]. Meanwhile, attaining A*β*_25-35_ was one of the benchmarks for creating a rapid, acute and pathomimetic model of AD for the rapid screening of the therapeutic potential of new compounds in in vitro and in vivo studies [9]. Therefore, A*β*_25–35_ was chosen to induce the AD model in this experiment.

**Figure 5:**
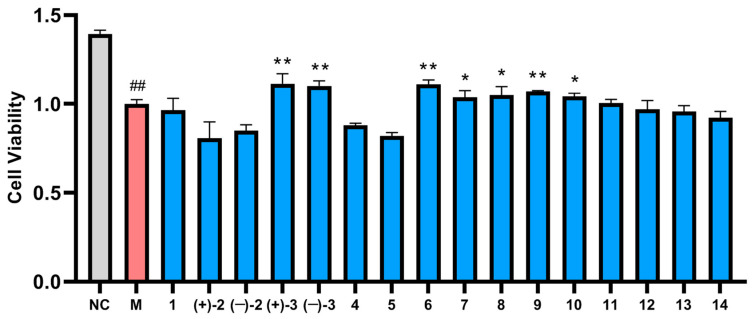
The effects of compounds **1**–**14** on A*β*_25-35_-induced PC-12 cell damage (*x ± sd*, *n* = 4) (* *p* < 0.05 and ** *p* < 0.01 compared with M group. ^##^ *p* < 0.01 compared with NC group).

All of the isolated compounds were tested for their protective effects against A*β*_25-35_-induced PC-12 cell injury in vitro. As shown in Figure 5, compared with the normal (NC) group, the cell viability of the model (M) group was significantly decreased (*p* < 0.01). Compared with M group, the cell viabilities of compounds (±)-**3**, **6**, and **9** were significantly increased (*p* < 0.01), which indicated that these compounds could significantly protect PC-12 cells against A*β*_25-35_-induced injury at 10 *μ*M. Then, compounds (±)-**3**, **6**, and **9** were selected for further research based on the above results and the weight of the separated compounds.

**Figure 6:**
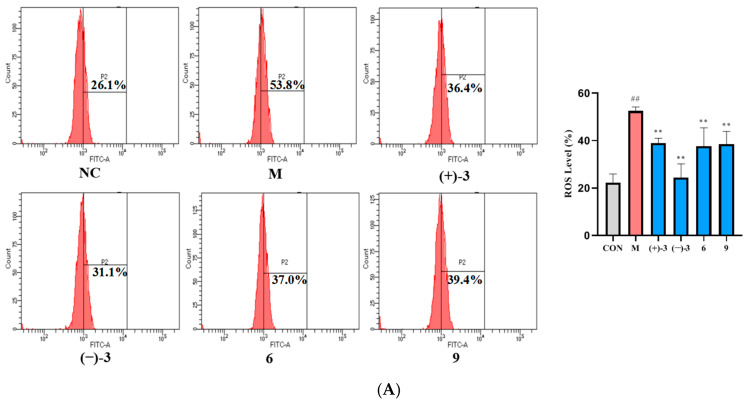

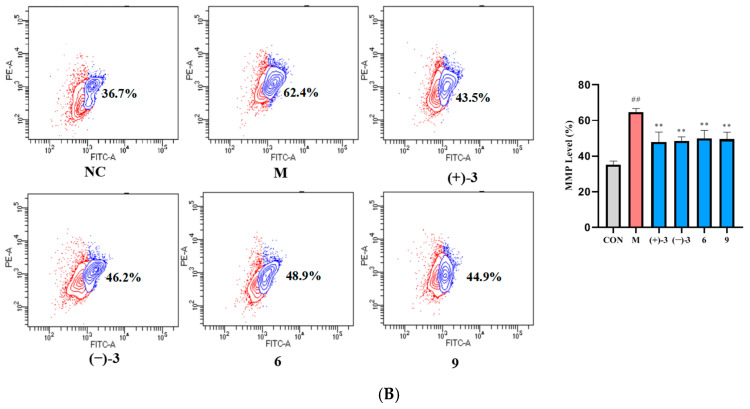
(**A**): Effects of compounds (±)**3**, **6**, and **9** on the level of the ROS in A*β*_25-35_-induced PC-12 cell. (**B**): Effects of compounds (±)**3**, **6**, and **9** on the level of the MMP in A*β*_25-35_-induced PC-12 cell. (*x ± sd*, *n* = 4) (** *p* < 0.01 compared with M group. ^##^ *p* < 0.01 compared with NC group).

During the development of AD, it can cause oxidative stress and mitochondrial damage, which may cause the changes in the levels of the reactive oxidative species (ROS) and the mitochondrial membrane potential (MMP). Pharmacological studies have shown that alkaloids possessed an outstanding therapeutic effect on AD [25,26], so the above indicators were been detected to discover potential therapeutic compounds. As shown in Figure 6, compared with the NC group, the levels of ROS and MMP in the M group were significantly increased (*p* < 0.01). Compared with M group, compounds (±)-**3**, **6**, and **9** could significantly reduce ROS and MMP levels (*p* < 0.01), which indicated that these compounds may reduce A*β*_25-35_-induced PC-12 cell injury by decreasing the levels of ROS and MMP.

The pharmacological effects of *P. pedatisecta* were mainly focused on anti-tumour, especially cervical cancer and ovarian cancer, with less research and attention paid to other pharmacological effects in the previous literature. In this study, the protective effects of compounds isolated from *P. pedatisecta* on A*β*_25-35_-induced cell injury of PC-12 were investigated for the first time. The preliminary results of this experiment have an important potential value in expanding the research on *P. pedatisecta*.

## 3. Experimental Section

### 3.1. General Experimental Procedures

In this study, 1D and 2D NMR spectra were carried out on a Bruker Avance III 500 spectrometer (Bruker, Germany). MS spectra were detected by a Bruker maXis HD mass spectrometer (Bruker, Germany). UV spectra were acquired using a ThermoEVO 300 spectrometer (Thermo, Waltham, MA, USA). IR spectra were recorded on a Thermo Nicolet IS 10 spectrometer (Thermo, Waltham, MA, USA). Optical rotations were recorded on a Rudolph AP-Ⅳ polarimeter (Rudolph, Hackettstown, NJ, USA). ECD spectra were obtained on an Applied Photophysics Chirascan qCD spectropolarimeter (AppliedPhotophysics, Leatherhead, Surrey, UK). Semi-preparative HPLC were performed on a Saipuruisi LC 52 HPLC system with a UV/vis 50 detector (Saipuruisi, Beijing, China) and a YMC Pack ODS A column (20 × 250 mm, 5 μm; YMC, Kyoto, Japan). Chiral-phase separation was conducted on an HPLC system, using a CHIRALAL IC column (10 × 250 mm) (Daicel Chiral Technologies Co., Ltd., China). Column chromatography was conducted using silica gel (100–300 mesh, Marine Chemical Industry Qingdao, China), MCI gel CHP-20, ODS gel (50 μm), Toyopearl HW-40C, and Sephadex LH-20 (40–70 μm, TOSOH Corp., Tokyo, Japan). The chemical reagents were supplied by Fuyu Fine Chemical Industry, Tianjin, China.

### 3.2. Plant Material

The tubers of *P. pedatisecta* were collected in Yuzhou, Henan province, China, in November 2022 and identified by Professor Chengming Dong of the Henan University of Chinese Medicine. A voucher specimen (No.20221101) is deposited at the Department of Natural Medicinal Chemistry, the Henan University of Chinese Medicine, Zhengzhou, China.

### 3.3. Extraction and Isolation

The tubers (45.0 kg) were extracted by the tissue crushing method with 70% aqueous acetone at room temperature to obtain a crude extract (2.8 kg). The extract was dispersed in H_2_O and sequentially partitioned with EtOAc and *n*-BuOH, respectively. The EtOAc fraction (73.0 g) was separated by silica gel column chromatography (CC) eluted with petroleum ether-EtOAc (100:0−0:100) and EtOAc-CH_3_OH (100:0−0:100) gradient systems to yield ten subfractions (E1−E10).

Subfraction E2 (9.5 g) was subjected to ODS CC eluted with a CH_3_OH-H_2_O (10:90−100:0) gradient system to obtain eight subfractions (E2-1−E2-8). Subfraction E2-4 (1.0 g) was rechromatographed with silica gel CC by a CH_2_Cl_2_-MeOH (20:1−0:1) gradient system and semi-preparative HPLC (CH_3_OH-H_2_O, 21:79) to obtain compounds **2** (4.2 mg, *t_R_* = 25.2 min) and **3** (4.0 mg, *t_R_* = 30.1 min). Moreover, **2** was separated by chiral HPLC using a CHIRALAL IC column eluted with isopropanol-n-hexane (20:80) to produce compounds (+)-**2** (1.5 mg, t*_R_* = 32.5 min) and (−)-**2** (1.4 mg, t*_R_* = 35.0 min). Additionally, **3** was separated by chiral HPLC using a CHIRALAL IC column eluted with isopropanol-n-hexane (40:60) to obtain compounds (+)-**3** (1.5 mg, *t_R_* = 13.2 min) and (−)-**3** (1.5 mg, *t_R_* = 15.2 min). Compounds **1** (3.4 mg, *t_R_* = 21.2 min) and **4** (6.4 mg, *t_R_* = 30.0 min) were isolated from E2-5 (820.1 mg) using silica gel CC eluted with a CH_2_Cl_2_-MeOH (25:1−0:1) gradient system and semi-preparative HPLC (MeOH-H_2_O, 20:80). Subfraction E2-6 (1.6 g) was separated by silica gel CC eluted with a CH_2_Cl_2_-MeOH (15:1−0:1) gradient system and then further purified by Toyopearl HW-40C CC (CH_3_OH-H_2_O 80:20) to obtain seven subfractions (E2-6-1-1−E2-6-1-7). Compounds **7** (3.6 mg, *t_R_* = 18.5 min) and **9** (4.8 mg, *t_R_* = 22.7 min) were obtained from E2-6-1-1 (90.7 mg) using semi-preparative HPLC (CH_3_CN-H_2_O, 20:80). Fraction E2-6-1-3 (335.2 mg) was purified by Sephadex LH-20 CC (CH_3_OH-H_2_O 30:70) and semi-preparative HPLC (CH_3_OH-H_2_O, 24:76) to produce compounds **8** (8.0 mg, *t_R_* = 18.2 min) and **10** (6.3 mg, *t_R_* = 21.2 min). The further separation of subfraction E2-6-1-6 (90.8 mg) using semi-preparative HPLC (CH_3_OH-H_2_O, 10:90) yielded compound **5** (15.9 mg, *t_R_* = 15.2 min).

Subfraction E3 (5.4 g) was chromatographed with MCI gel CHP-20 CC by a MeOH-H_2_O (10:90−100:0) gradient system to yield five subfractions (E3-1−E3-5). Subfraction E3-3 (2.0 g) was separated by Sephadex LH-20 CC (MeOH-H_2_O 30:90−100:0) to provide six subfractions (E3-3-1−E3-3-6). Subfraction E3-3-2 (652.3 mg) was purified by silica gel CC eluted with a CH_2_Cl_2_-MeOH (50:1−1:1) gradient system to yield in four subfractions (E3-3-2-1−E3-3-2-4). Subfraction E3-3-2-2 (90.2 mg) was purified by semi-preparative HPLC (CH_3_OH-H_2_O, 25:75) to obtain compounds **6** (7.5 mg, *t_R_* = 15.2 min) and **12** (5.5 mg, *t_R_* = 20.2 min). Then, subfraction E3-3-2-3 (131.6 mg) was purified by semi-preparative HPLC (CH_3_OH-H_2_O 28:72) to produce compounds **11** (6.0 mg, *t_R_* = 19.0 min), **13** (6.1 mg, *t_R_* = 25.5 min), and **14** (5.3 mg, *t_R_* = 32.2 min).

Pedatisectine V (**1**): white amorphous powder; UV (CH_3_OH) *λ*_max_: 206, 260; IR (iTR) *ν*_max_ 3388, 2106, 1661 cm^−1^; HRESIMS *m/z* 136.0615 [M + H]^+^ (calcd for C_5_H_6_N_5_, 136.0618); ^1^H and ^13^C NMR data (see Table 1).

Pedatisectine W (**2**): White amorphous powder; UV (CH_3_OH) *λ*_max_: 212, 260 nm; IR (iTR) *ν*_max_ 3383, 2130, 1644 cm^−1^; HRESIMS *m/z* 220.1195 [M + H]^+^ (calcd for C_10_H_14_N_5_O, 220.1193); ^1^H and ^13^C NMR data, (see Table 1). (+)-**2**: [α${]}_{D}^{25}$ +15.1 (c 0.03, CH_3_OH), ECD (CH_3_OH) *λ*_max_ (Δ*ε*) 202 (+8.1), 260 (−2.1), 280 (+2.6); (−)-**2**: [α${]}_{D}^{25}$ -16.7 (c 0.03, CH_3_OH), ECD (CH_3_OH) *λ*_max_ (Δ*ε*) 202 (−7.9), 260 (+3.1), 280 (−3.4).

Pedatisectine X (**3**): White amorphous powder; UV (CH_3_OH) *λ*_max_: 208, 261 nm; IR (iTR) *ν*_max_ 3427, 1682, 1205, 1141 cm^−1^; HRESIMS *m/z* 234.1352 [M + H]^+^ (calcd for C_11_H_16_N_5_O, 234.1349); ^1^H and ^13^C NMR data (see Table 2). (+)-**3**: [α${]}_{D}^{25}$ +16.2 (c 0.03, CH_3_OH), ECD (CH_3_OH) *λ*_max_ (Δ*ε*) 205 (+3.8), 258(−1.8), 278 (+2.1); (−)-**3**: [α${]}_{D}^{25}$ -17.5 (c 0.03, CH_3_OH), ECD (CH_3_OH) *λ*_max_ (Δ*ε*) 205 (−5.5), 258 (+2.0), 278 (−2.6).

Pedatisectine Y (**4**): white amorphous powder; [α${]}_{D}^{25}$ +10.1 (c 0.13, CH_3_OH); UV (CH_3_OH) *λ*_max_: 203, 260 nm; IR (iTR) *ν*_max_ 3346, 1651 cm^−1^; HRESIMS *m/z* 282.1196 [M + H]^+^ (calcd for C_11_H_16_N_5_O_4_, 282.1197); ^1^H and ^13^C NMR data (see Table 2).

Pedatisectine Z (**5**): white amorphous powder; [α${]}_{D}^{25}$ +16.1 (c 0.32, CH_3_OH); UV (CH_3_OH) *λ*_max_: 207, 259 nm; IR (iTR) *ν*_max_ 3376, 2130, 1644 cm^−1^; HRESIMS *m/z* 268.1043 [M + H]^+^ (calcd for C_10_H_14_N_5_O_4_, 268.1040); ^1^H and ^13^C NMR data (see Table 3).

2,8-dimethyl-imidazo[1,2-α]pyridine (**6**): white amorphous powder; UV (CH_3_OH) *λ*_max_: 223, 238, 297, 345 nm; IR (iTR) *ν*_max_ 3412, 1678, 1202, 1139 cm^−1^; HRESIMS *m/z* 147.0921 [M + H]^+^ (calcd for C_9_H_11_N_2_, 147.0917); ^1^H and ^13^C NMR data (see Table 3).

### 3.4. Computational Analysis

The conformations of **2** was conducted by GMMX using the MMFF94 force field with the energy window of 3.5 kcal/mol, which was part of the function of GuassView 6.0 software. The conformers were optimized with density functional theory (DFT) at the B3LYP/6-31G (d, p) level. After checking the imaginary frequencies (Tables S5 and S6), the ECD calculations of conformers with Boltzmann distributions over 1% were further calculated by the TDDFT method at the B3LYP/6-311G+(2d,p) level in CH_3_OH by GuassView 6.0 software [27]. SpecDis_1170 software was used to extract the ECD curves.

### 3.5. MTT Assay

Briefly, the PC-12 cells (CL-0418, Wuhan Punosai Life Technology Co., Ltd.) were seeded in 96-well plates at 5 × 10^4^ cells/well in a 5% CO_2_ incubator at 37 °C. After 24 h, the cells were divided into the normal group (NC), model group (M, A*β*_25-35_ 0.1 μM), and treatment groups (each using compound 10 μM + A*β*_25-35_ 0.1 μM). Then, the MTT assay was performed as previously described [9]. The OD values of three groups were measured at 490 nm using a microplate reader (Thermo Scientific, Boston, MA, USA). Experiments are performed in triplicate and the results are shown in Figure 4.

### 3.6. Flow Cytometry

The PC-12 cells were seeded into 6-well plates at a density of 8 × 10^4^ cells/mL and divided into the normal group (NC), model group (M, A*β*_25-35_ + 0.1 μM), treatment groups (10 μM, A*β*_25-35_ + 0.1 μM). After 24 h of drug treatment, the cellular levels of intracellular ROS and MMP were detected using an ROS detection kit (G1706, Wuhan Saiweier Biotechnology Co., Ltd, Wuhan, China) and an MMP detection kit (M8650, Beijing Solebao Technology Co., Ltd, Beijing, China).

### 3.7. Statistical Analyses

All biological data were analyzed by one-way analysis of variance, followed by the least significant difference test using SPSS software version 20.0. The results were expressed as the mean ± standard deviation (SD) and *p* < 0.05 was considered to indicate significance.

## 4. Conclusions

Seven new alkaloids [**1**, (±)-**2**, (±)-**3**, **4**, and **5**] and one undescribed natural product (**6**), together with eight known analogues, were isolated from the tubers of *P. pedatisecta*, which enriched the chemical content of this plant. The biological activity results showed that compounds (±)**3**, **6**, and **9** exhibited obviously protective effects on PC-12 cells against A*β*_25-35_-induced injury. The above research results illustrate the important potential value of future development and research on *P. pedatisecta* and provide a theoretical basis for elucidating its potential application in AD. Subsequently, we will discover more bioactive alkaloids from *P. pedatisecta* to carry out further research on the treatment of AD.

## Figures and Tables

**Table 1:** ^1^H (500 MHz) and ^13^C (125 MHz) NMR spectroscopic and HMBC data of compounds **1**–**2** in CD_3_OD.

Position	1			2		
	*δ*_H_ (*J* in Hz)	*δ*_C_ (Type)	HMBC(H→C)	*δ*_H_ (*J* in Hz)	*δ*_C_ (Type)	HMBC(H→C)
1		153.1 (C)			153.5 (C)	
3	8.38 (s)	146.3 (CH)	1, 5	8.33 (s)	147.3 (CH)	1, 5
5		151.0 (C)			150.5 (C)	
7	8.33 (s)	144.1 (CH)	5, 9	8.31 (s)	143.9 (CH)	5, 9
9		117.5 (C)			120.2 (C)	
1′				5.14 (m)	49.9 (CH)	3, 5, 2′, 3′, 5′;
2′				3.45 (dd, 18.2, 8.2) 2.68 (dd, 18.2, 5.5)	49.6 (CH_2_)	1′, 3′, 4′, 5′
3′					207.5 (C)	
4′				2.11 (s)	30.0 (CH_3_)	2′, 3′
5′				1.62 (d, 6.9)	20.7 (CH_3_)	1′, 2′

**Table 2:** ^1^H (500 MHz) and ^13^C (125 MHz) NMR spectroscopic and HMBC data of compounds **3**–**4** in CD_3_OD.

Position	3			4		
	*δ*_H_ (*J* in Hz)	*δ*_C_ (Type)	HMBC(H→C)	*δ*_H_ (*J* in Hz)	*δ*_C_ (Type)	HMBC(H→C)
1		154.0 (C)			154.1 (C)	
3	8.26 (s)	149.8 (CH)	1, 5	8.56 (s)	148.5 (CH)	1, 5
5		150.5 (C)			149.8 (C)	
7	8.26 (s)	143.7 (CH)	5, 9	8.33 (s)	143.1 (CH)	5, 9
9		120.2 (C)			120.7 (C)	
1′	4.86 (m)	55.6 (CH)	3, 5, 2′, 3′, 5′	6.15 (d, 5.1)	88.8 (CH)	3, 5, 2′, 3′, 5′
2′	3.47 (dd, 18.2, 8.9) 3.19 (dd, 18.2, 4.8)	47.6 (CH_2_)	1′, 3′, 4′, 5′	4.34 (t, 5.0)	85.2 (CH)	1′, 3′, 4′, 6′
3′		207.7 (C)		4.48 (t, 4.5)	70.5 (CH)	1′, 2′, 4′, 5′
4′	2.09 (s)	30.0 (CH_3_)	2′, 3′	4.14 (m)	87.9 (CH)	1′, 2′, 3′, 5′
5′	2.10 (m) 1.93 (m)	28.4 (CH_2_)	1′, 2′, 6′	3.89 (dd, 12.4, 2.7) 3.77 (dd, 12.4, 3.0)	62.6 (CH_2_)	3′, 4′
6′	0.79 (t, 7.4)	10.7 (CH_3_)	1′, 5′	3.47 (s)	58.8 (CH_3_)	2′

**Table 3:** ^1^H (500 MHz) and ^13^C (125 MHz) NMR spectroscopic and HMBC data of compounds **5**–**6** in CD_3_OD.

Position	5			6		
	*δ*_H_ (*J* in Hz)	*δ*_C_ (Type)	HMBC(H→C)	*δ*_H_ (*J* in Hz)	*δ*_C_ (Type)	HMBC(H→C)
1		152.8 (C)			127.2 (C)	
2				7.88 (d, 4.4)	116.2 (CH)	4, 9, 10
3	8.60 (s)	146.6 (CH)	1, 5	7.39 (dd, 4.4, 2.6)	121.0 (CH)	1, 2, 4
4				8.14 (d, 1.7)	122.8 (CH)	2, 6, 9
5		149.8 (C)				
6				7.44 (s)	114.1 (CH)	4, 9, 11
7	8.33 (s)	143.7 (CH)	5, 9		130.7 (C)	
9		120.5 (C)			149.5 (C)	
10				2.93 (s)	16.9 (CH_3_)	1, 2, 9
11				2.68 (s)	15.5 (CH_3_)	6, 7
1′	6.09 (d, 5.4)	90.6 (CH)	3, 5, 2′, 3′, 5′			
2′	4.34 (t, 5.0)	76.4 (CH)	1′, 3′, 4′, 6′			
3′	4.63 (t, 5.2)	72.0 (CH)	1′, 2′, 4′, 5′			
4′	4.15 (m)	87.6 (CH)	1′, 2′, 3′, 5′			
5′	3.88 (dd, 12.3, 3.0) 3.78 (dd, 12.3, 3.3)	62.7 (CH_2_)	3′, 4′			

## Data Availability

The data presented in this study are available in the Main Text of this paper and the Supplementary Materials.

## Supplementary Materials

The following supporting information can be downloaded at: https://www.mdpi.com/article/10.3390/molecules29215059/s1, Figure S1–S40: 1D/2D NMR, HR-ESIMS, IR and UV spectra of compounds **1**–**6**. Tables S1–S6: Coordinates of the conformers of (*R*)-**2** and (*S*)-**2** used after optimization and frequency, EE + thermal free energy correction and Boltzmann distribution of the conformers of (*R*)-**2** and (*S*)-**2** used after optimization and frequency, Related frequencies of the conformers of (*R*)-**2** and (*S*)-**2** after optimization and frequency.
