# Supplementary material for: Alkaloids with Their Protective Effects Against Aβ25-35-Induced PC-12 Cell Injury from the Tubers of Pinellia pedatisecta Schott
Source: Molecules. 2024 Oct 26;29(21):5059. doi: 10.3390/molecules29215059 (PMC11547655; doi:10.3390/molecules29215059)
Supplement: Supplementary file 1 [file molecules-29-05059-s001.zip › molecules-3266948-supplementary.pdf]

Alkaloids with their protective effects against Aβ_{25-35}-induced PC-12 cell injury from the tubers of *Pinellia pedatisecta* Schott

Xu Chen[1,2], Yan-gang Cao[1,2], Kai-li Ye[1,2], Yan-ling Liu[1,2], Fang-ge Chi[1,2], Ying Niu[1,2], Di Lu[1,2], Bing-xian Zhao[1,2], Lan Chen[1,2], Xiao-ke Zheng[1,2,3],Wei-sheng Feng[1,2,3*]

[1]*School of Pharmacy, Henan University of Chinese Medicine, Zhengzhou 450046, China*

[2]*The Engineering and Technology Center for Chinese Medicine Development of Henan Province China,Zhengzhou 450046, China*

[3]*Co-construction Collaborative Innovation Center for Chinese Medicine and Respiratory Diseases by Henan & Education Ministry of P.R. China, Zhengzhou 450046, China*

*Corresponding author:

School of Pharmacy, Henan University of Chinese Medicine, Zhengzhou 450046, P.R. China.

Tel: +86 371 60190296

*Corresponding author

E-mail addresses: zhengxk.2006@163.com(XK Zheng), fwsh@hactcm.edu.cn(WS Feng)

Contents

Figure S1. ^{1}H NMR spectrum (500MHz, CD_3OD) of **1**

Figure S2. ^{13}C NMR spectrum (125MHz, CD_3OD) of **1**

Figure S3. HSQC spectrum of **1**

Figure S4. HMBC spectrum of **1**

Figure S5. HR-ESI-MS spectrum of **1**

Figure S6. UV spectrum of **1**

Figure S7. IR spectrum of **1**

Figure S8. ^{1}H NMR spectrum (500MHz, CD_3OD) of **2**

Figure S9. ^{13}C NMR spectrum (125MHz, CD_3OD) of **2**

Figure S10. HSQC spectrum of **2**

Figure S11. HMBC spectrum of **2**

Figure S12. HR-ESI-MS spectrum of **2**

Figure S13. UV spectrum of **2**

Figure S14. IR spectrum of **2**

Figure S15. ^{1}H NMR spectrum (500MHz, CD_3OD) of **3**

Figure S16. ^{13}C NMR spectrum (125MHz, CD_3OD) of **3**

Figure S17. HSQC spectrum of **3**

Figure S18. HMBC spectrum of **3**

Figure S19. HR-ESI-MS spectrum of **3**

Figure S20. UV spectrum of **3**

Figure S21. IR spectrum of **3**

Figure S22. ^{1}H NMR spectrum (500MHz, CD_3OD) of **4**

Figure S23. ^{13}C NMR spectrum (125MHz, CD_3OD) of **4**

Figure S24. HSQC spectrum of **4**

Figure S25. HMBC spectrum of **4**

Figure S26. HR-ESI-MS spectrum of **4**

Figure S27. UV spectrum of **4**

Figure S28. IR spectrum of **4**

Figure S29. ^{1}H NMR spectrum (500MHz, CD_3OD) of **5**

Figure S30. ^{13}C NMR spectrum (125MHz, CD_3OD) of **5**

Figure S31. HR-ESI-MS spectrum of **5**

Figure S32. UV spectrum of **5**

Figure S33. IR spectrum of **5**

Figure S34. ^{1}H NMR spectrum (500MHz, CD_3OD) of **6**

Figure S35. ^{13}C NMR spectrum (125MHz, CD_3OD) of **6**

Figure S36. HSQC spectrum of **6**

Figure S37. HMBC spectrum of **6**

Figure S38. HR-ESI-MS spectrum of **6**

Figure S39. UV spectrum of **6**

Figure S40. IR spectrum of **6**

Figure S41. ^{1}H NMR spectrum (500MHz, CD_3OD) of **7**

Figure S42. ^{13}C NMR spectrum (125MHz, CD_3OD) of **7**

Figure S43. ^{1}H NMR spectrum (500MHz, CD_3OD) of **8**

Figure S44. ^{13}C NMR spectrum (125MHz, CD_3OD) of **8**

Figure S45. ^{1}H NMR spectrum (500MHz, CD_3OD) of **9**

Figure S46. ^{13}C NMR spectrum (125MHz, CD_3OD) of **9**

Figure S47. ^{1}H NMR spectrum (500MHz, CD_3OD) of **10**

Figure S48. ^{13}C NMR spectrum (125MHz, CD_3OD) of **10**

Figure S49. ^{1}H NMR spectrum (500MHz, CD_3OD) of **11**

Figure S50. ^{13}C NMR spectrum (125MHz, CD_3OD) of **11**

Figure S51. ^{1}H NMR spectrum (500MHz, CD_3OD) of **12**

Figure S52. ^{13}C NMR spectrum (125MHz, CD_3OD) of **12**

Figure S53. ^{1}H NMR spectrum (500MHz, CD_3OD) of **13**

Figure S54. ^{13}C NMR spectrum (125MHz, CD_3OD) of **13**

Figure S55. ^{1}H NMR spectrum (500MHz, CD_3OD) of **14**

Figure S56. ^{13}C NMR spectrum (125MHz, CD_3OD) of **14**

Table S1 Coordinates of the conformers of (*R*)-**2** used after optimization and frequency.

Table S2 Coordinates of the conformers of (*S*)-**2** used after optimization and frequency.

Table S3. The electronic energy (EE) + thermal free energy correction and Boltzmann

distribution of the conformers of (*R*)-**2** used after optimization and frequency.

Table S4. The electronic energy (EE) + thermal free energy correction and Boltzmann distribution of the conformers of (*S*)-**2** used after optimization and frequency.

Table S5. Related frequencies of the conformers of (*R*)-**2** after optimization and frequency.

Table S6. Related frequencies of the conformers of (*S*)-**2** after optimization and frequency.

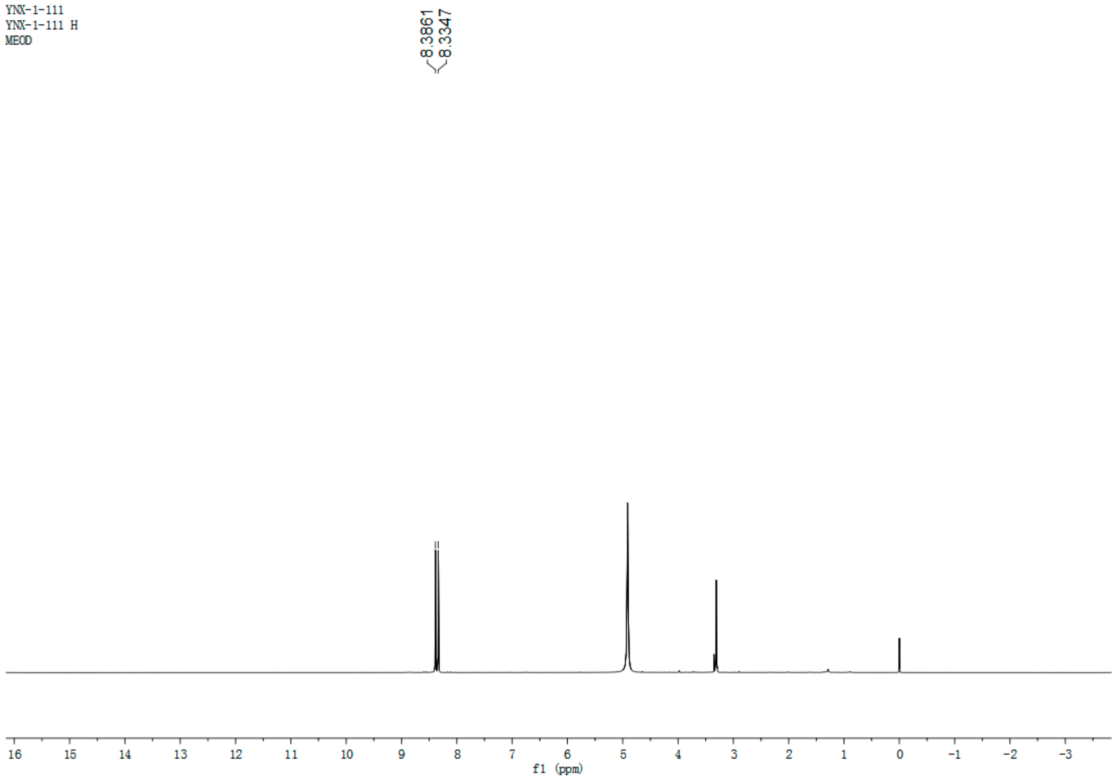

Figure S1. ^{1}H NMR spectrum (500MHz, CD_3OD) of **1**

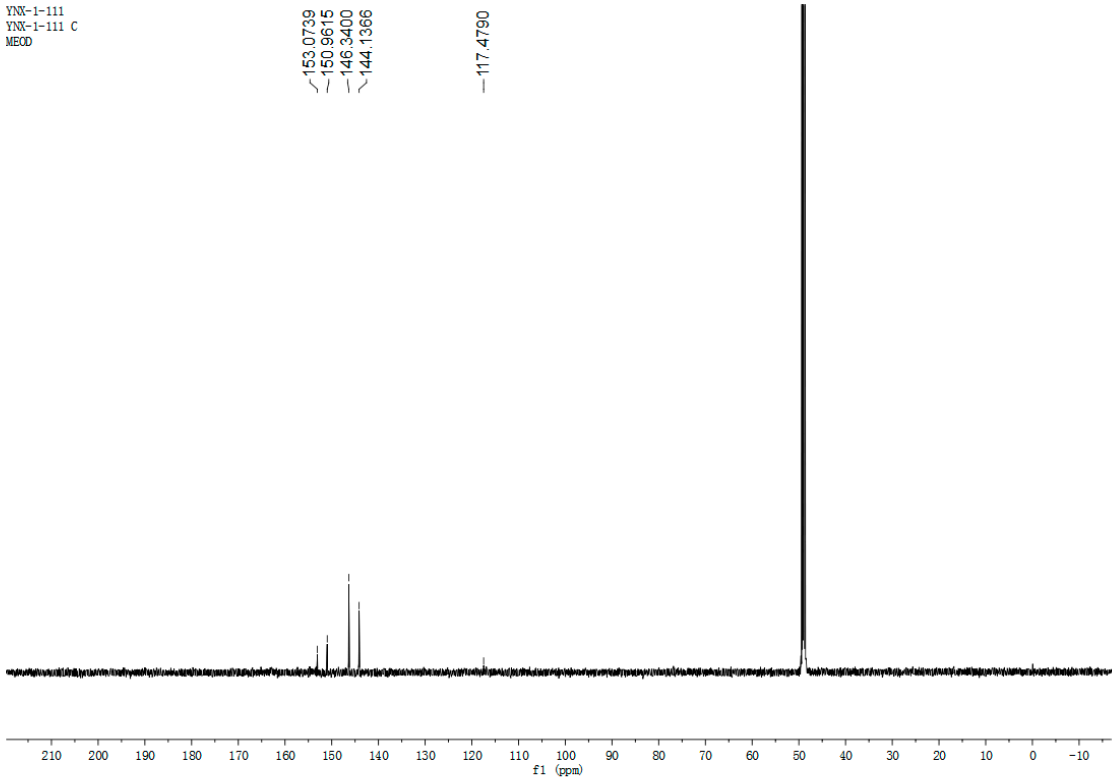

Figure S2. ^{13}C NMR spectrum (125MHz, CD_3OD) of **1**

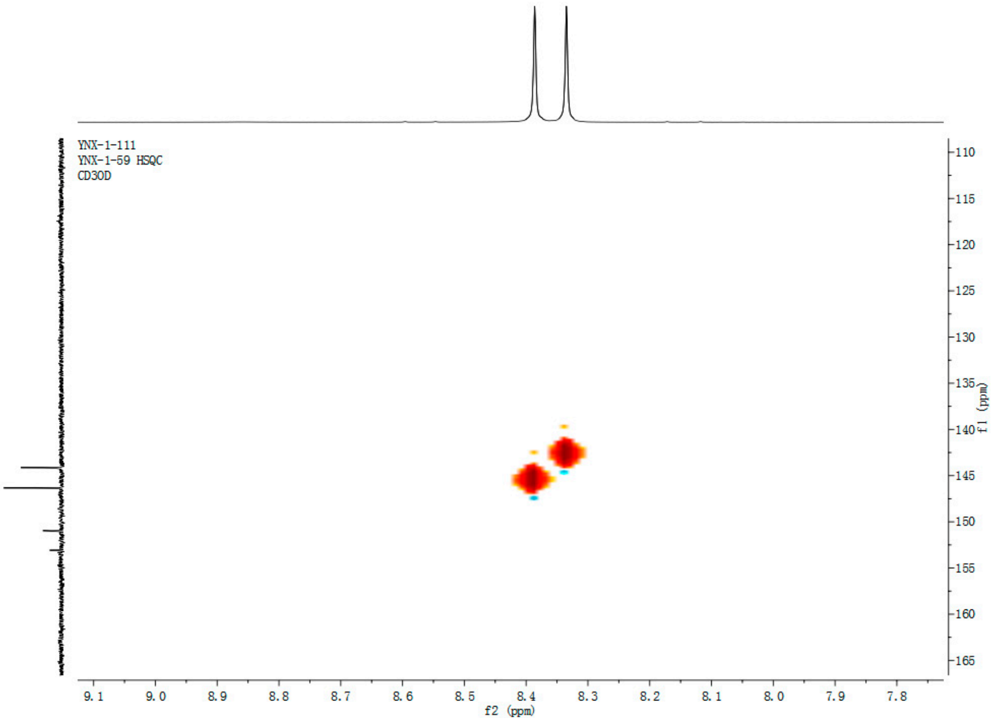

Figure S3. HSQC spectrum of **1**

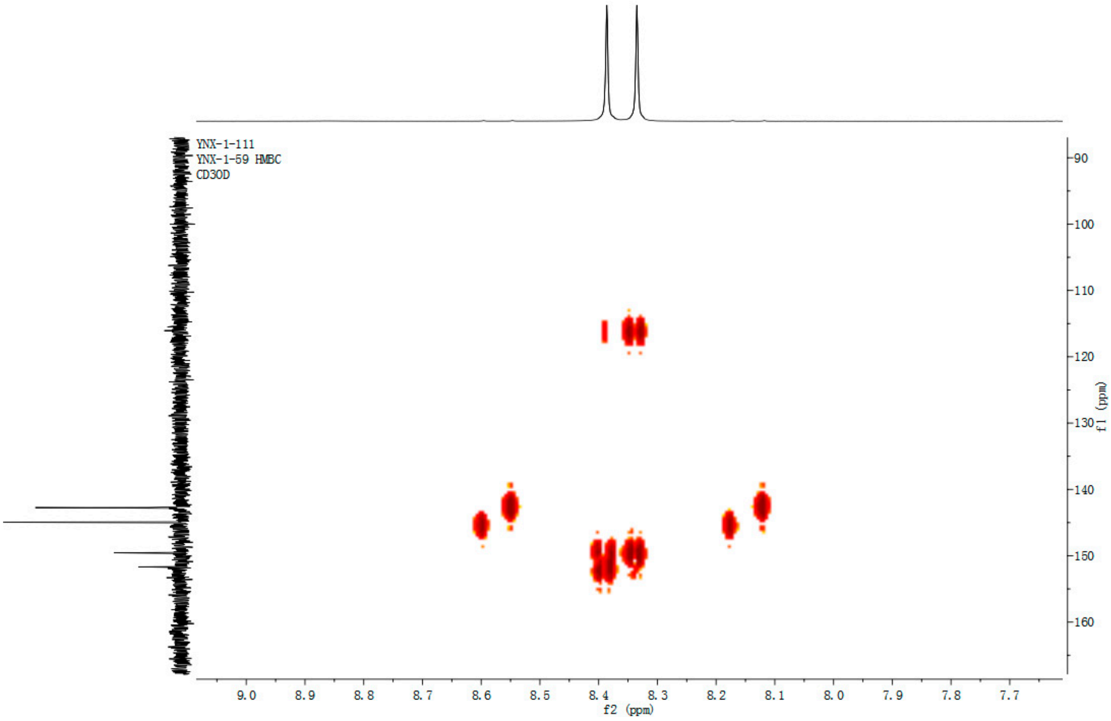

Figure S4. HMBC spectrum of **1**

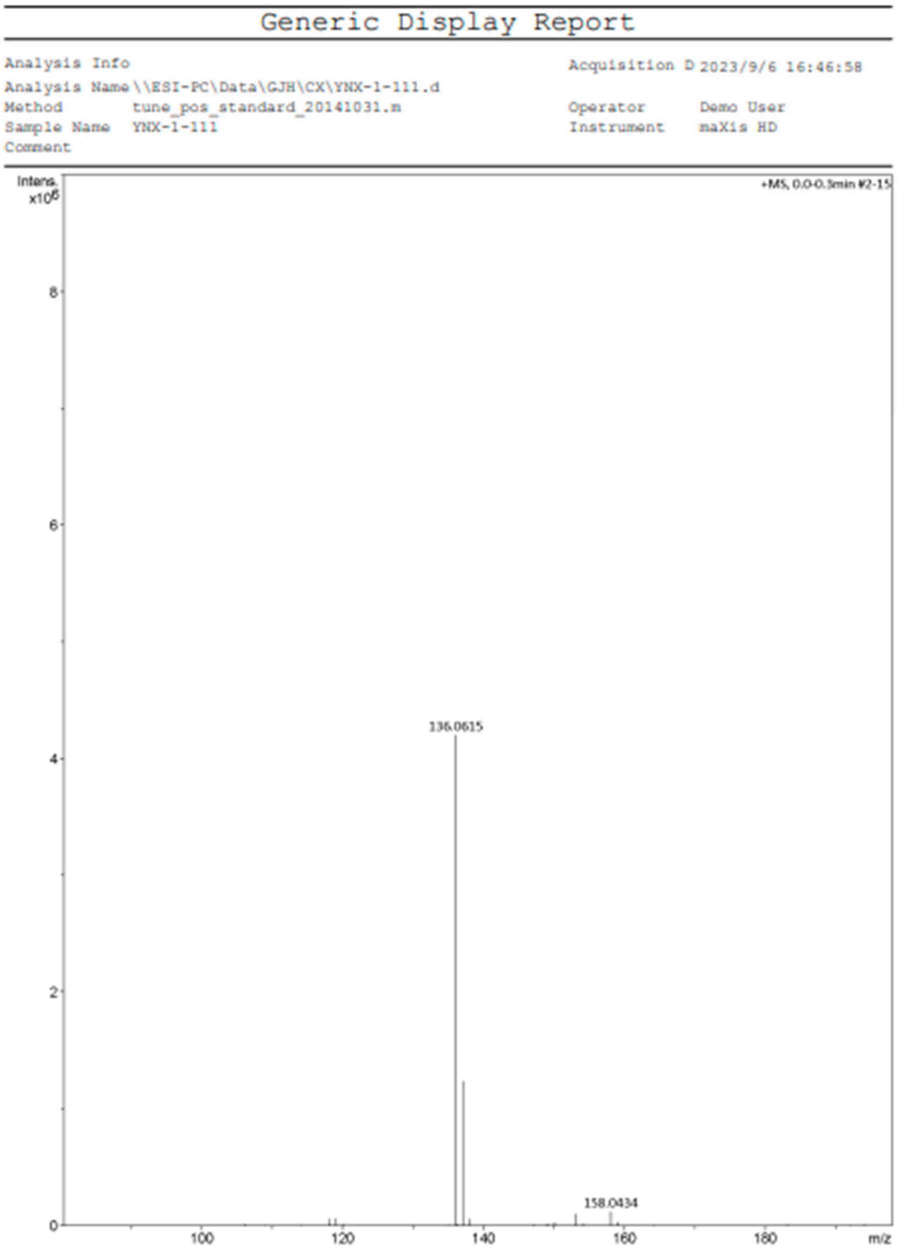

Figure S5. HR-ESI-MS spectrum of **1**

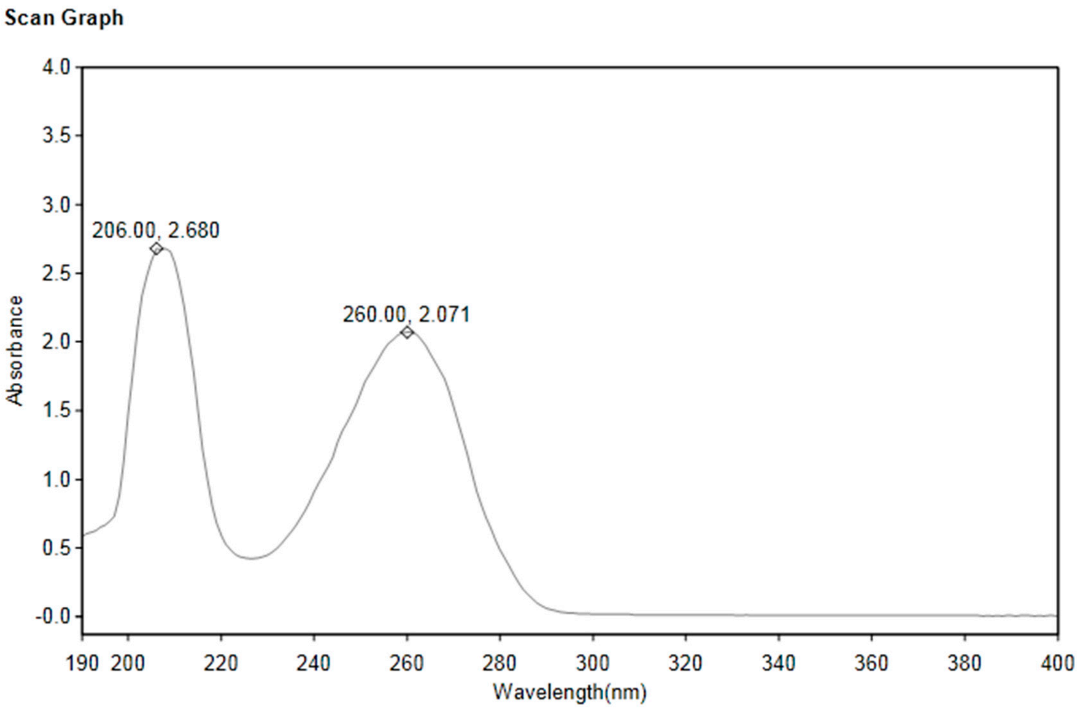

Results Table - scan017,1-111,Cycle01

nm	A	**Peak Pick Method**
206.00	2.680	Find 8 Peaks Above -3.0000 A
260.00	2.071	Start Wavelength190.00 nm
		Stop Wavelength400.00 nm
		Sort By Wavelength
Sensitivity	Medium	

Figure S6. UV spectrum of **1**

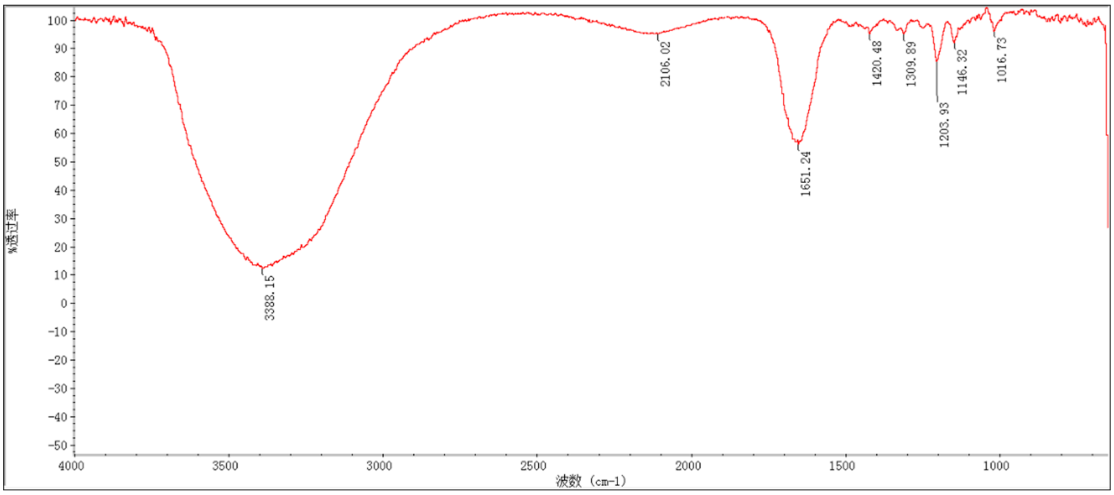

Figure S7. IR spectrum of **1**

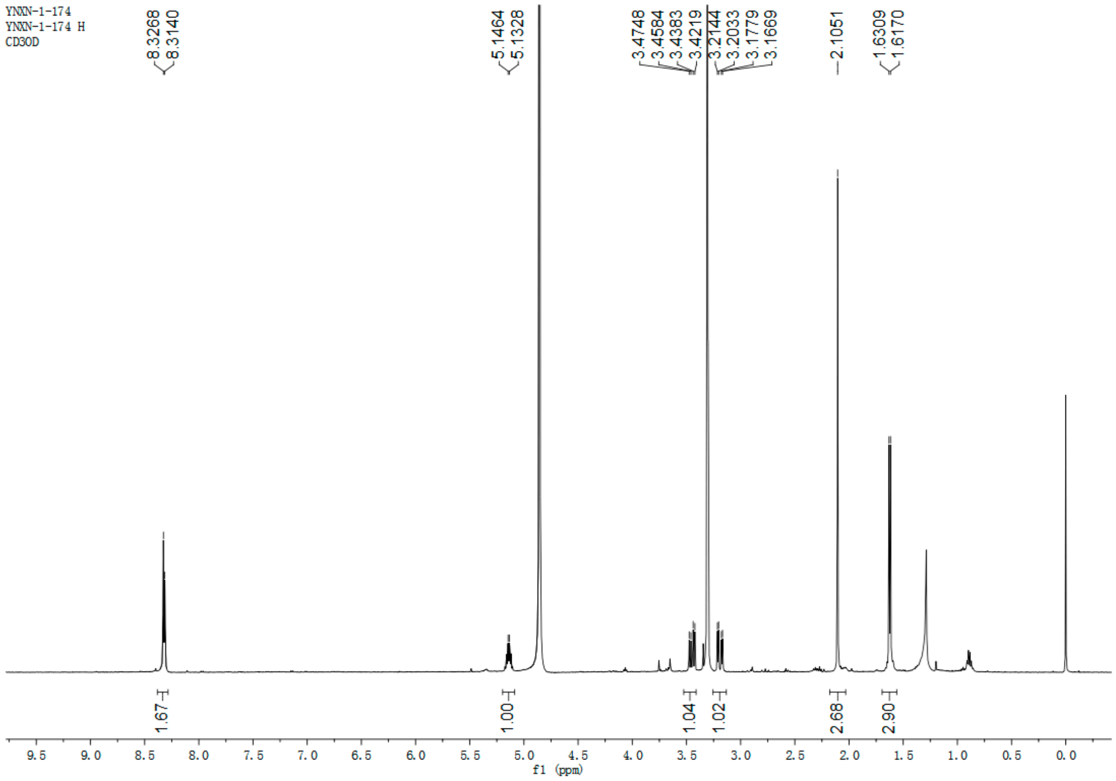

Figure S8. ^{1}H NMR spectrum (500MHz, CD_3OD) of **2**

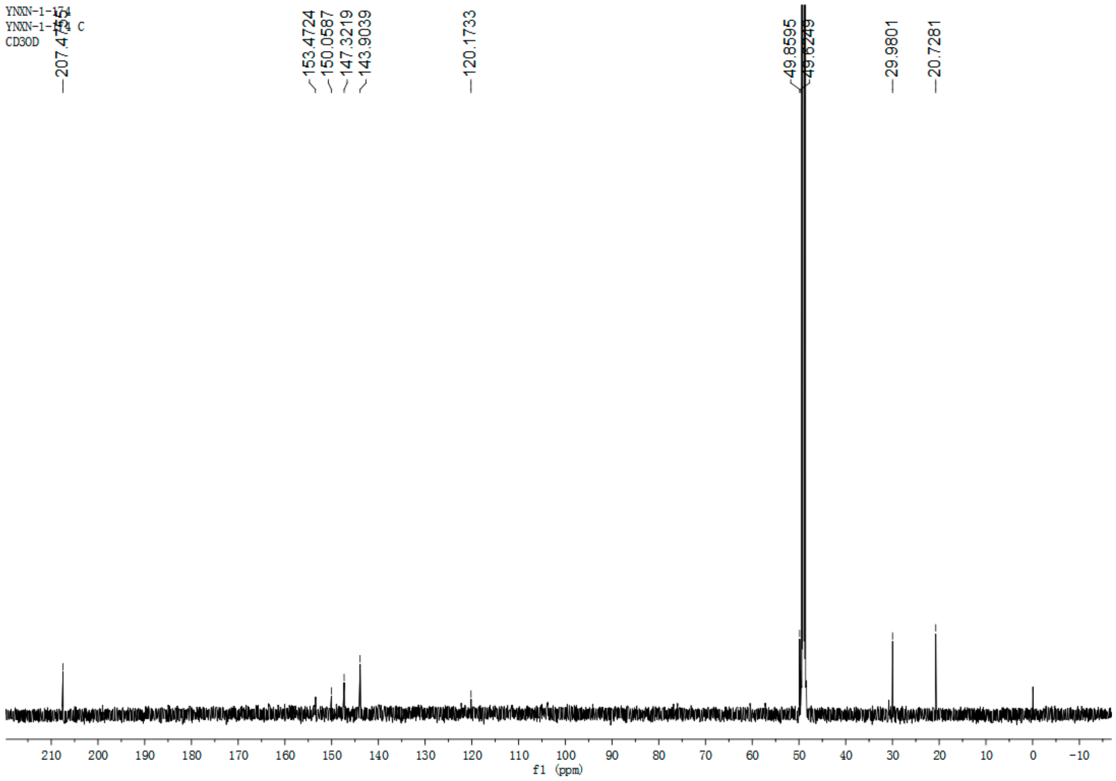

Figure S9. ^{13}C NMR spectrum (125MHz, CD_3OD) of **2**

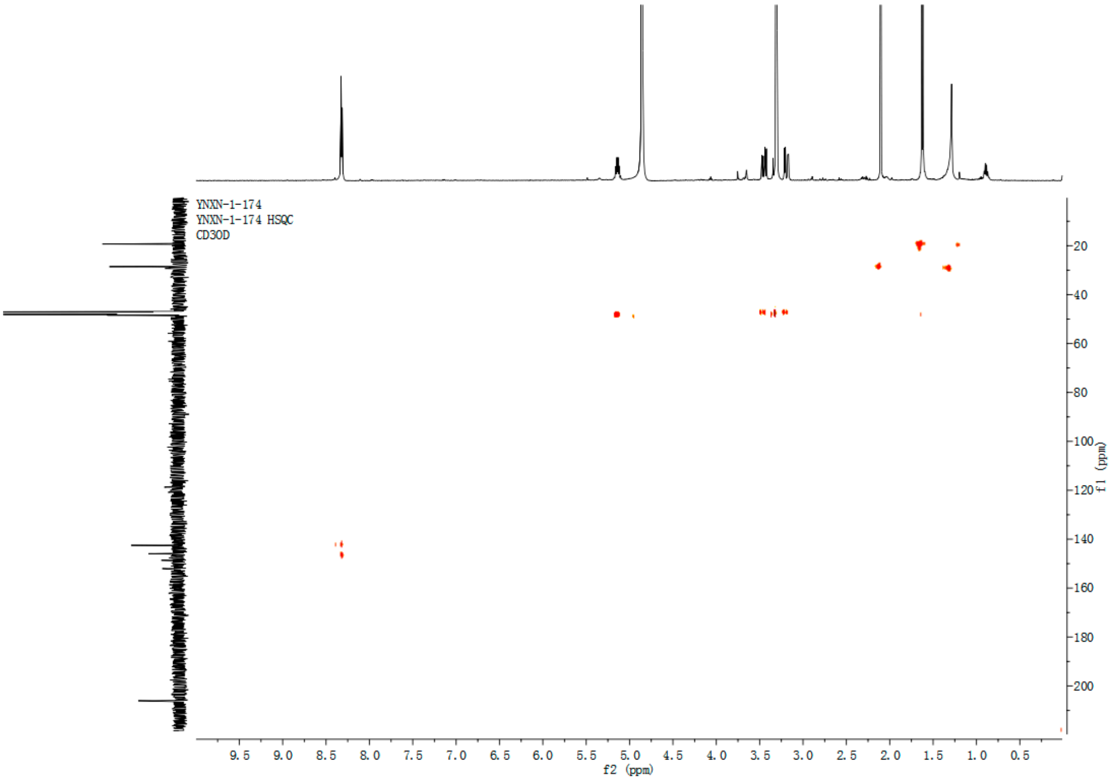

Figure S10. HSQC spectrum of **2**

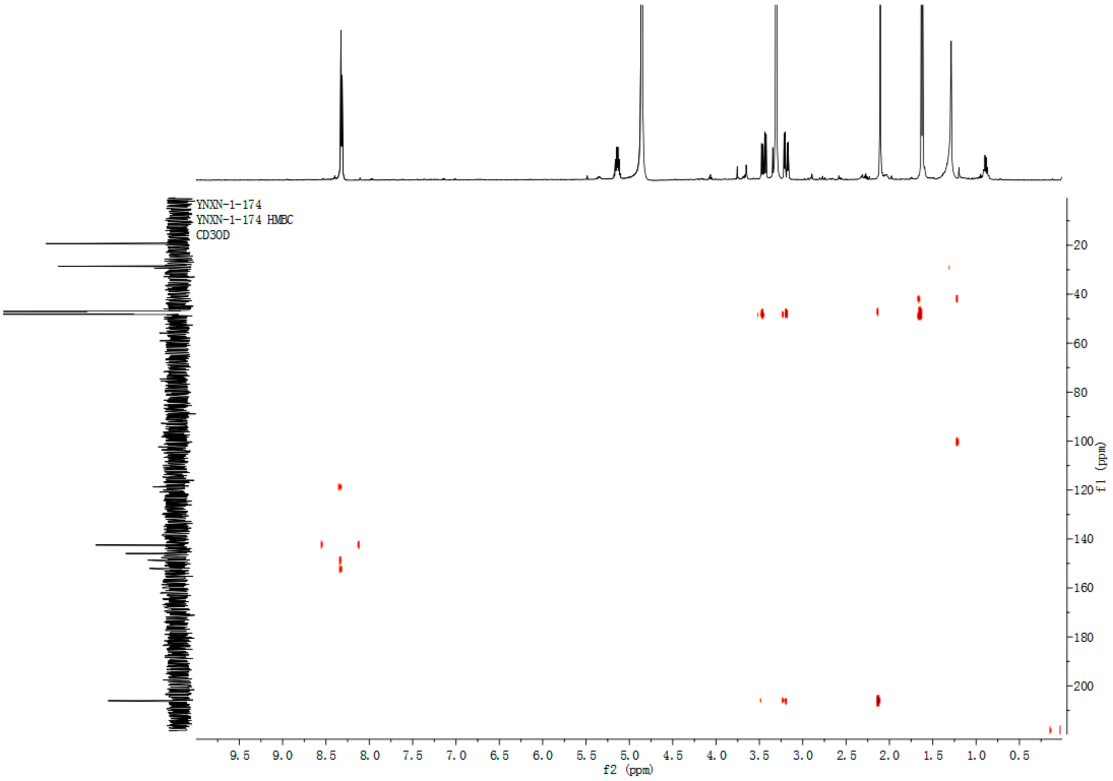

Figure S11. HMBC spectrum of **2**

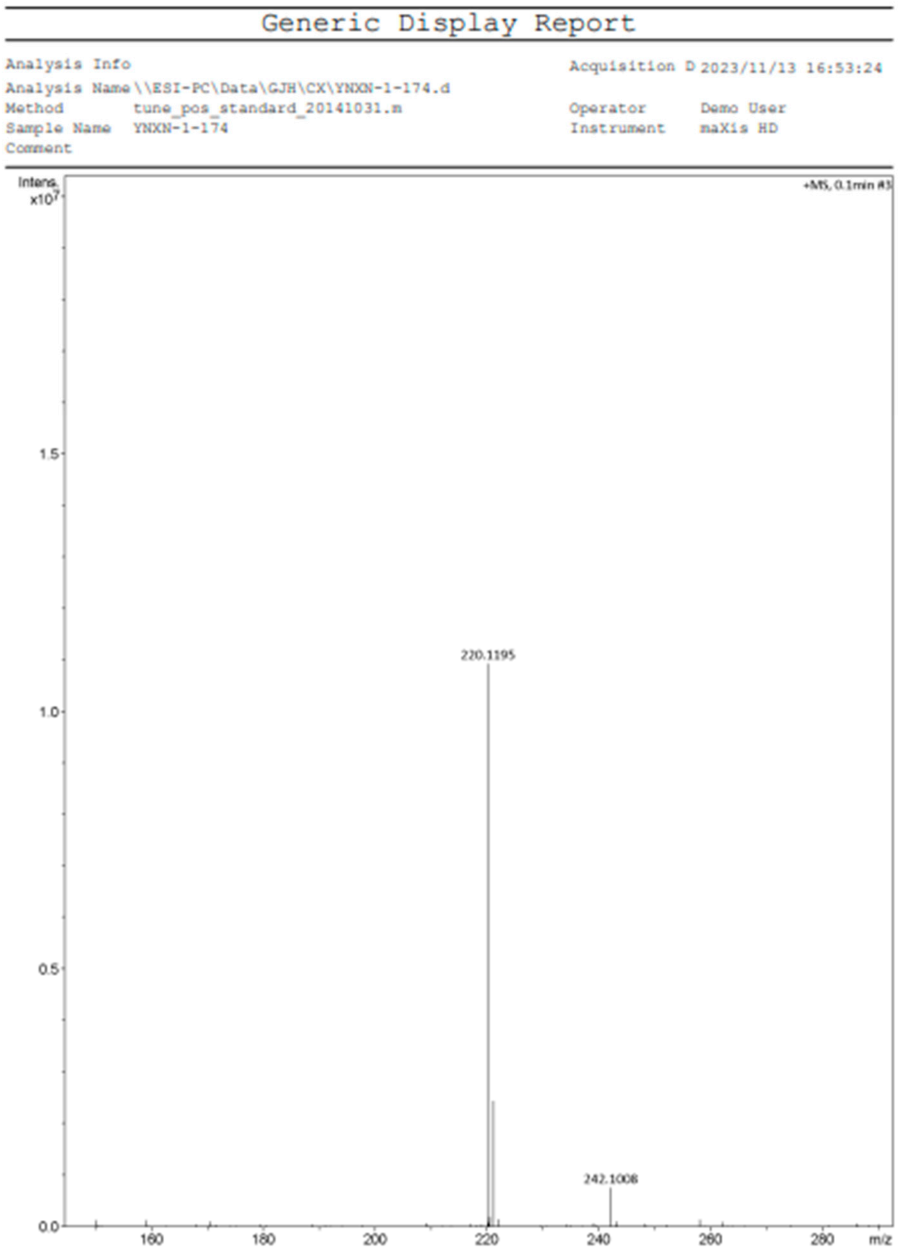

Figure S12. HR-ESI-MS spectrum of **2**

Scan Graph

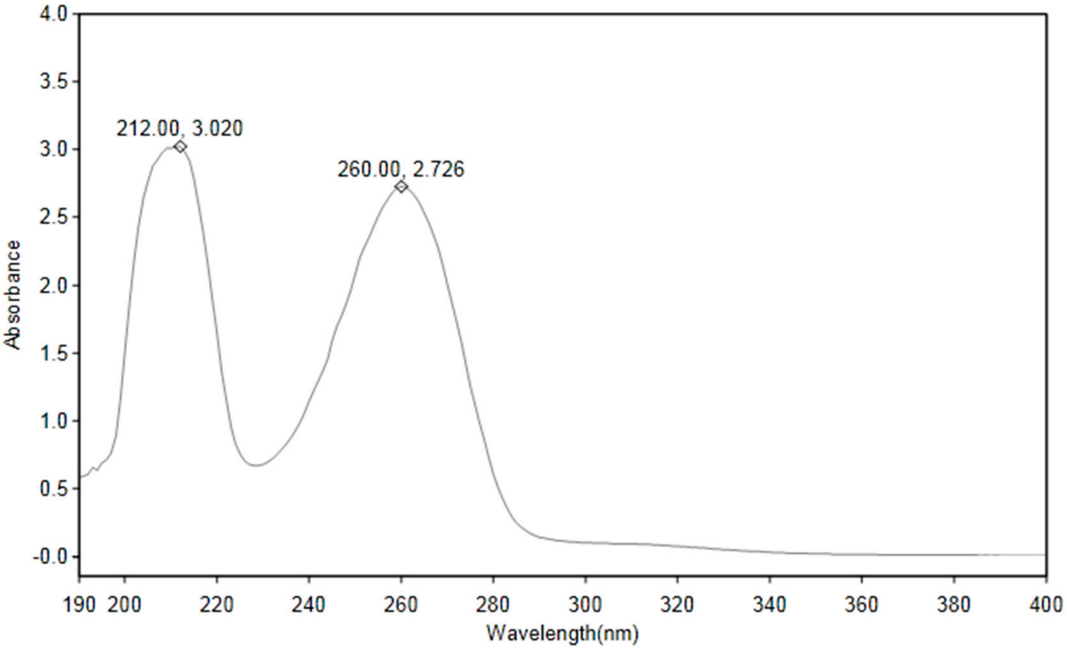

Results Table - scan008,N-1-174,Cycle01

nm	A	**Peak Pick Method**
212.00	3.020	Find 8 Peaks Above -3.0000 A
260.00	2.726	Start Wavelength190.00 nm
		Stop Wavelength400.00 nm
		Sort By Wavelength
Sensitivity	Auto	

Figure S13. UV spectrum of **2**

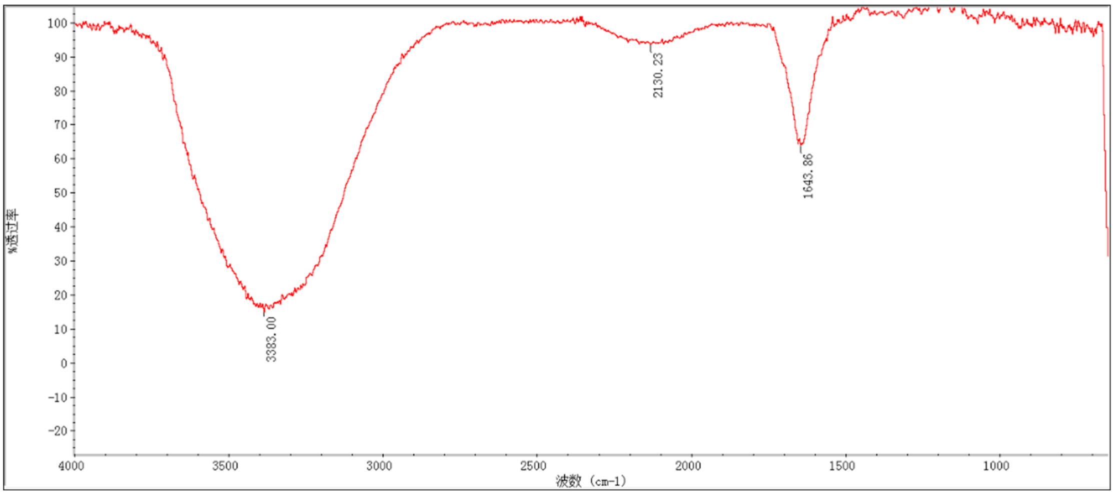

Figure S14. IR spectrum of **2**

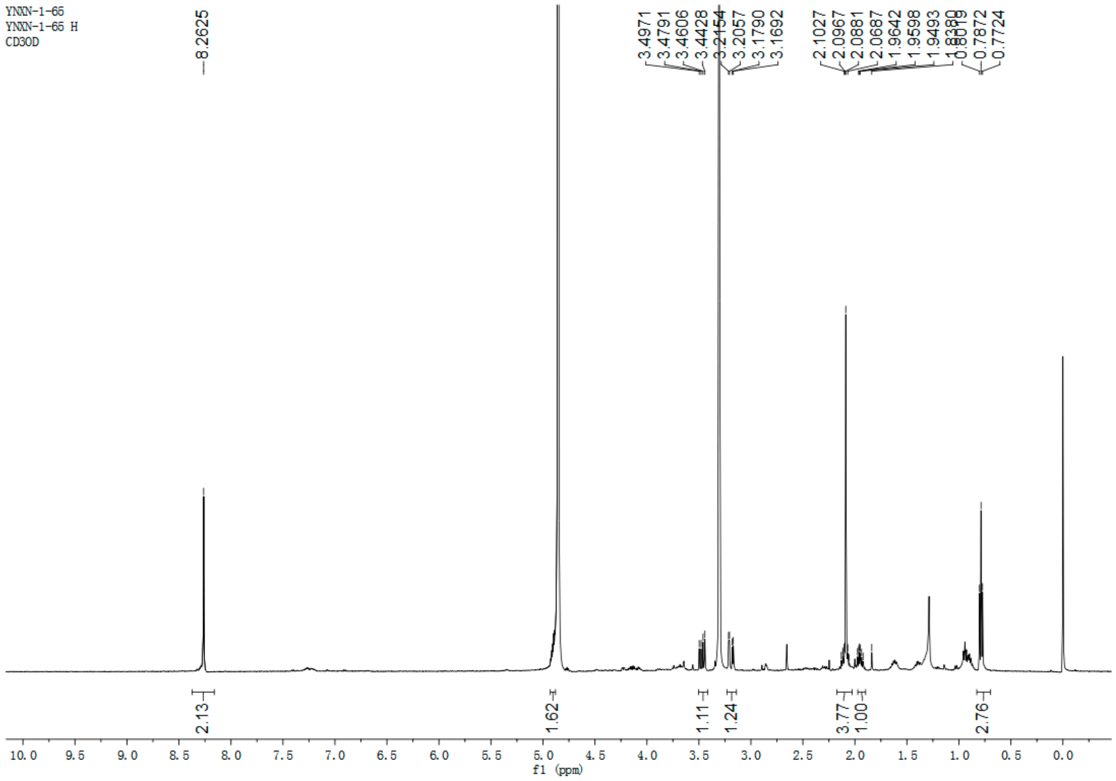

Figure S15. ^{1}H NMR spectrum (500MHz, CD_3OD) of **3**

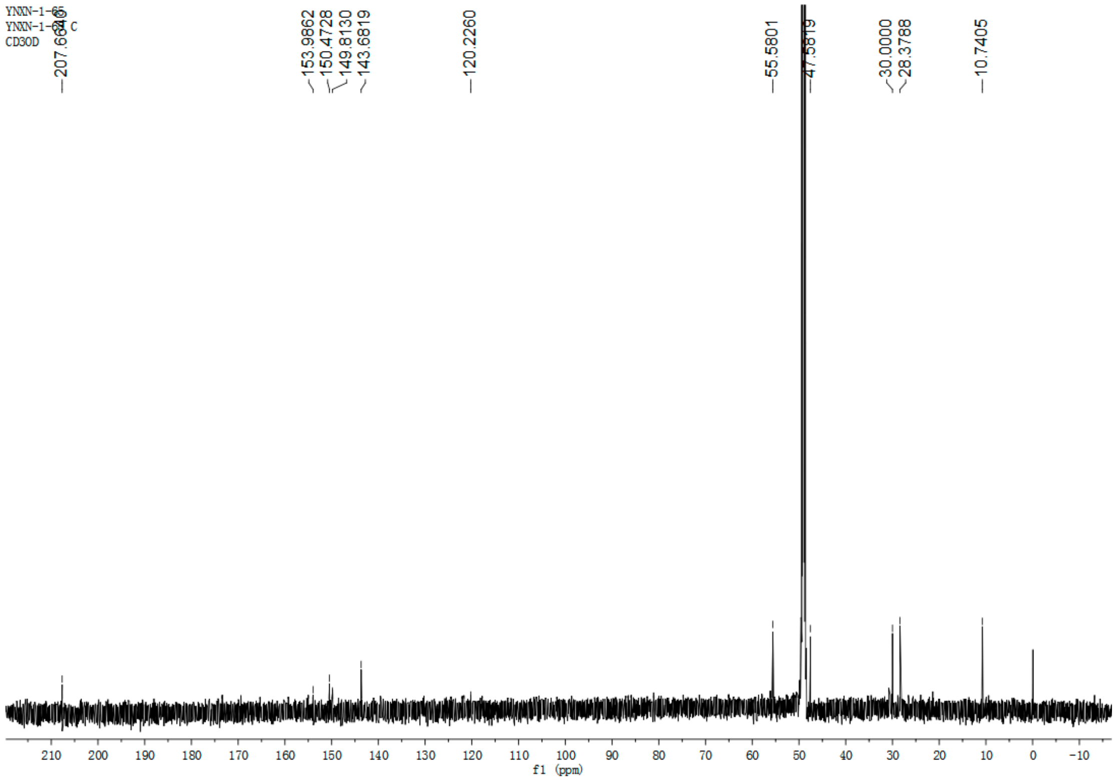

Figure S16. ^{13}C NMR spectrum (125MHz, CD_3OD) of **3**

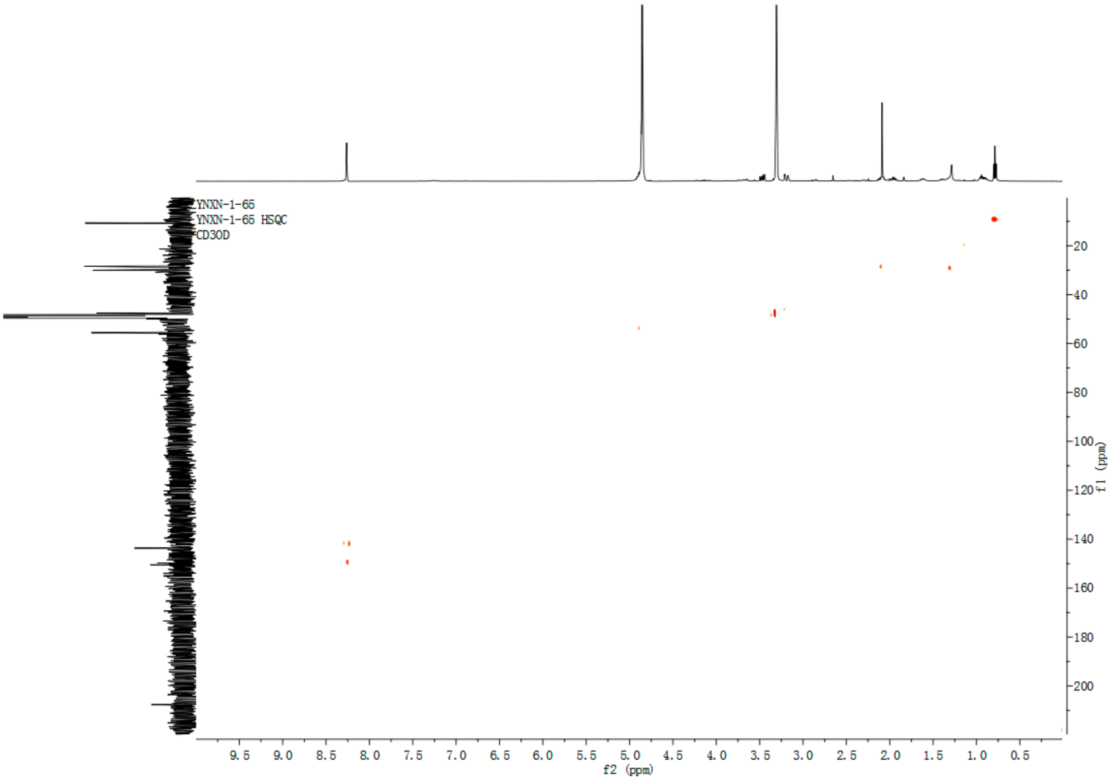

Figure S17. HSQC spectrum of **3**

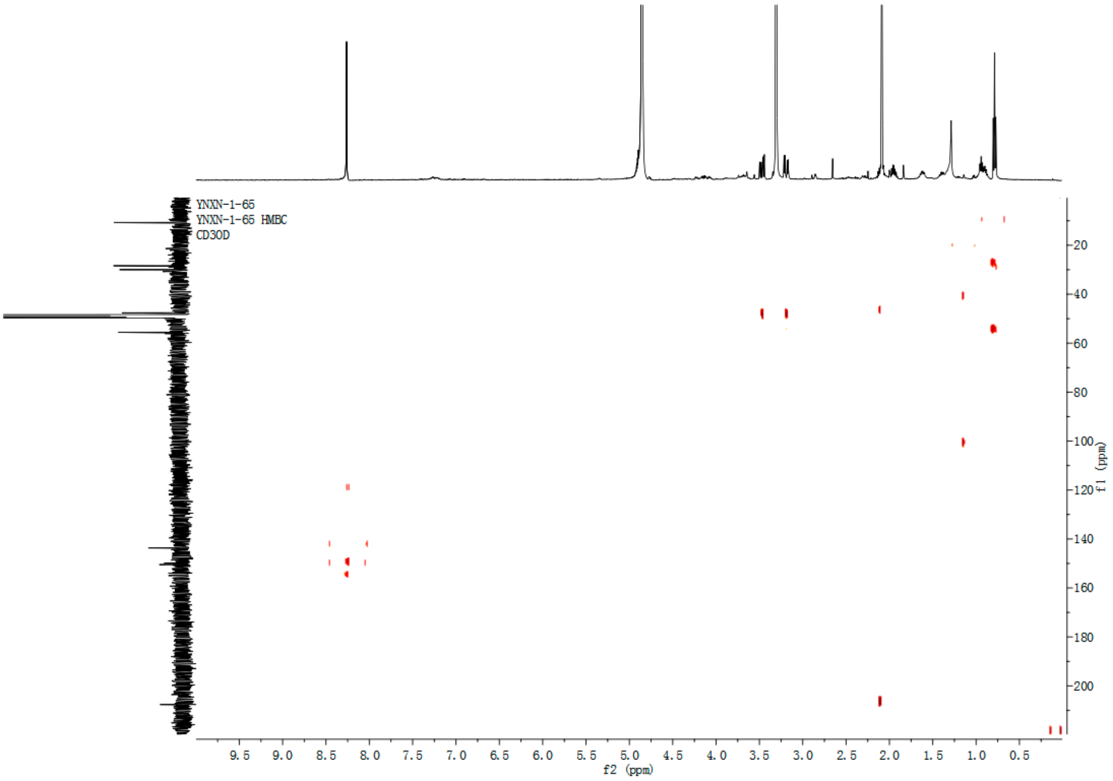

Figure S18. HMBC spectrum of **3**

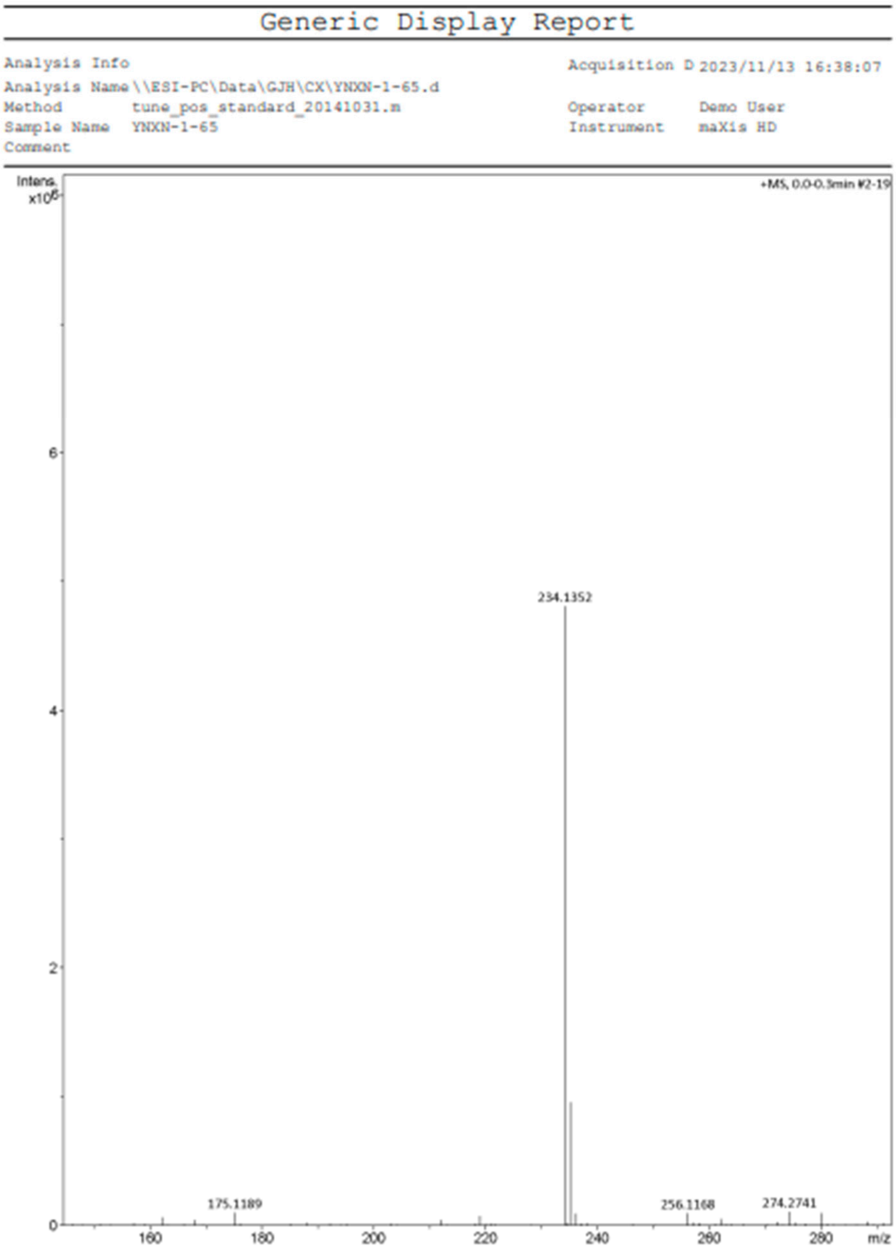

Figure S19. HR-ESI-MS spectrum of **3**

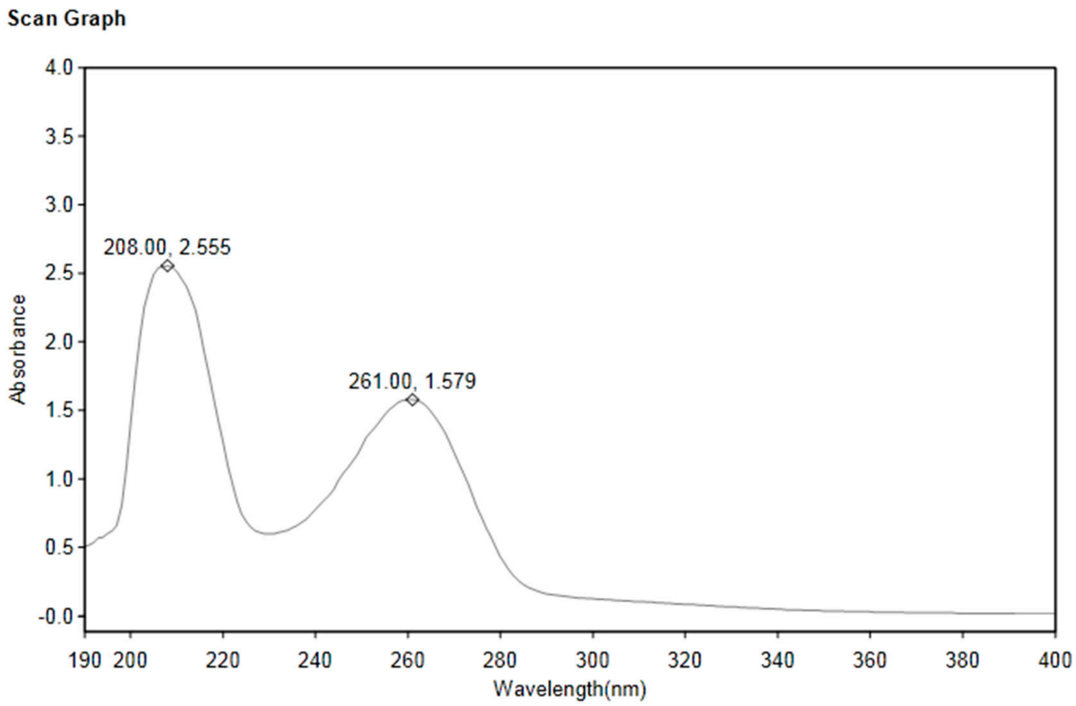

Results Table - scan007,N-1-65,Cycle01

nm	A	Peak Pick Method
208.00	2.555	Find 8 Peaks Above -3.0000 A
261.00	1.579	Start Wavelength190.00 nm
		Stop Wavelength400.00 nm
		Sort By Wavelength
Sensitivity	Auto	

Figure S20. UV spectrum of **3**

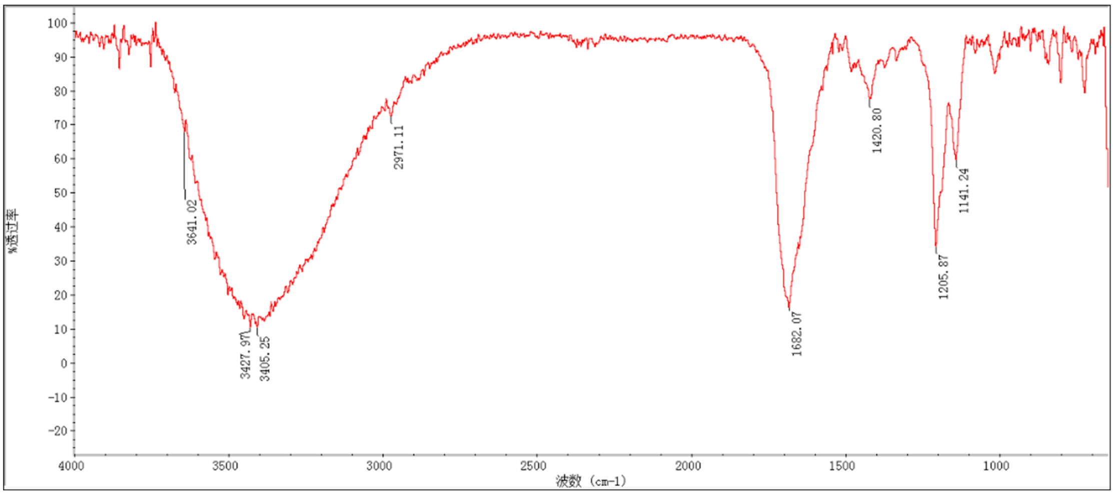

Figure S21. IR spectrum of **3**

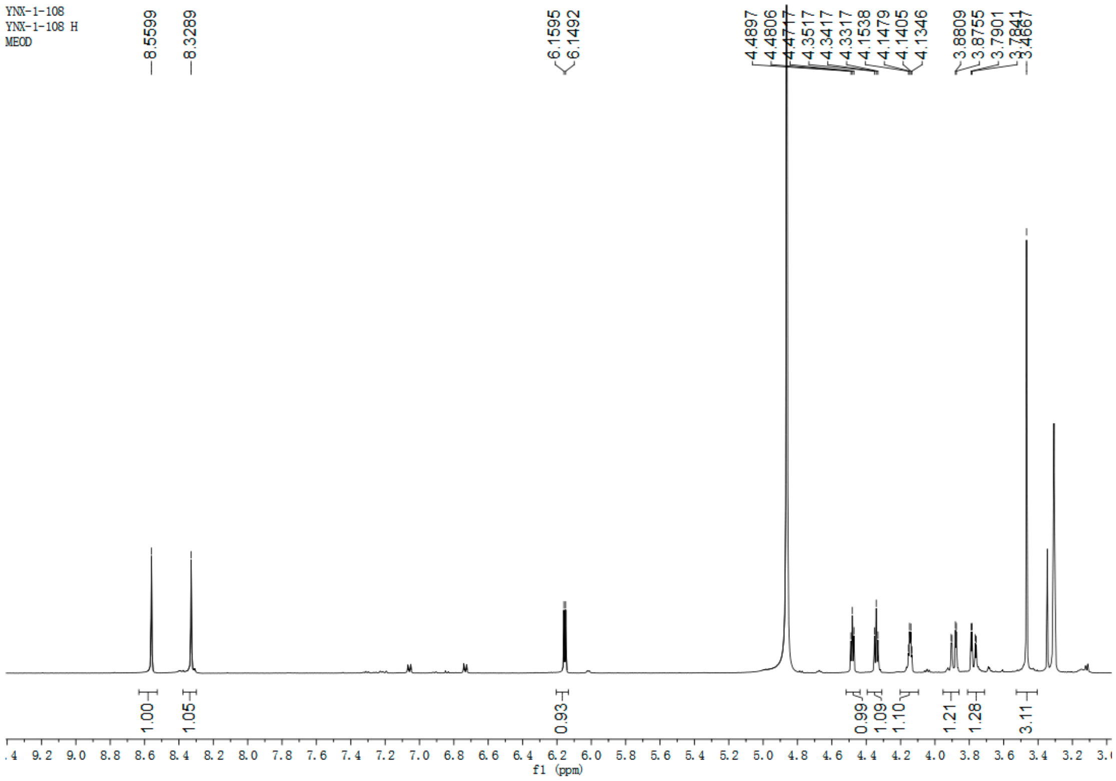

Figure S22. ^{1}H NMR spectrum (500MHz, CD_3OD) of **4**

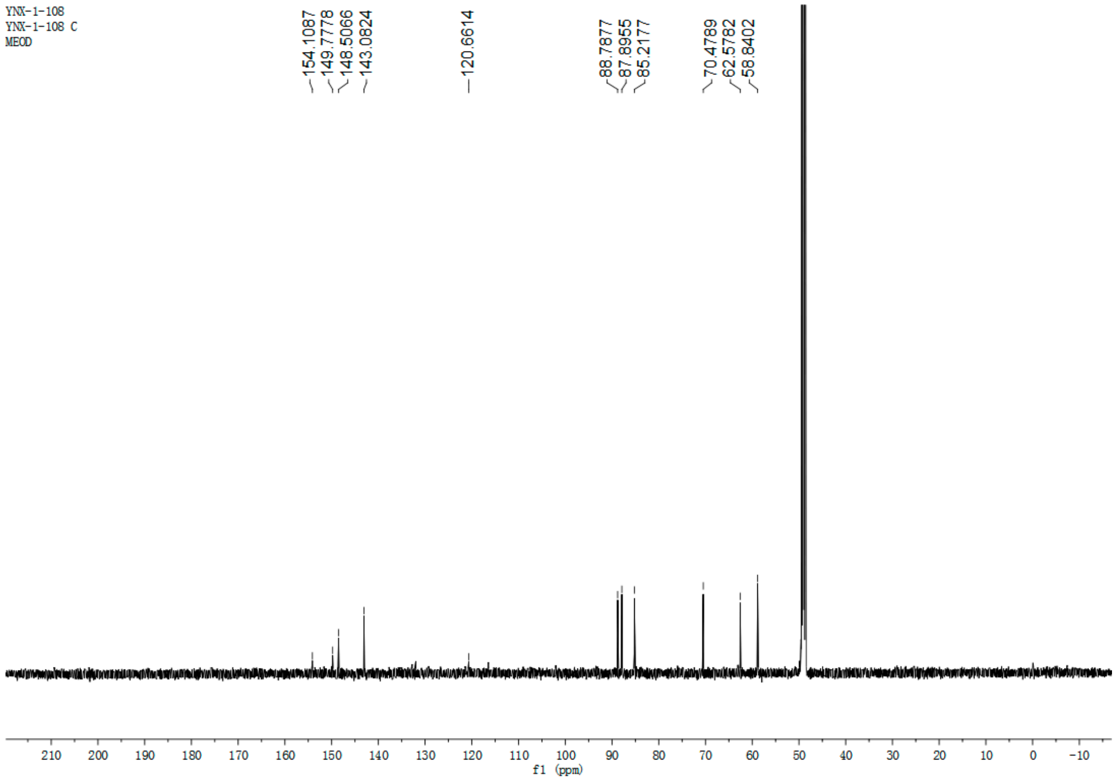

Figure S23. ^{13}C NMR spectrum (125MHz, CD_3OD) of **4**

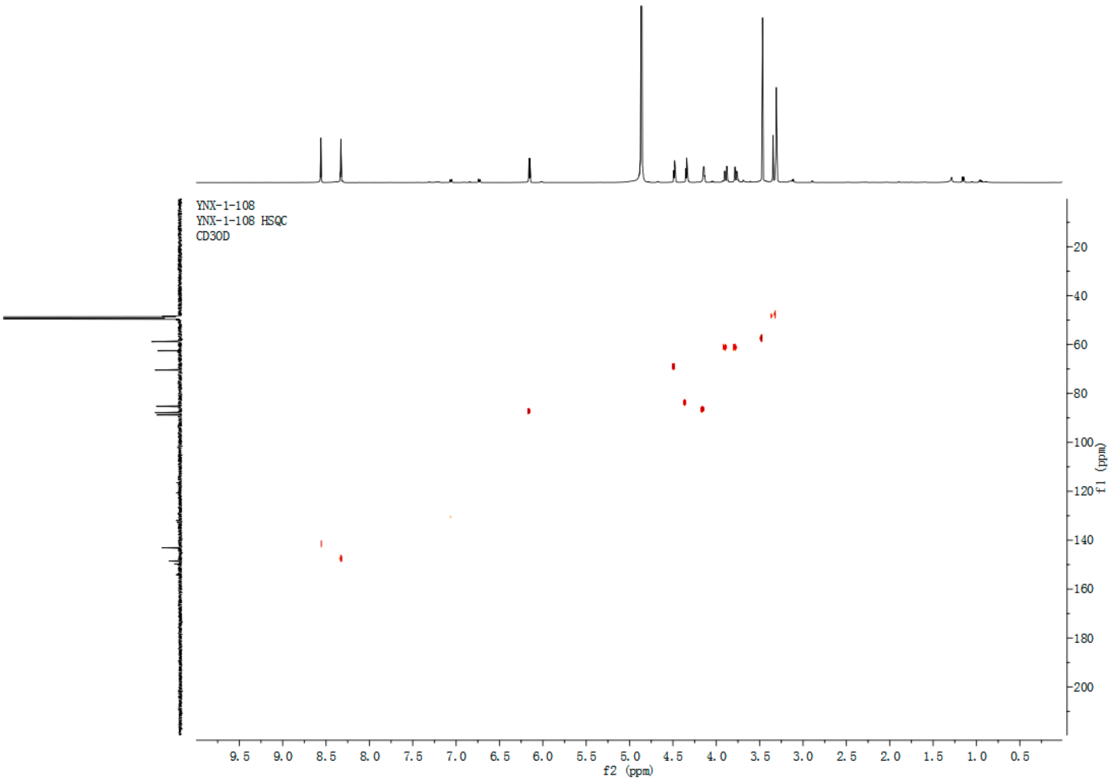

Figure S24. HSQC spectrum of **4**

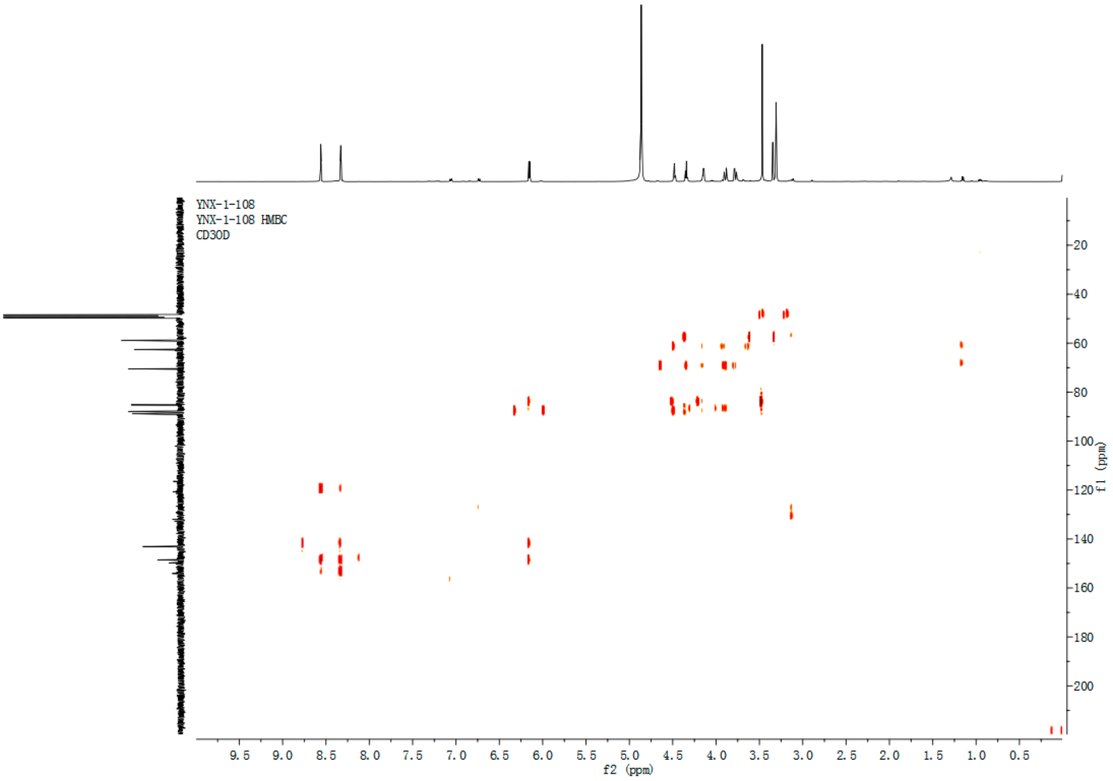

Figure S25. HMBC spectrum of **4**

Generic Display Report

Analysis Info		Acquisition D	2023/9/6 20:50:35
Analysis Name	\\ESI-PC\Data\GJH\CX\YNX-1-108.d		
Method	tune_pos_standard_20141031.m	Operator	Demo User
Sample Name	YNX-1-108	Instrument	maXis HD
Comment			

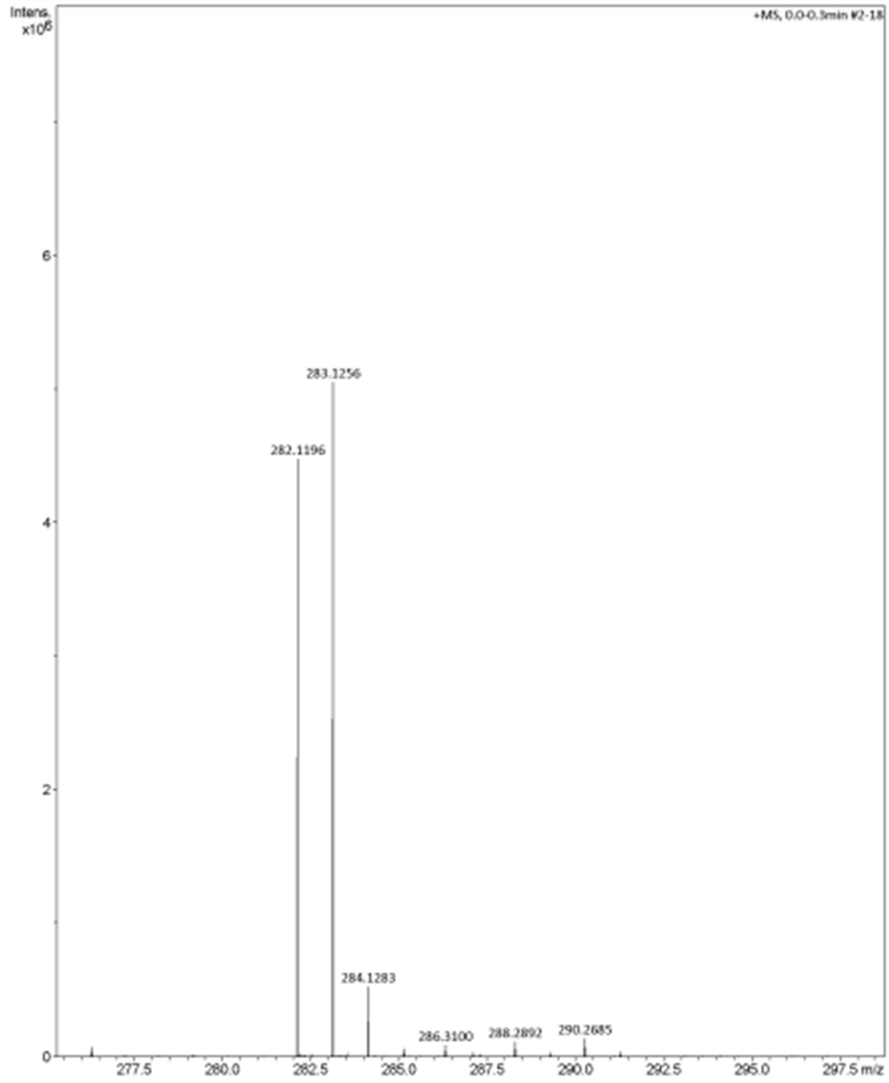

Figure S26. HR-ESI-MS spectrum of **4**

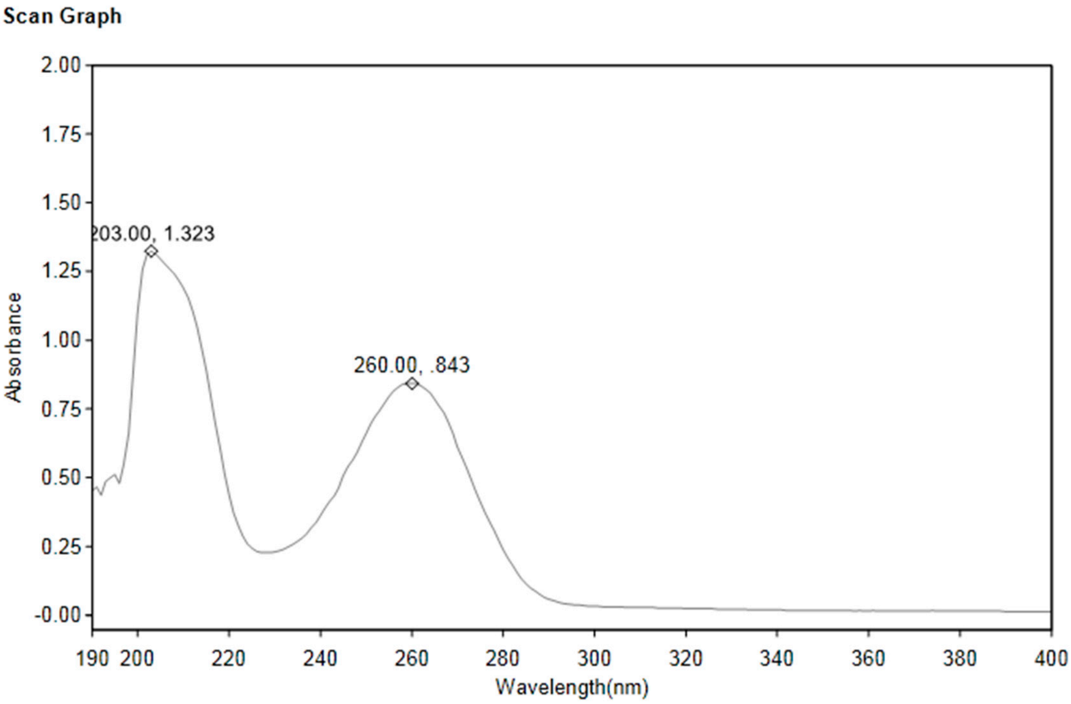

Results Table - scan026,1-108,Cycle01

nm	A	**Peak Pick Method**
203.00	1.323	Find 8 Peaks Above -3.0000 A
260.00	.843	Start Wavelength190.00 nm
		Stop Wavelength400.00 nm
		Sort By Wavelength
Sensitivity	Auto	

Figure S27. UV spectrum of **4**

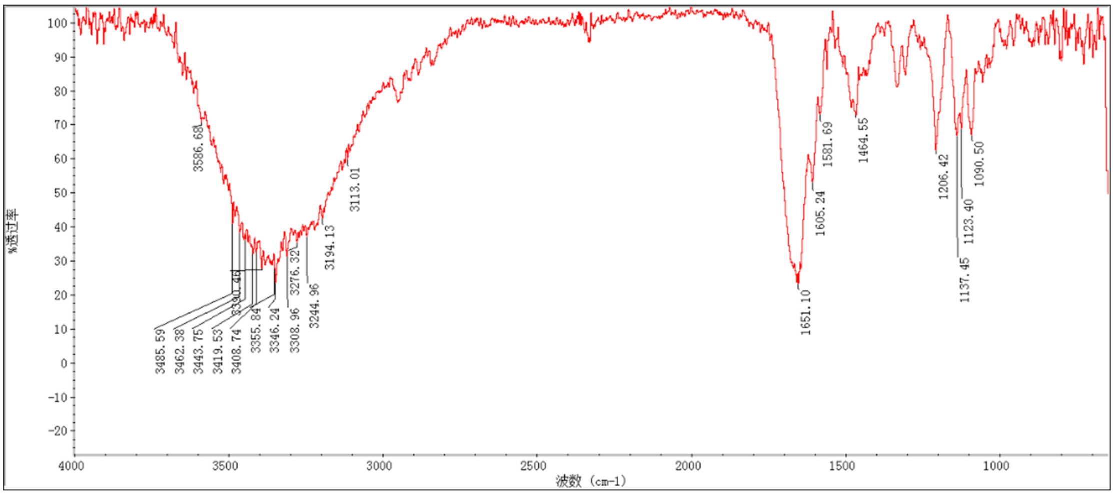

Figure S28. IR spectrum of **4**

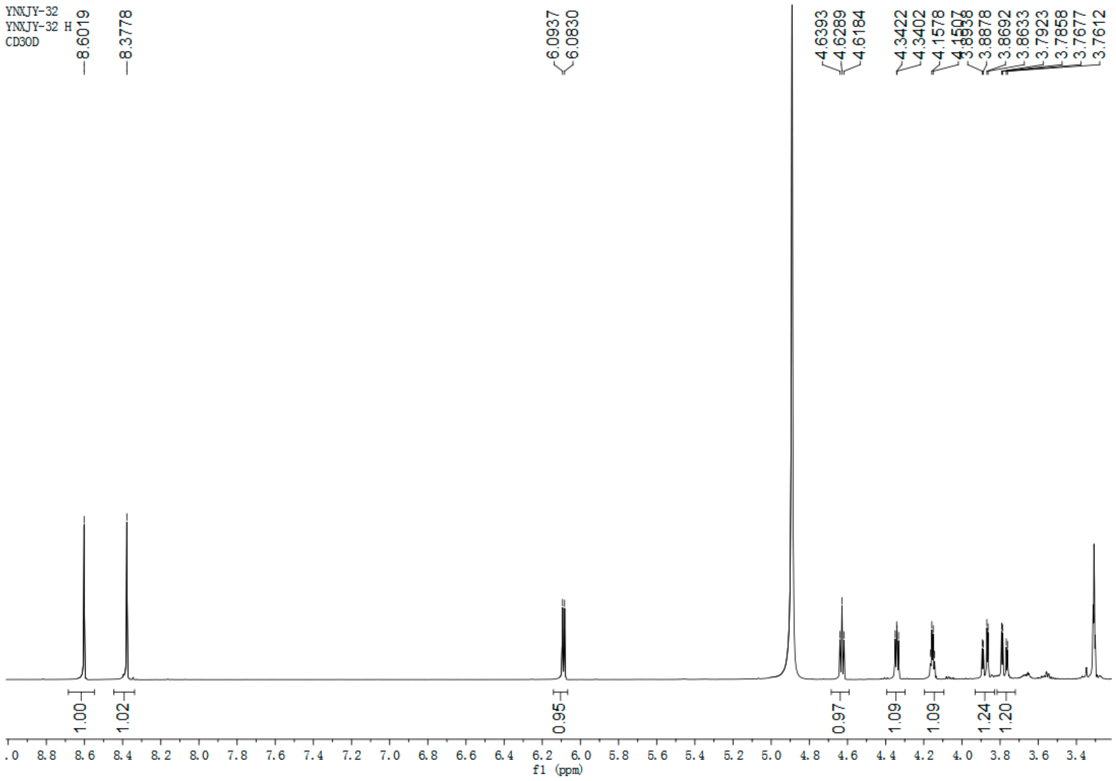

Figure S29. ^{1}H NMR spectrum (500MHz, CD_3OD) of **5**

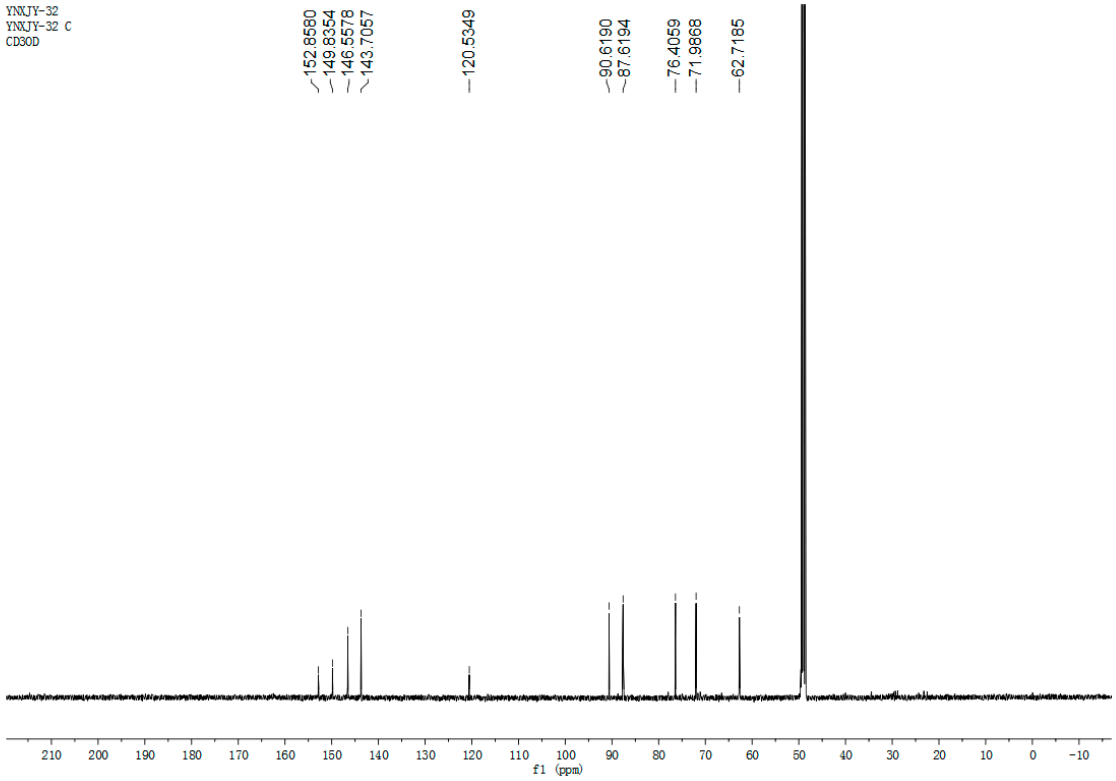

Figure S30. ^{13}C NMR spectrum (125MHz, CD_3OD) of **5**

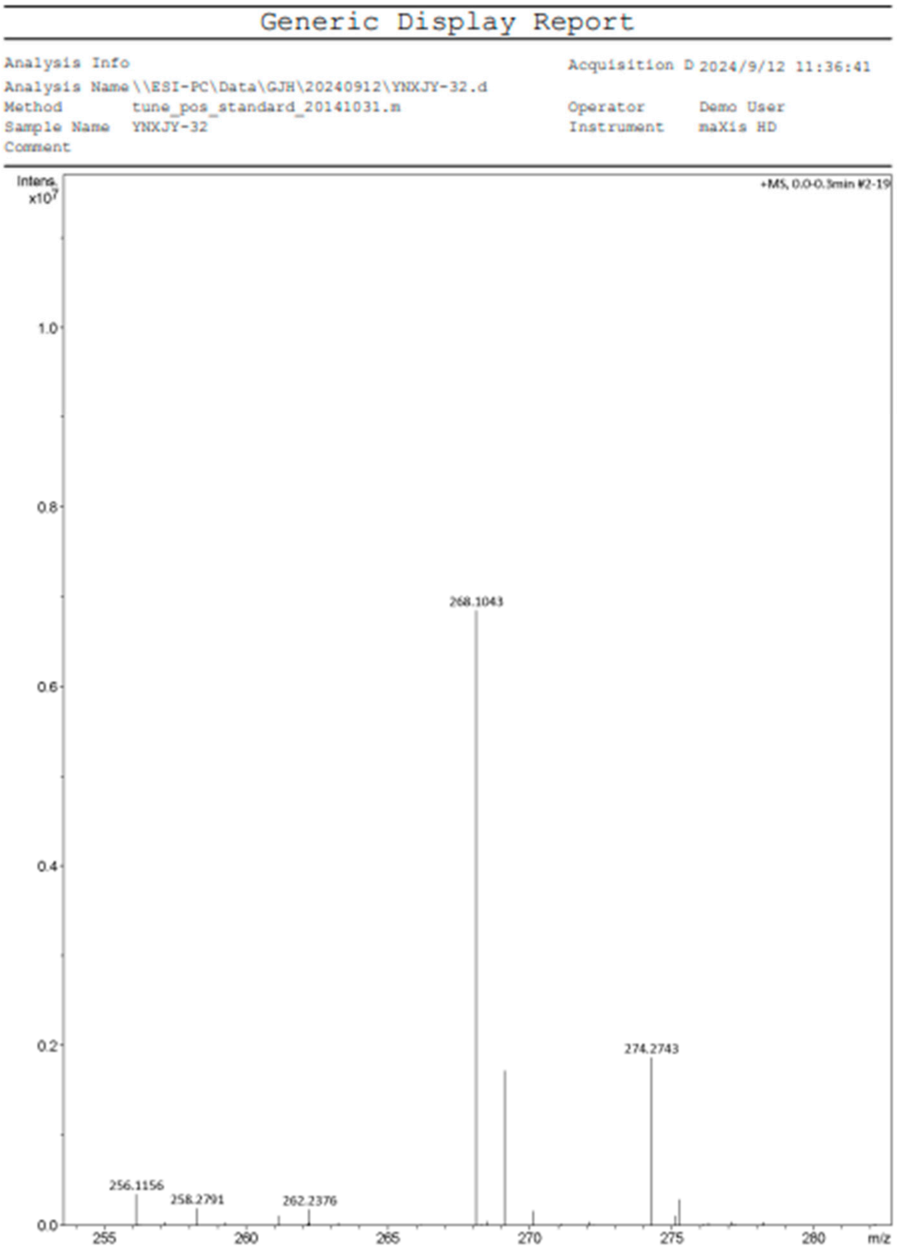

Figure S31. HR-ESI-MS spectrum of **5**

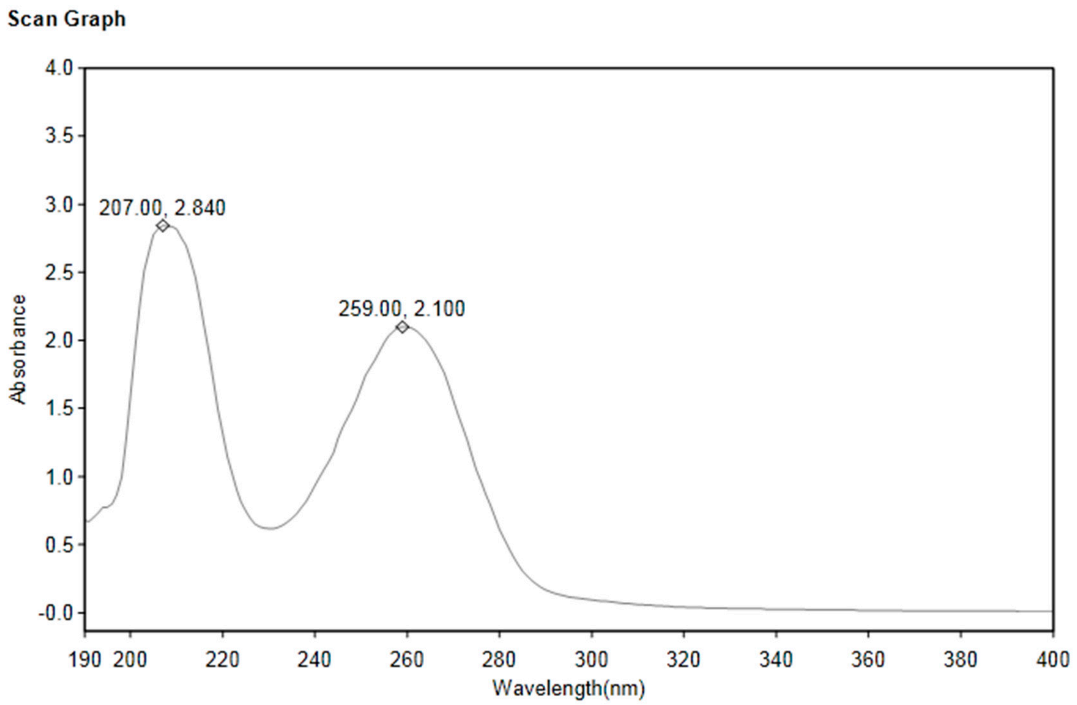

Results Table - scan015,YNXJY-32,Cycle01

nm	A	**Peak Pick Method**
207.00	2.840	Find 8 Peaks Above -3.0000 A
259.00	2.100	Start Wavelength190.00 nm
		Stop Wavelength400.00 nm
		Sort By Wavelength
Sensitivity	Medium	

Figure S32. UV spectrum of **5**

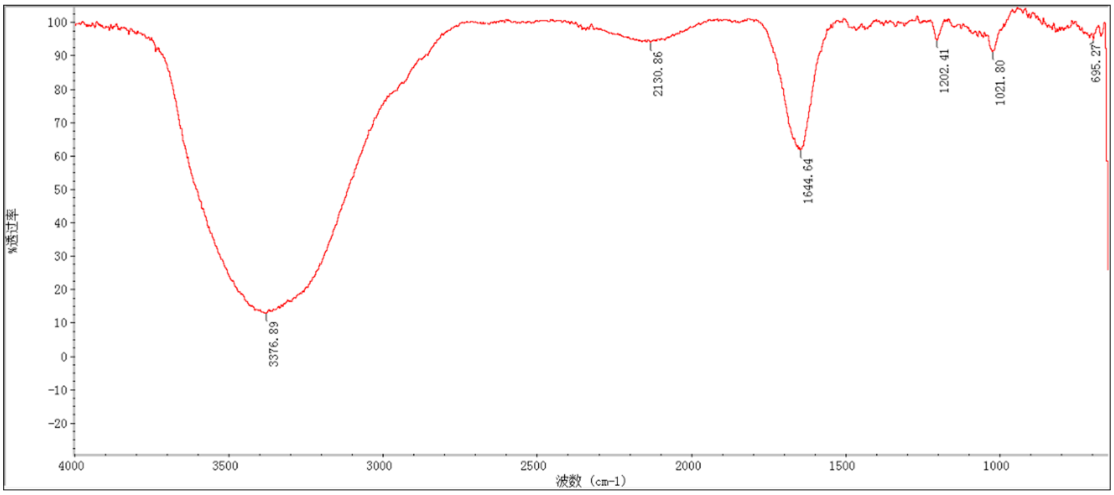

Figure S33. IR spectrum of **5**

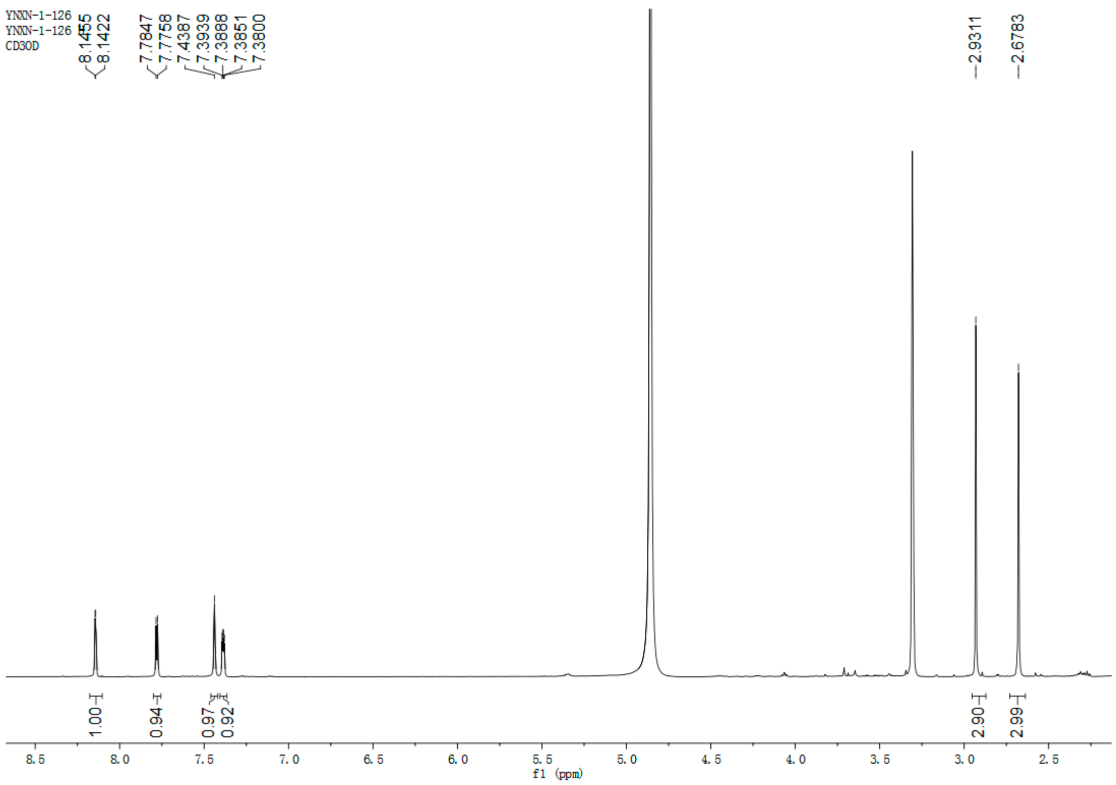

Figure S34. ^{1}H NMR spectrum (500MHz, CD_3OD) of **6**

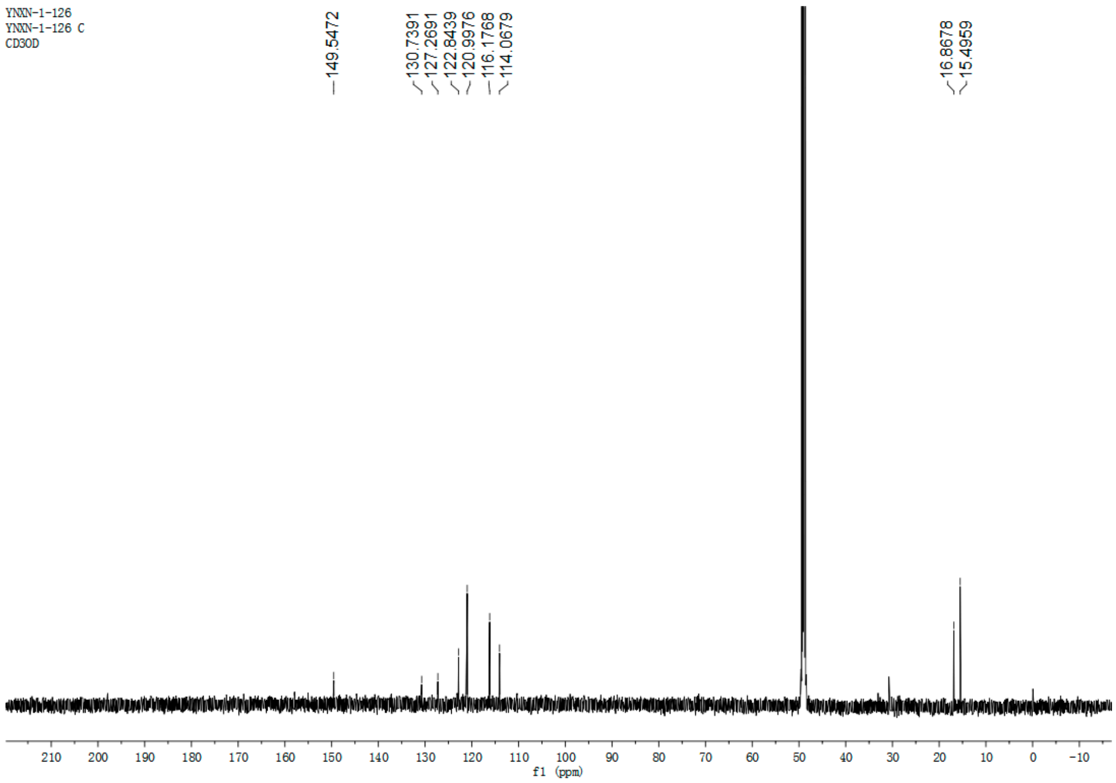

Figure S35. ^{13}C NMR spectrum (125MHz, CD_3OD) of **6**

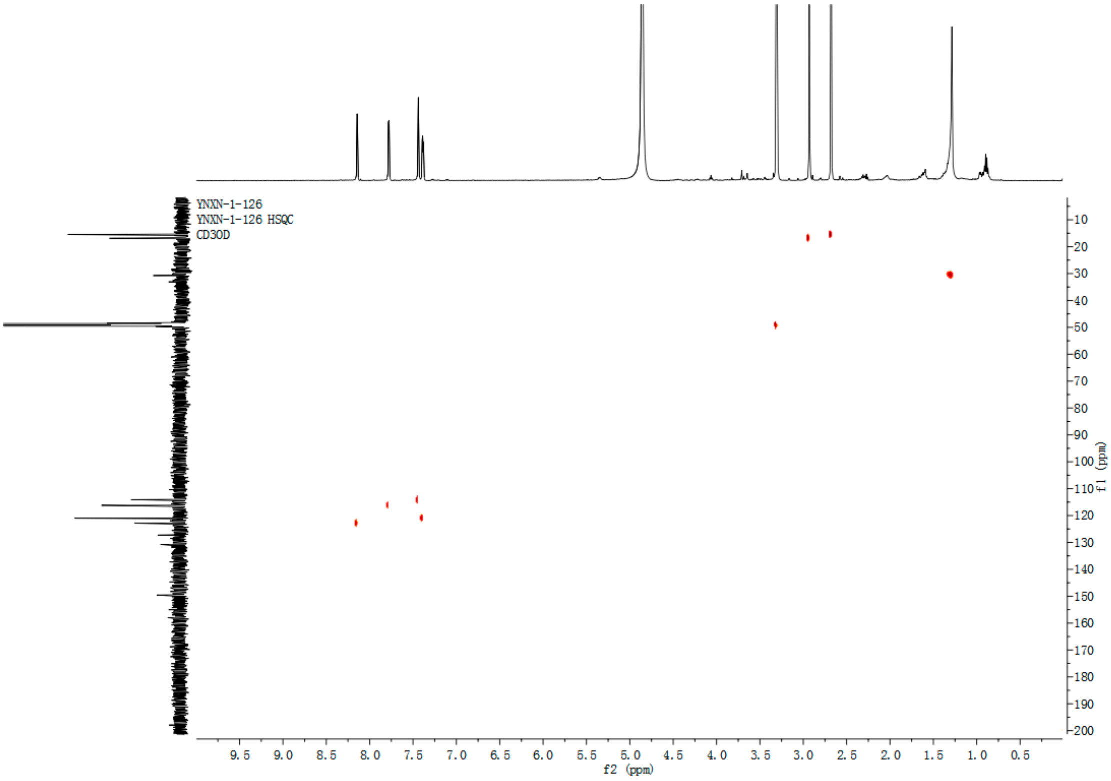

Figure S36. HSQC spectrum of **6**

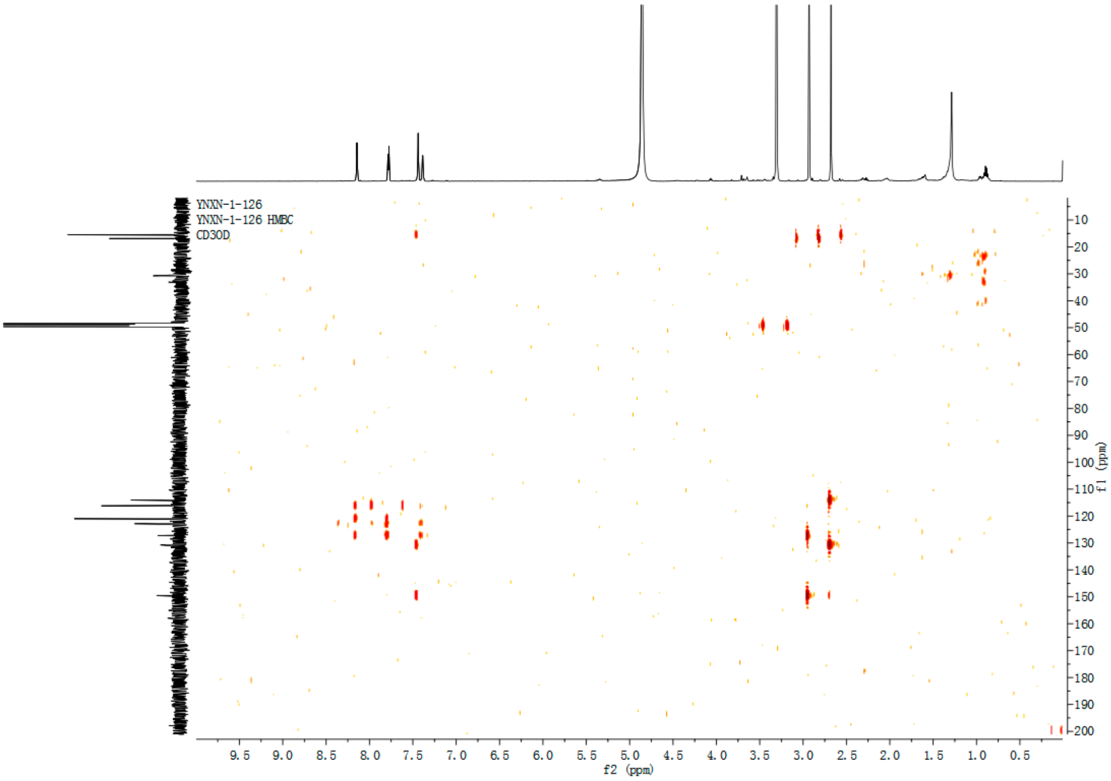

Figure S37. HMBC spectrum of **6**

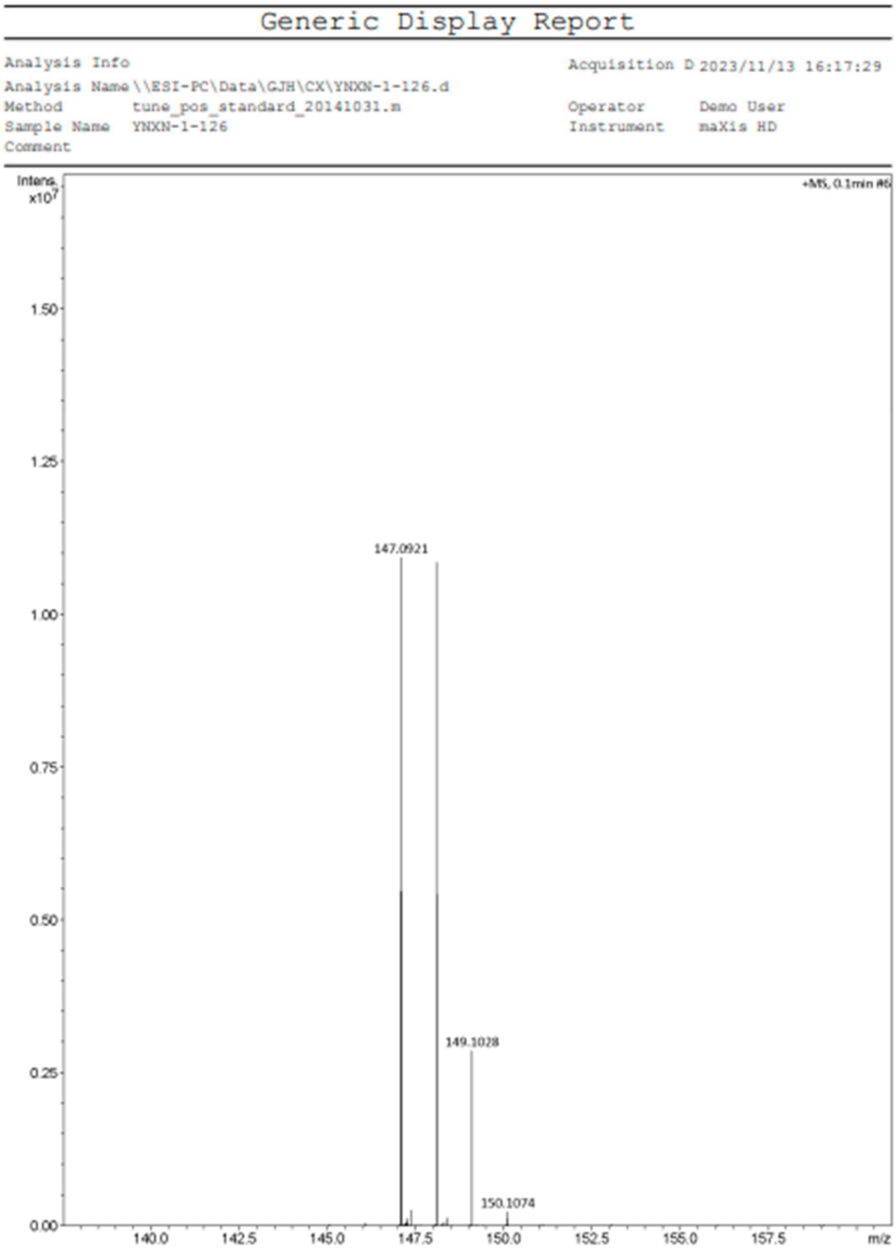

Figure S38. HR-ESI-MS spectrum of **6**

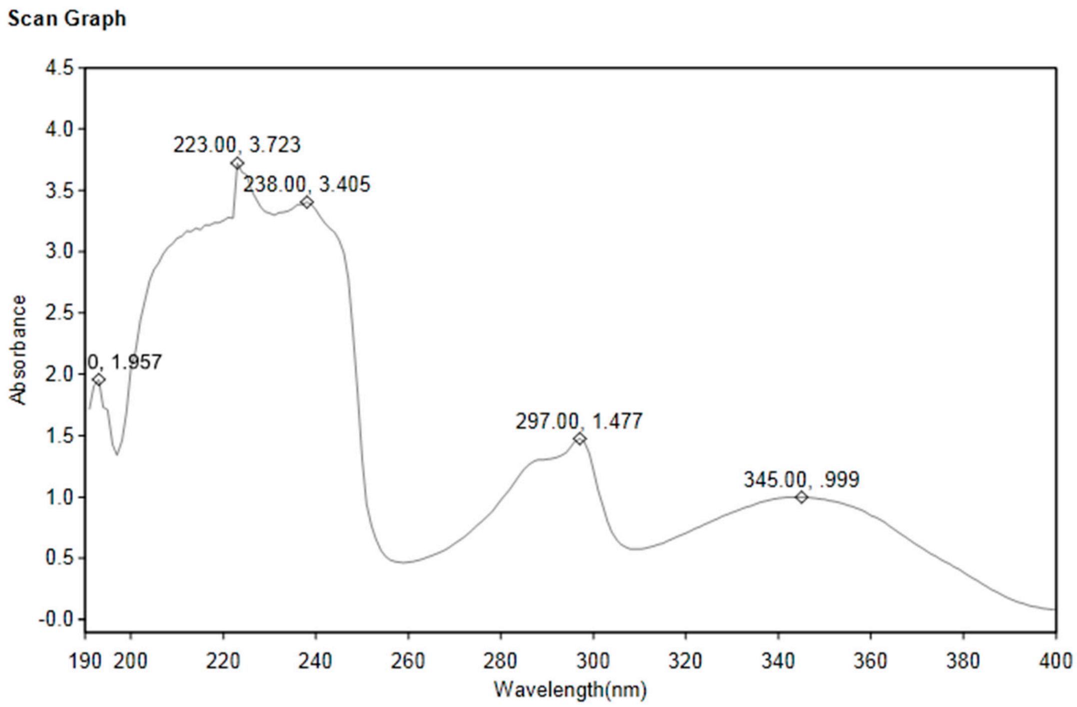

Results Table - scan023,N-126,Cycle01

nm	A	Peak Pick Method	
193.00	1.957	Find 8 Peaks Above -3.0000 A	
223.00	3.723	Start Wavelength190.00 nm	
238.00	3.405	Stop Wavelength400.00 nm	
297.00	1.477	Sort By Wavelength	
345.00	.999	Sensitivity	Auto

Figure S39. UV spectrum of **6**

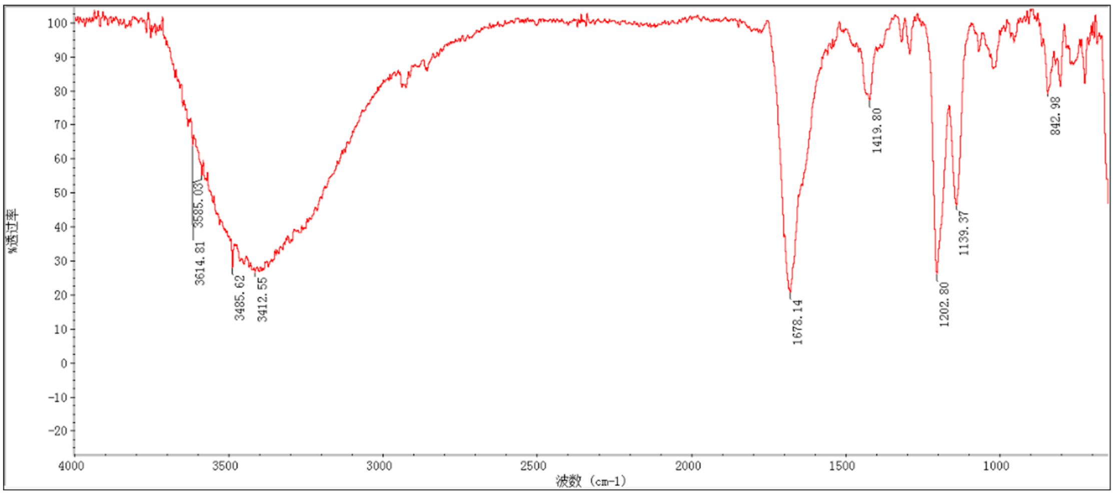

Figure S40. IR spectrum of **6**

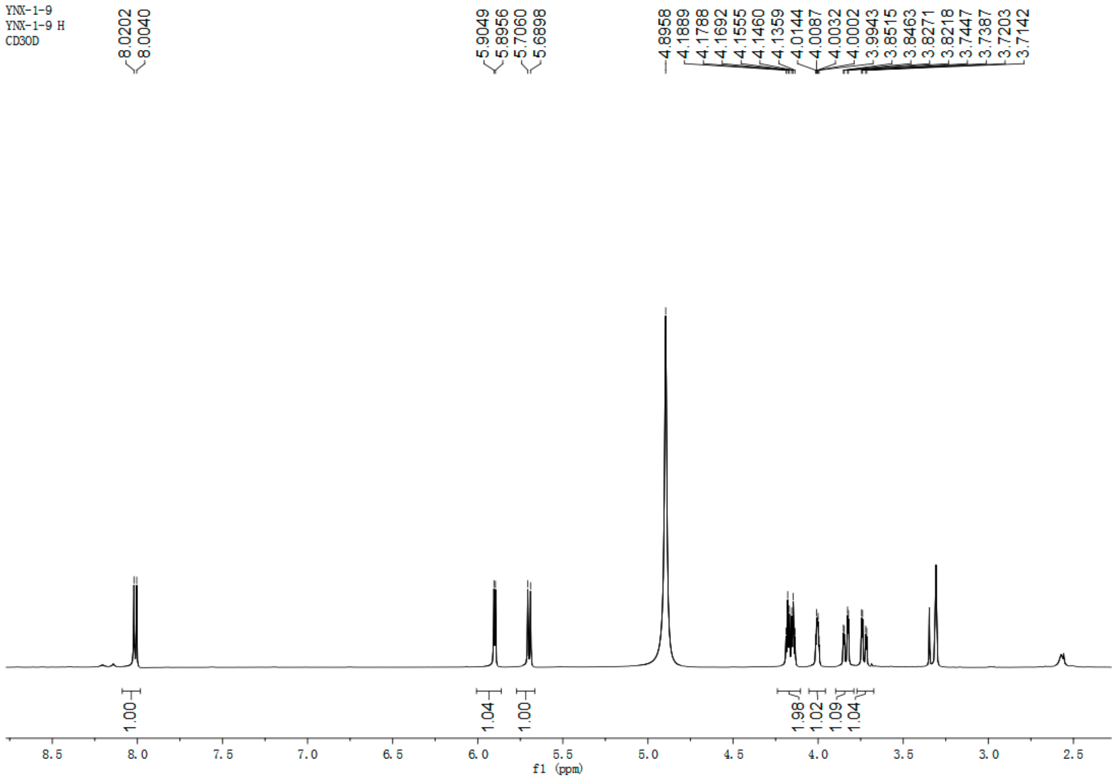

Figure S41. ^{1}H NMR spectrum (500MHz, CD_3OD) of **7**

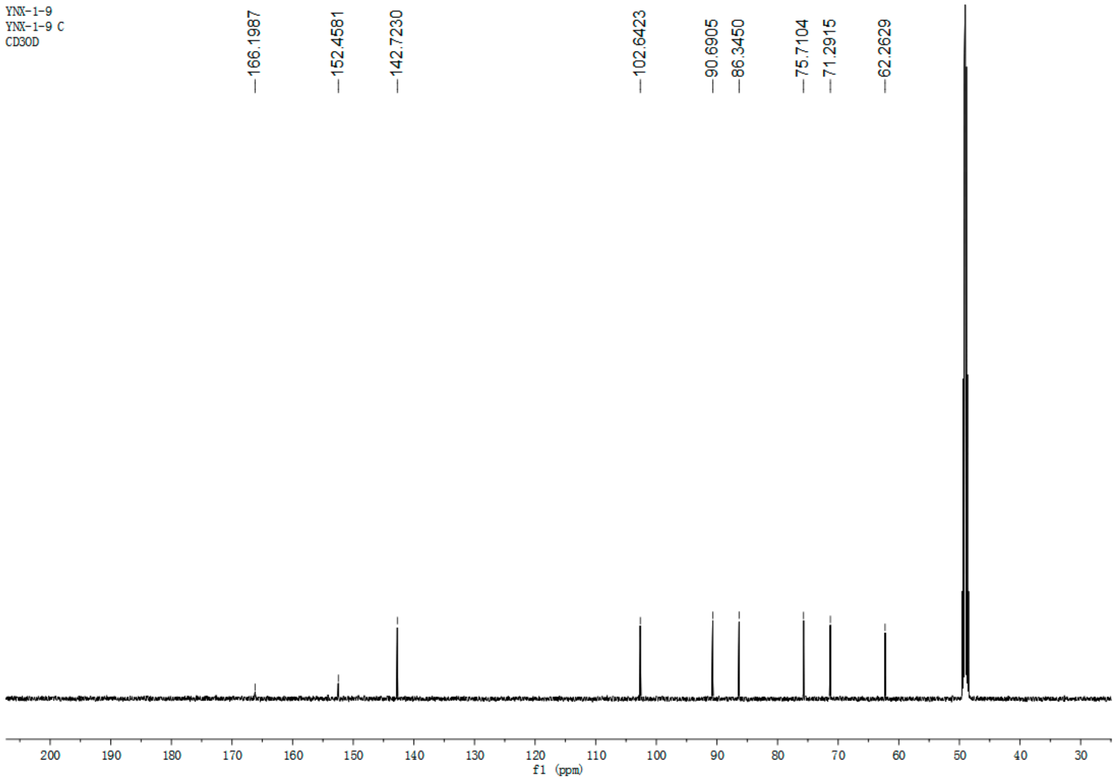

Figure S42. ^{13}C NMR spectrum (125MHz, CD_3OD) of **7**

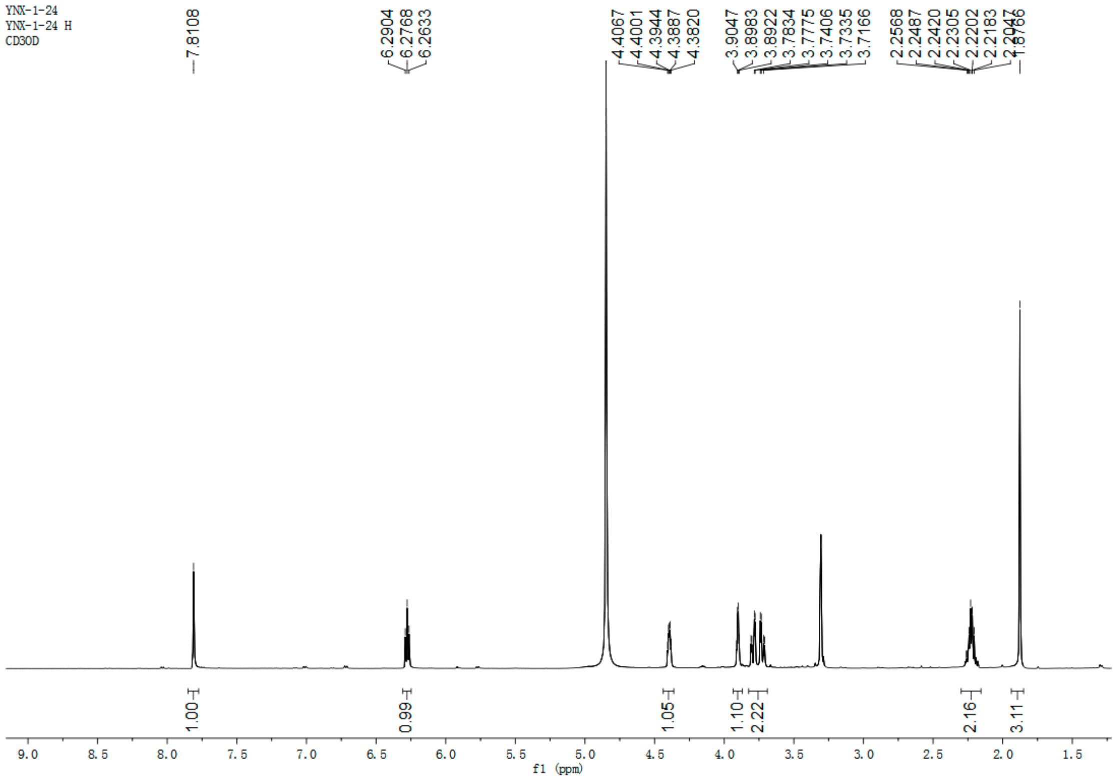

Figure S43. ^{1}H NMR spectrum (500MHz, CD_3OD) of **8**

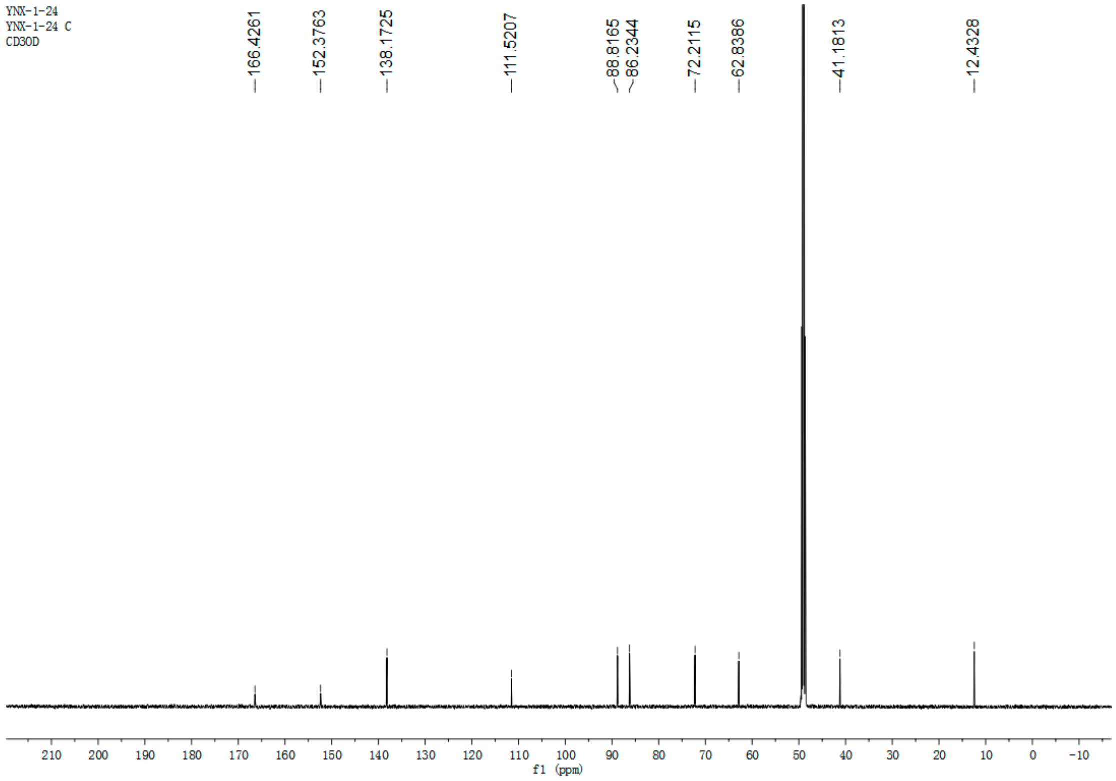

Figure S44. ^{13}C NMR spectrum (125MHz, CD_3OD) of **8**

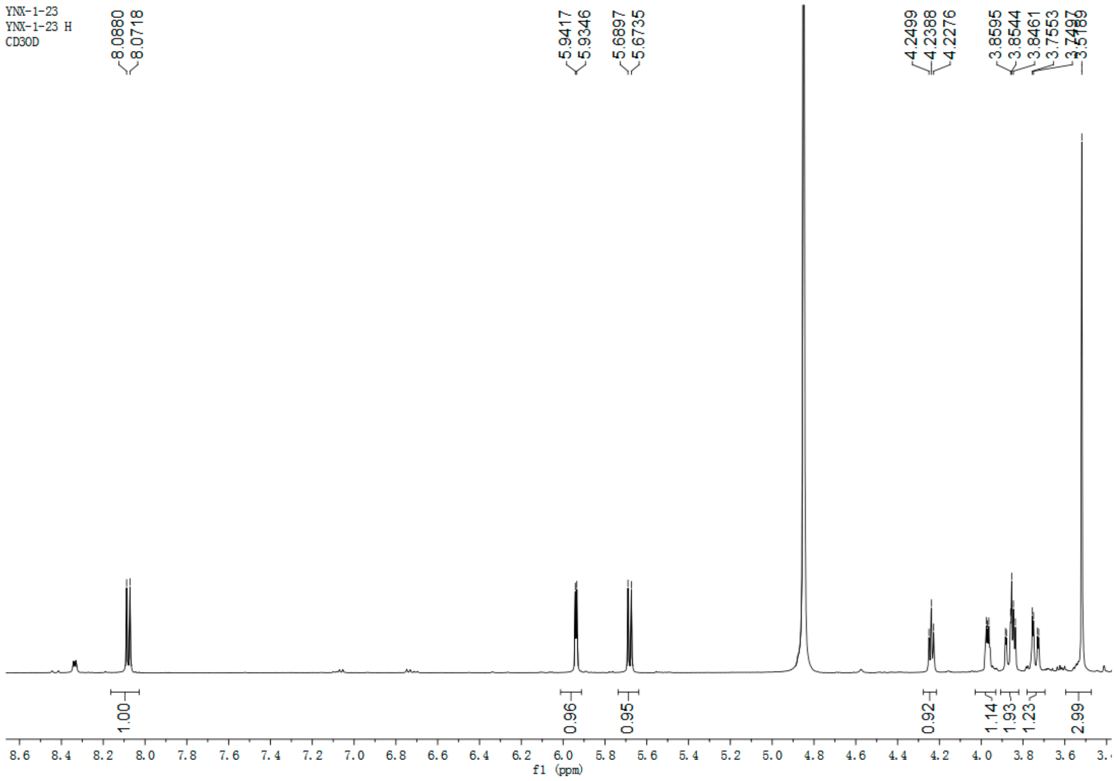

Figure S45. ^{1}H NMR spectrum (500MHz, CD_3OD) of **9**

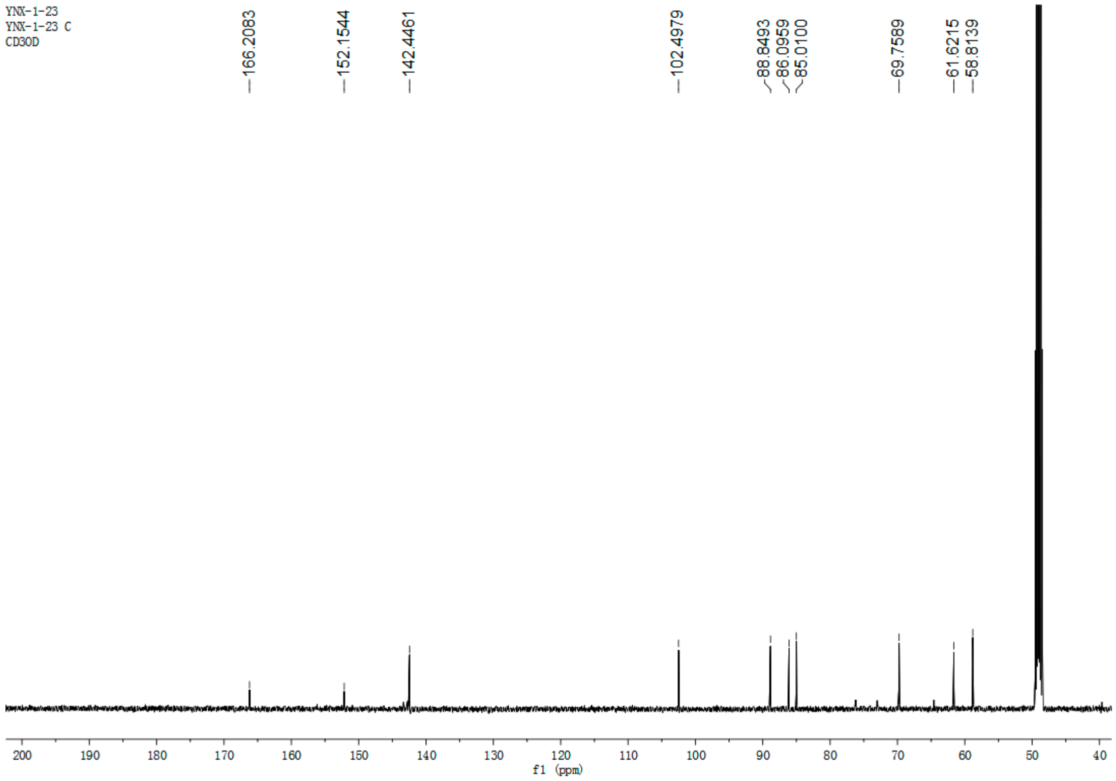

Figure S46. ^{13}C NMR spectrum (125MHz, CD_3OD) of **9**

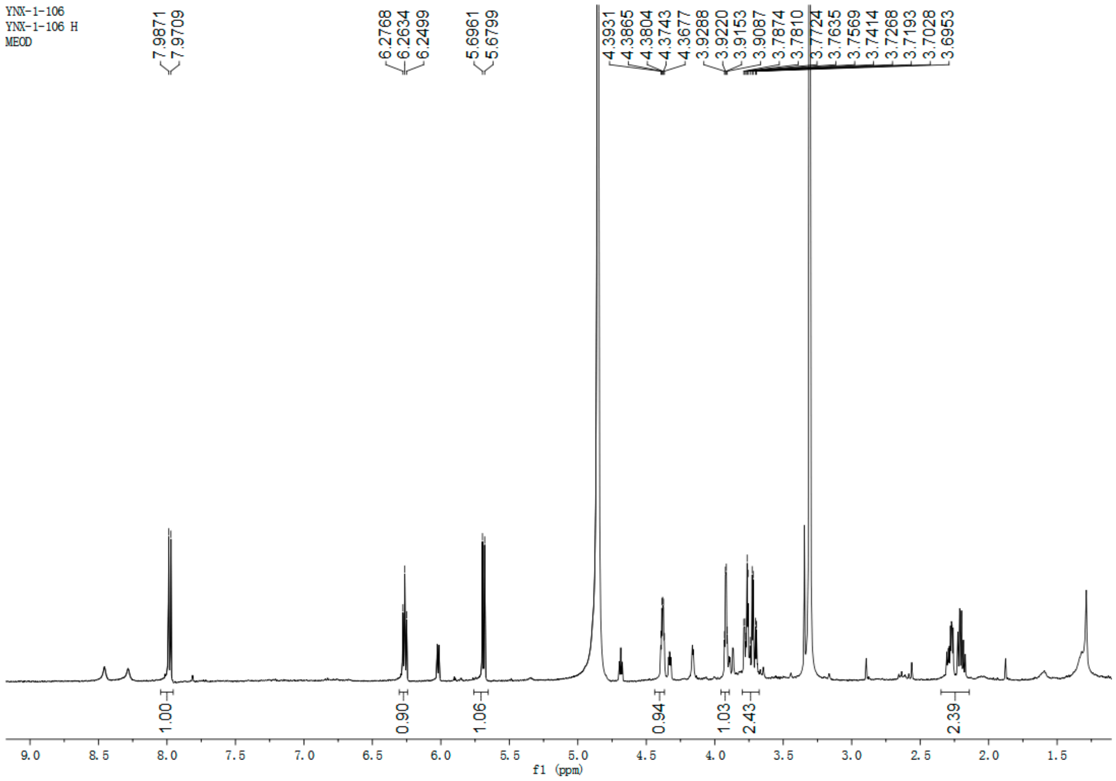

Figure S47. ^{1}H NMR spectrum (500MHz, CD_3OD) of **10**

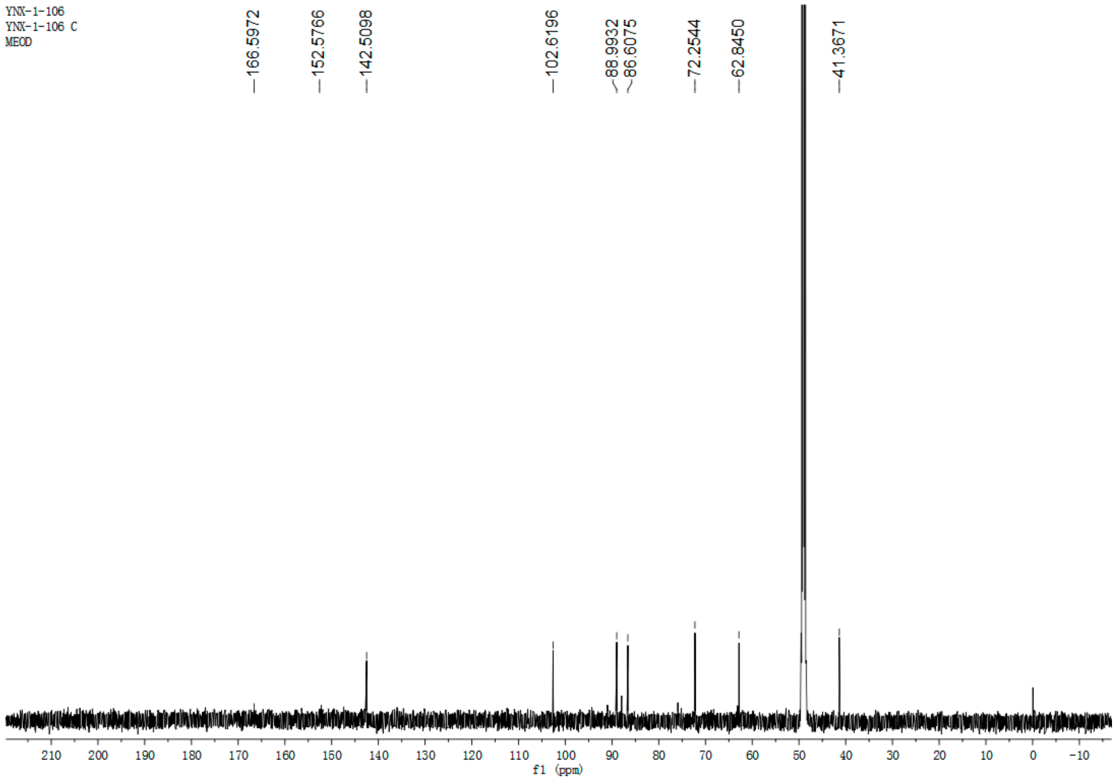

Figure S48. ^{13}C NMR spectrum (125MHz, CD_3OD) of **10**

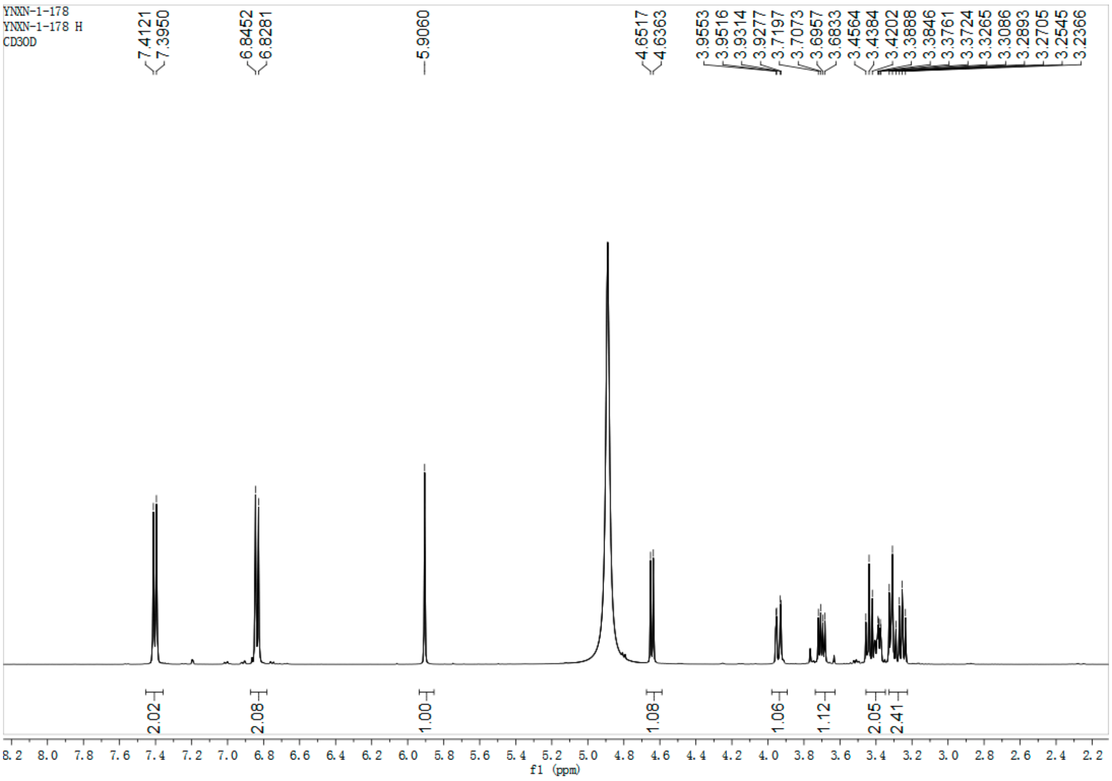

Figure S49. ^{1}H NMR spectrum (500MHz, CD_3OD) of **11**

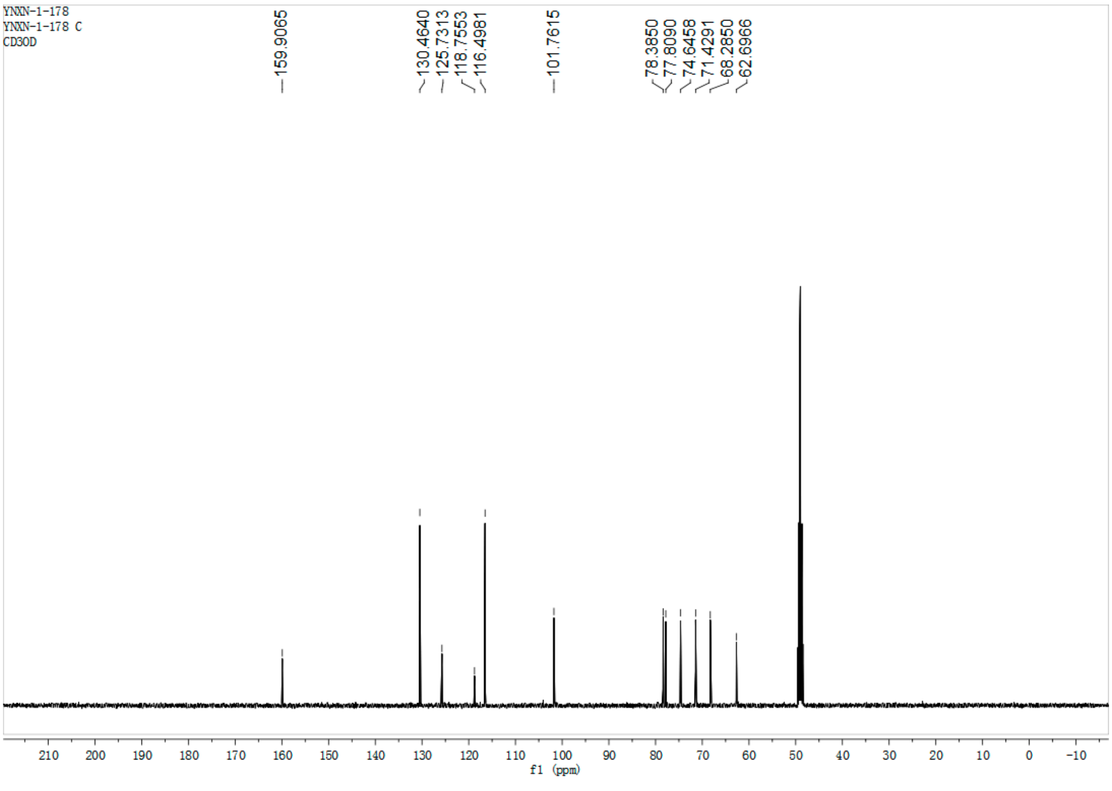

Figure S50. ^{13}C NMR spectrum (125MHz, CD_3OD) of **11**

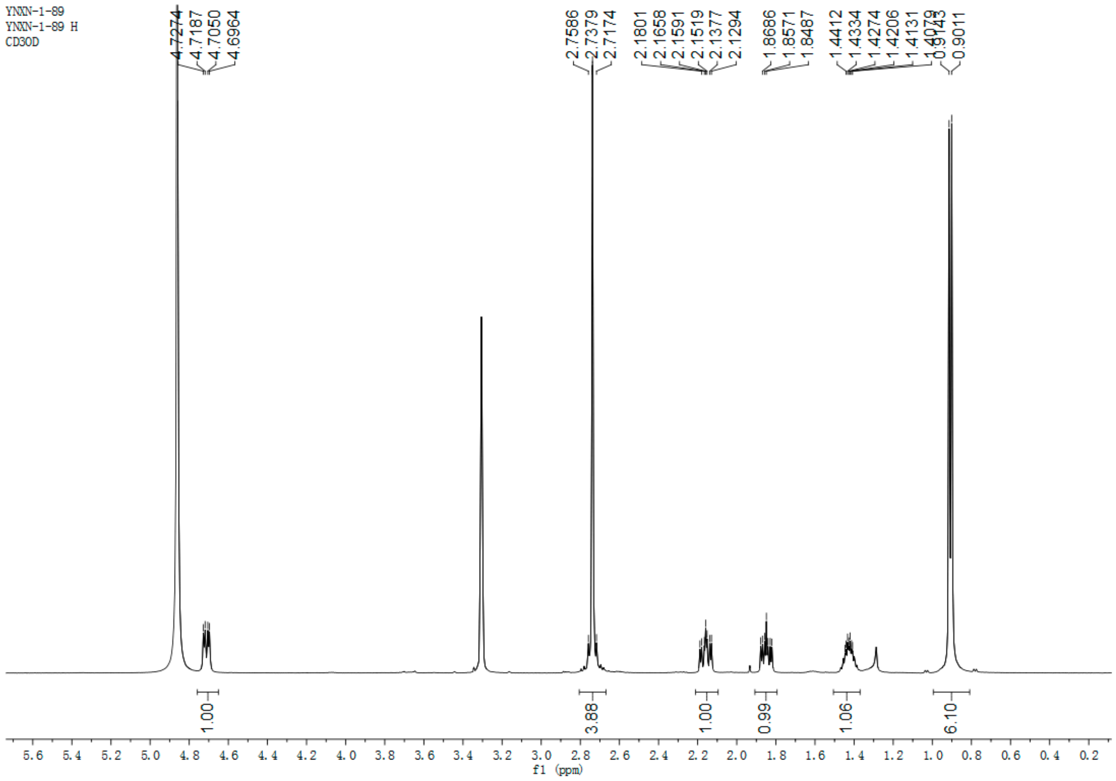

Figure S51. ^{1}H NMR spectrum (500MHz, CD_3OD) of **12**

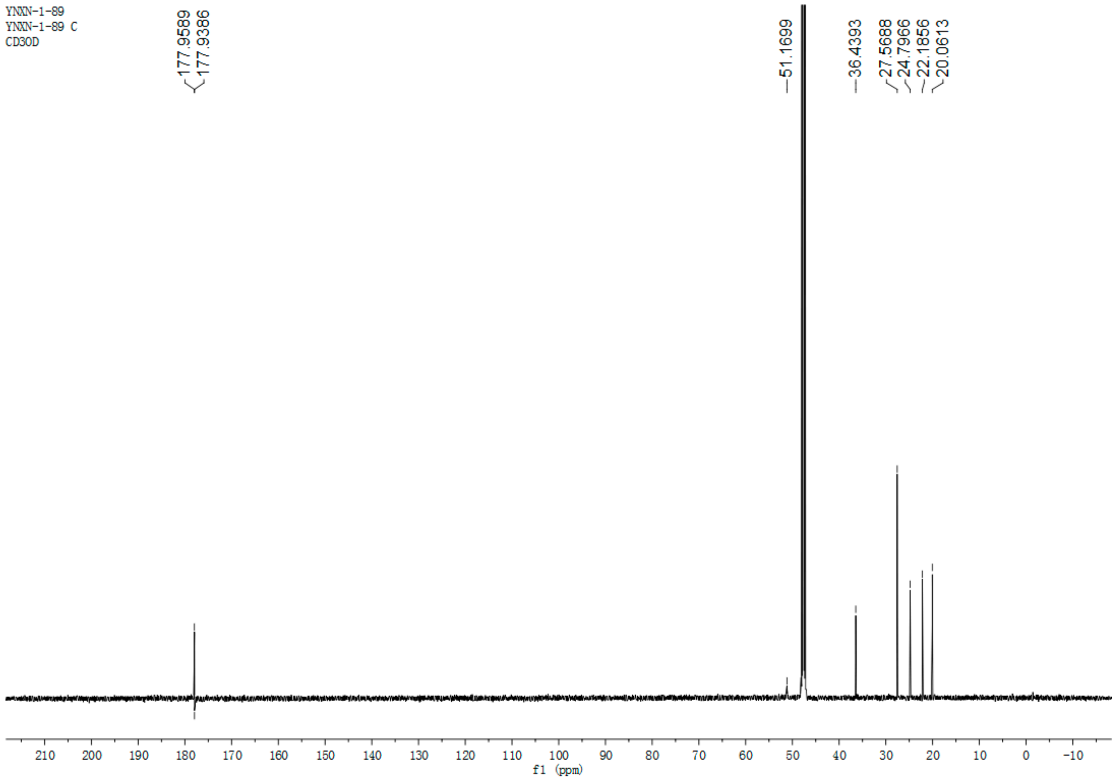

Figure S52. ^{13}C NMR spectrum (125MHz, CD_3OD) of **12**

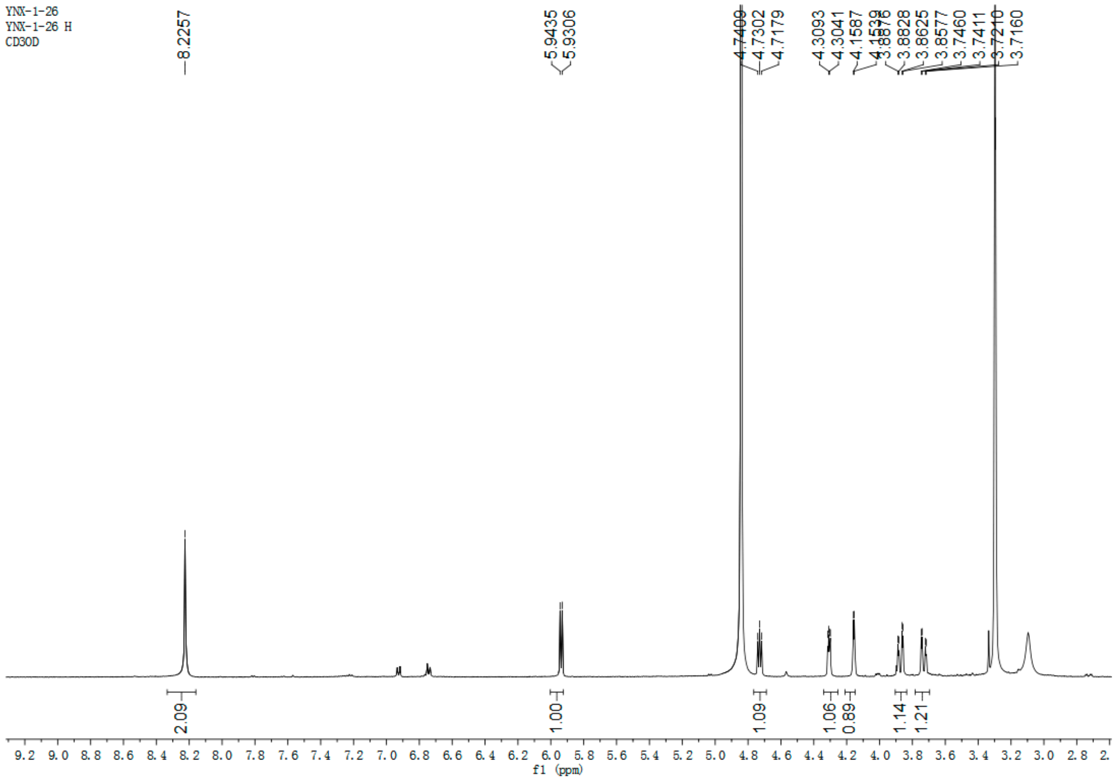

Figure S53. ^{1}H NMR spectrum (500MHz, CD_3OD) of **13**

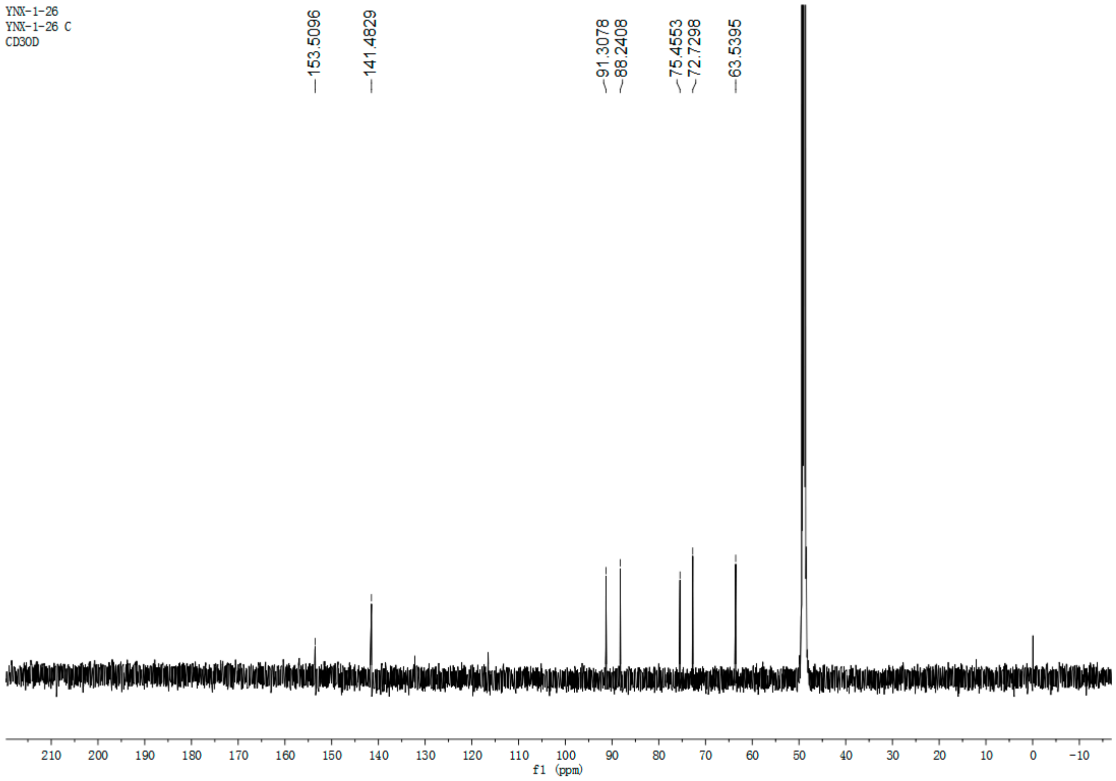

Figure S54. ^{13}C NMR spectrum (125MHz, CD_3OD) of **13**

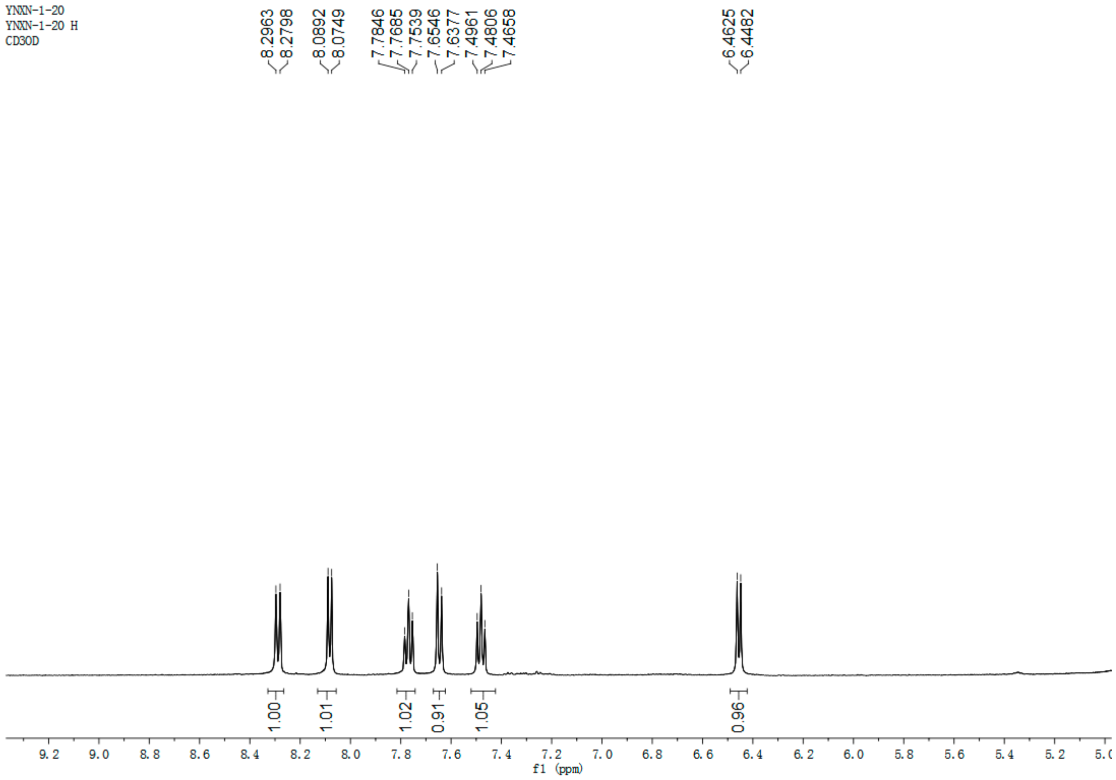

Figure S55. ^{1}H NMR spectrum (500MHz, CD_3OD) of **14**

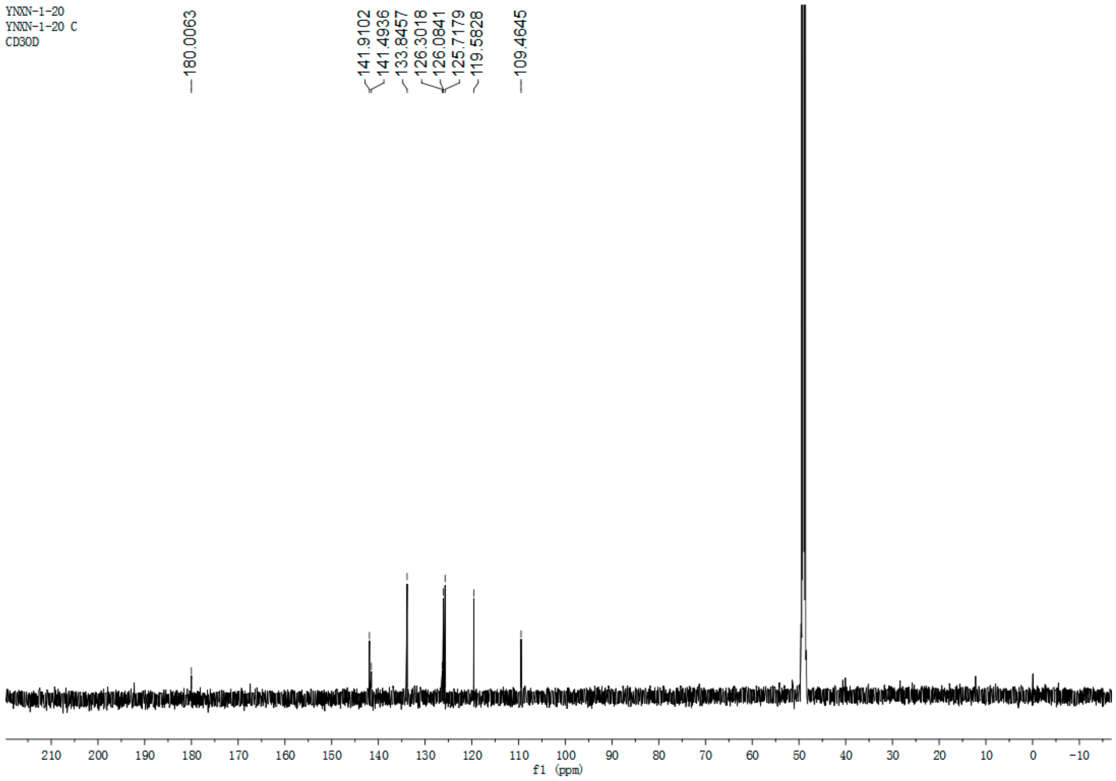

Figure S56. ^{13}C NMR spectrum (125MHz, CD_3OD) of **14**

Table S1 Coordinates of the conformers of (*R*)-**2** used after optimization and frequency.

(*R*)-**2**_01				(*R*)-**2**_02			
C	2.350796	-0.731475	-0.502704	C	2.905872	-0.572246	-0.261923
C	1.892438	0.409535	0.172718	C	2.196839	0.605776	0.02025
C	0.56946	0.419576	0.66964	C	0.812656	0.513299	0.272888
C	0.286636	-1.727761	-0.176894	C	0.944144	-1.785293	-0.035822
H	-0.367203	-2.580521	-0.317911	H	0.437547	-2.741277	-0.057631
C	1.488512	2.246169	1.130762	C	1.397684	2.523346	0.398512
H	1.612615	3.248351	1.525281	H	1.321258	3.595573	0.539278
N	-0.247565	-0.674203	0.487165	N	0.176142	-0.707826	0.25026
N	1.507262	-1.795679	-0.660096	N	2.2371	-1.763244	-0.279365
N	3.587292	-0.839109	-1.011386	N	4.222058	-0.594889	-0.520358
H	4.238786	-0.075868	-0.92297	H	4.753314	0.260939	-0.51864
H	3.862302	-1.679653	-1.494628	H	4.6814	-1.469235	-0.720817
N	2.477703	1.612829	0.484747	N	2.562526	1.928427	0.108736
N	0.298359	1.580742	1.27993	N	0.287692	1.723803	0.513002
C	-1.662298	-0.652246	0.951154	C	-1.290955	-0.743835	0.471041
H	-1.738403	0.271779	1.525919	H	-1.45369	0.018212	1.237416
C	-2.61291	-0.550553	-0.256131	C	-2.012743	-0.303975	-0.827297
H	-2.503981	-1.428759	-0.899171	H	-1.409573	0.454959	-1.329648
H	-3.63663	-0.531999	0.128096	H	-2.13985	-1.16492	-1.488883
C	-2.381575	0.744147	-1.032638	C	-3.373022	0.279017	-0.471037
C	-1.969695	-1.845324	1.852046	C	-1.789811	-2.084949	0.991864
H	-2.969134	-1.725952	2.27711	H	-2.855749	-1.989355	1.207072
H	-1.951638	-2.791193	1.30374	H	-1.681389	-2.882095	0.251576
H	-1.248265	-1.901232	2.670826	H	-1.271625	-2.37335	1.909638
C	-1.650701	0.644607	-2.348124	C	-3.408602	1.771503	-0.245441
H	-2.223193	0.019396	-3.042693	H	-3.36988	2.270656	-1.22195
H	-1.500599	1.635769	-2.776753	H	-4.325139	2.06286	0.268857
H	-0.683697	0.149388	-2.20677	H	-2.521571	2.099136	0.306578
O	-2.764538	1.807298	-0.573945	O	-4.357006	-0.432732	-0.345874

(*R*)-**2**_03				(*R*)-**2**_04			
C	2.905868	-0.572193	-0.261829	C	2.339601	-0.766958	-0.476782
C	2.196791	0.605803	0.02038	C	1.891047	0.406922	0.147571
C	0.812592	0.513259	0.272937	C	0.568179	0.44894	0.638286
C	0.944171	-1.785316	-0.035889	C	0.266175	-1.729123	-0.112649
H	0.437602	-2.741314	-0.05778	H	-0.395909	-2.580401	-0.218731
C	1.397492	2.523337	0.398498	C	1.494048	2.297046	1.000621
H	1.321006	3.595569	0.539208	H	1.619947	3.319466	1.338175
N	0.176127	-0.707894	0.250249	N	-0.255218	-0.646678	0.512319

N	2.237137	-1.763201	-0.279396	N	1.486693	-1.828498	-0.589803
N	4.22216	-0.59483	-0.519751	N	3.578036	-0.909486	-0.97295
H	4.752872	0.261342	-0.520518	H	4.221253	-0.134281	-0.954189
H	4.681021	-1.468818	-0.722868	H	3.829613	-1.755328	-1.459944
N	2.562389	1.928481	0.108774	N	2.481187	1.623804	0.395196
N	0.287554	1.723735	0.513016	N	0.300595	1.644453	1.183337
C	-1.290963	-0.743963	0.470979	C	-1.666824	-0.589255	0.979525
H	-1.453756	0.01804	1.237391	H	-1.730871	0.365587	1.502522
C	-2.012734	-0.304059	-0.827345	C	-2.621089	-0.578215	-0.230414
H	-1.409511	0.454817	-1.329721	H	-2.599558	-1.546988	-0.736662
H	-2.139958	-1.164997	-1.488924	H	-3.63406	-0.407067	0.147702
C	-3.372941	0.279076	-0.471018	C	-2.250941	0.49539	-1.246983
C	-1.78977	-2.08513	0.991714	C	-1.975164	-1.718224	1.959728
H	-2.855736	-1.989613	1.206822	H	-2.980959	-1.579263	2.363714
H	-1.681213	-2.882246	0.251409	H	-1.941059	-2.698928	1.477545
H	-1.27164	-2.373517	1.909527	H	-1.263434	-1.709659	2.788697
C	-3.408327	1.771586	-0.245516	C	-2.507079	1.932865	-0.864381
H	-4.324884	2.063101	0.268663	H	-2.353591	2.580517	-1.728465
H	-2.521306	2.099078	0.306611	H	-3.518679	2.060562	-0.467455
H	-3.369387	2.270688	-1.22204	H	-1.802642	2.218633	-0.072954
O	-4.356969	-0.432571	-0.345687	O	-1.727103	0.185706	-2.305473

(*R*)-**2**_05				(*R*)-**2**_06			
C	2.339392	-0.767397	-0.476716	C	-2.728825	-0.298229	-0.29545
C	1.891185	0.406756	0.147339	C	-1.80755	0.647704	0.177757
C	0.568364	0.449275	0.638176	C	-0.471459	0.247381	0.410856
C	0.265733	-1.728904	-0.11218	C	-1.053955	-1.890644	-0.288578
H	-0.39659	-2.580028	-0.218008	H	-0.735149	-2.910961	-0.47403
C	1.494788	2.297215	0.999869	C	-0.671982	2.289254	0.864093
H	1.62097	3.319698	1.337131	H	-0.396929	3.289612	1.178457
N	-0.255339	-0.64616	0.512554	N	-0.087586	-1.054272	0.167164
N	1.486165	-1.828737	-0.589419	N	-2.30901	-1.578349	-0.517324
N	3.577739	-0.910455	-0.972931	N	-4.013976	-0.009858	-0.549118
H	4.221188	-0.135433	-0.954505	H	-4.364648	0.920392	-0.386485
H	3.828968	-1.756486	-1.459778	H	-4.637139	-0.729944	-0.878936
N	2.481684	1.62356	0.394577	N	-1.918429	1.983287	0.480711
N	0.301127	1.645029	1.18284	N	0.259453	1.283416	0.848066
C	-1.666845	-0.588352	0.980031	C	1.303701	-1.546487	0.346487
H	-1.73073	0.366803	1.502483	H	1.22497	-2.626742	0.20333
C	-2.621334	-0.577998	-0.229731	C	2.228527	-1.038801	-0.783213
H	-2.600135	-1.547149	-0.735266	H	3.137354	-1.646779	-0.731609
H	-3.634175	-0.40635	0.14852	H	1.744751	-1.238984	-1.743134

C	-2.251192	0.494754	-1.247177	C	2.681037	0.418462	-0.699495
C	-1.975058	-1.716788	1.960891	C	1.810039	-1.287534	1.763529
H	-2.980721	-1.577477	2.365089	H	2.794982	-1.746361	1.87841
H	-1.941258	-2.697747	1.479191	H	1.893282	-0.221347	1.965528
H	-1.263104	-1.707921	2.789663	H	1.128148	-1.740357	2.488076
C	-2.50655	1.932604	-0.865497	C	2.141863	1.364862	-1.744591
H	-2.354121	2.579469	-1.730361	H	1.058127	1.266181	-1.840348
H	-3.517503	2.060938	-0.467157	H	2.406583	2.393439	-1.498388
H	-1.800859	2.21886	-0.075342	H	2.580361	1.099646	-2.715148
O	-1.727807	0.184146	-2.305628	O	3.507969	0.760853	0.129927

(*R*)-**2**_07				(*R*)-**2**_08			
C	-2.728805	-0.298243	-0.295465	C	-2.334771	-1.027573	0.059828
C	-1.807541	0.647699	0.177746	C	-1.92389	0.280918	-0.234755
C	-0.47145	0.247383	0.410862	C	-0.624449	0.69312	0.14005
C	-1.053929	-1.890651	-0.288556	C	-0.274729	-1.428726	1.027405
H	-0.735119	-2.91097	-0.473989	H	0.398153	-2.103362	1.546476
C	-0.671995	2.289251	0.864114	C	-1.613295	2.290954	-0.797625
H	-0.396956	3.289608	1.17849	H	-1.780674	3.282799	-1.202031
N	-0.087568	-1.054269	0.16718	N	0.221173	-0.190496	0.778596
N	-2.308985	-1.578364	-0.517313	N	-1.470194	-1.865371	0.704824
N	-4.013929	-0.00985	-0.549262	N	-3.546054	-1.50711	-0.259182
H	-4.364739	0.920253	-0.386086	H	-3.793285	-2.454128	-0.018792
H	-4.637223	-0.730088	-0.878501	H	-4.213659	-0.919546	-0.732461
N	-1.918435	1.983278	0.48071	N	-2.55314	1.337726	-0.846524
N	0.259448	1.283422	0.84809	N	-0.415864	1.971077	-0.210858
C	1.30372	-1.546476	0.346517	C	1.594847	0.181985	1.207722
H	1.224994	-2.626735	0.203387	H	2.022002	-0.73606	1.618653
C	2.228546	-1.038812	-0.783193	C	2.430636	0.638417	0.000252
H	3.137389	-1.646763	-0.731554	H	2.043315	1.58249	-0.38342
H	1.744784	-1.239045	-1.743111	H	3.455277	0.794595	0.358811
C	2.681009	0.418467	-0.699525	C	2.4769	-0.361324	-1.145809
C	1.81006	-1.287488	1.763553	C	1.543291	1.243266	2.306153
H	2.794996	-1.746326	1.878447	H	2.557322	1.452971	2.655995
H	1.893316	-0.221297	1.965522	H	1.102546	2.165964	1.924951
H	1.128162	-1.740281	2.488112	H	0.950359	0.887229	3.152385
C	2.141767	1.364825	-1.744627	C	2.929168	-1.771745	-0.833408
H	2.58029	1.099635	-2.715179	H	3.202589	-2.283892	-1.756629
H	1.058038	1.26607	-1.840391	H	3.767387	-1.779408	-0.130858
H	2.406416	2.393422	-1.498429	H	2.107162	-2.32615	-0.366176
O	3.507955	0.760908	0.129862	O	2.163478	-0.027502	-2.276721

(*R*)-**2**_09			
C	-2.339374	-1.026172	0.047979
C	-1.925742	0.283642	-0.23708
C	-0.625897	0.690732	0.141937
C	-0.280839	-1.438045	1.014357
H	0.390302	-2.117594	1.529287
C	-1.611221	2.297262	-0.784714
H	-1.776463	3.292335	-1.18205
N	0.217598	-0.199019	0.774852
N	-1.476803	-1.870202	0.687452
N	-3.551292	-1.500951	-0.275247
H	-3.802191	-2.447721	-0.037859
H	-4.218835	-0.907289	-0.74086
N	-2.552753	1.346082	-0.841348
N	-0.414755	1.970847	-0.19961
C	1.589995	0.168996	1.211952
H	2.015362	-0.753177	1.615445
C	2.430236	0.637465	0.012625
H	2.043657	1.584222	-0.365046
H	3.452845	0.793045	0.377717
C	2.488467	-0.350483	-1.142984
C	1.534391	1.2195	2.320651
H	2.547118	1.426036	2.676082
H	1.09474	2.145831	1.946989
H	0.938608	0.855155	3.161331
C	2.926259	-1.767178	-0.838631
H	3.209914	-2.271593	-1.763006
H	3.754115	-1.787839	-0.124052
H	2.093773	-2.320505	-0.389045
O	2.19818	-0.001565	-2.275524

Table S2 Coordinates of the conformers of (*S*)-**2** used after optimization and frequency.

(*S*)-**2**_01				(*S*)-**2**_02			
C	-2.419627	-0.720805	-0.664969	C	-2.419629	-0.720795	-0.664959
C	-2.025923	0.404292	0.074916	C	-2.025918	0.404291	0.074938
C	-0.720751	0.439088	0.611982	C	-0.720743	0.439076	0.611997
C	-0.330287	-1.654324	-0.316313	C	-0.330291	-1.654325	-0.316327
H	0.355281	-2.48093	-0.464241	H	0.355275	-2.48093	-0.464273
C	-1.713259	2.220095	1.105567	C	-1.713235	2.220088	1.105595
H	-1.881541	3.203511	1.529576	H	-1.881507	3.203504	1.529607
N	0.145944	-0.609073	0.402247	N	0.145947	-0.609087	0.402247

N	-1.531915	-1.74545	-0.840581	N	-1.531921	-1.745439	-0.840592
N	-3.633058	-0.849119	-1.221646	N	-3.633084	-0.849125	-1.22158
H	-3.863832	-1.681387	-1.741088	H	-4.319571	-0.119853	-1.113351
H	-4.319654	-0.119989	-1.11316	H	-3.863751	-1.681239	-1.741316
N	-2.660758	1.576669	0.410975	N	-2.660741	1.576673	0.411001
N	-0.504912	1.588407	1.266976	N	-0.504891	1.588391	1.266993
C	1.513769	-0.595466	0.989074	C	1.513777	-0.595489	0.98906
C	2.610435	-0.681915	-0.088682	C	2.610432	-0.681915	-0.08871
H	3.558214	-0.91275	0.402325	H	3.558218	-0.912752	0.402283
H	2.395742	-1.512139	-0.773472	H	2.395735	-1.512128	-0.773512
C	2.846735	0.570757	-0.927939	C	2.846712	0.570776	-0.927943
C	1.649882	-1.708738	2.029497	C	1.649904	-1.708781	2.02946
H	1.629926	-2.699067	1.565343	H	1.629941	-2.699102	1.565289
H	2.600643	-1.601957	2.557112	H	2.600671	-1.60201	2.557065
H	0.838706	-1.647765	2.758977	H	0.838737	-1.647821	2.758952
C	1.664145	1.248501	-1.577989	C	1.664115	1.248491	-1.578009
H	2.018547	1.974636	-2.310366	H	2.018509	1.974622	-2.310394
H	0.999892	0.522303	-2.055188	H	0.999869	0.52228	-2.055198
H	1.077059	1.769516	-0.812975	H	1.077024	1.769512	-0.813002
O	3.982497	0.994575	-1.071573	O	3.982462	0.994637	-1.07154
H	1.573226	0.370465	1.493065	H	1.573238	0.370432	1.493069

(*S*)-**2**_03				(*S*)-**2**_04			
C	-2.905872	-0.572246	-0.261923	C	-2.905868	-0.572193	-0.261829
C	-2.196838	0.605776	0.02025	C	-2.196791	0.605803	0.02038
C	-0.812655	0.513299	0.272888	C	-0.812592	0.513259	0.272937
C	-0.944144	-1.785294	-0.035822	C	-0.944172	-1.785316	-0.035889
H	-0.437548	-2.741277	-0.057631	H	-0.437602	-2.741314	-0.057779
C	-1.397683	2.523346	0.398513	C	-1.397492	2.523337	0.398498
H	-1.321257	3.595573	0.539278	H	-1.321006	3.595569	0.539208
N	-0.176142	-0.707827	0.25026	N	-0.176127	-0.707894	0.250249
N	-2.2371	-1.763244	-0.279365	N	-2.237137	-1.763201	-0.279396
N	-4.222059	-0.594888	-0.520358	N	-4.22216	-0.59483	-0.519751
H	-4.753314	0.26094	-0.518638	H	-4.752872	0.261342	-0.520518
H	-4.681401	-1.469234	-0.720816	H	-4.681021	-1.468818	-0.722867
N	-2.562525	1.928427	0.108737	N	-2.562389	1.928481	0.108774
N	-0.287692	1.723803	0.513002	N	-0.287554	1.723735	0.513016
C	1.290955	-0.743836	0.471041	C	1.290963	-0.743963	0.470979
C	2.012743	-0.303975	-0.827297	C	2.012734	-0.304059	-0.827345
H	2.139851	-1.16492	-1.488884	H	2.139958	-1.164997	-1.488924
H	1.409573	0.454959	-1.329648	H	1.409511	0.454817	-1.329721
C	3.373022	0.279017	-0.471037	C	3.372941	0.279076	-0.471018

C	1.789811	-2.08495	0.991862	C	1.78977	-2.085131	0.991714
H	1.681388	-2.882096	0.251575	H	1.681213	-2.882246	0.251409
H	2.855749	-1.989356	1.207071	H	2.855736	-1.989613	1.206822
H	1.271625	-2.373352	1.909637	H	1.27164	-2.373517	1.909526
C	3.408601	1.771503	-0.245442	C	3.408326	1.771586	-0.245515
H	4.325138	2.062861	0.268856	H	4.324884	2.063101	0.268664
H	3.369878	2.270656	-1.221951	H	3.369387	2.270688	-1.222039
H	2.52157	2.099137	0.306578	H	2.521306	2.099078	0.306611
O	4.357005	-0.432731	-0.345872	O	4.356969	-0.432571	-0.345687
H	1.45369	0.018211	1.237416	H	1.453756	0.01804	1.237391

(*S*)-**2**_05				(*S*)-**2**_06			
C	2.728825	-0.298229	-0.29545	C	2.728805	-0.298243	-0.295465
C	1.80755	0.647704	0.177757	C	1.807541	0.647699	0.177746
C	0.471459	0.247381	0.410856	C	0.47145	0.247383	0.410862
C	1.053955	-1.890644	-0.288578	C	1.053929	-1.890651	-0.288556
H	0.735149	-2.910961	-0.47403	H	0.735119	-2.91097	-0.473989
C	0.671982	2.289254	0.864093	C	0.671995	2.289251	0.864114
H	0.396929	3.289612	1.178457	H	0.396956	3.289608	1.17849
N	0.087586	-1.054272	0.167164	N	0.087568	-1.054269	0.16718
N	2.30901	-1.578349	-0.517324	N	2.308985	-1.578364	-0.517313
N	4.013976	-0.009858	-0.549118	N	4.013929	-0.00985	-0.549262
H	4.364648	0.920392	-0.386485	H	4.364739	0.920253	-0.386086
H	4.637139	-0.729944	-0.878936	H	4.637223	-0.730088	-0.878501
N	1.918429	1.983287	0.480711	N	1.918435	1.983278	0.48071
N	-0.259453	1.283416	0.848066	N	-0.259448	1.283422	0.84809
C	-1.303701	-1.546487	0.346487	C	-1.30372	-1.546476	0.346517
C	-2.228527	-1.038801	-0.783213	C	-2.228546	-1.038812	-0.783193
H	-1.744751	-1.238984	-1.743134	H	-1.744784	-1.239045	-1.743111
H	-3.137354	-1.646779	-0.731609	H	-3.137389	-1.646763	-0.731554
C	-2.681037	0.418462	-0.699495	C	-2.681009	0.418467	-0.699525
C	-1.810039	-1.287534	1.763529	C	-1.81006	-1.287488	1.763553
H	-1.893282	-0.221347	1.965528	H	-1.893316	-0.221297	1.965522
H	-2.794982	-1.746361	1.87841	H	-2.794996	-1.746326	1.878447
H	-1.128148	-1.740357	2.488076	H	-1.128162	-1.740281	2.488112
C	-2.141863	1.364862	-1.744591	C	-2.141767	1.364825	-1.744627
H	-2.406583	2.393439	-1.498388	H	-2.406416	2.393422	-1.498429
H	-1.058127	1.266181	-1.840348	H	-1.058038	1.26607	-1.840391
H	-2.580361	1.099646	-2.715148	H	-2.58029	1.099635	-2.715179
O	-3.507969	0.760853	0.129927	O	-3.507955	0.760908	0.129862
H	-1.22497	-2.626742	0.20333	H	-1.224994	-2.626735	0.203387

(*S*)-**2**_07			
C	2.33942	-1.026145	0.047897
C	1.925722	0.283673	-0.237082
C	0.625856	0.690676	0.141975
C	0.280889	-1.438179	1.014249
H	-0.390227	-2.117796	1.529132
C	1.611122	2.297349	-0.784501
H	1.776334	3.292469	-1.181743
N	-0.217609	-0.199153	0.77482
N	1.476873	-1.870252	0.687315
N	3.551362	-1.500849	-0.275335
H	3.802289	-2.447636	-0.038031
H	4.218857	-0.907156	-0.740981
N	2.552696	1.346208	-0.841232
N	0.414675	1.970829	-0.199427
C	-1.590005	0.168783	1.211966
C	-2.430245	0.637464	0.012717
H	-3.452851	0.792951	0.377854
H	-2.043665	1.584284	-0.364796
C	-2.488426	-0.35031	-1.143045
C	-1.534403	1.219134	2.320815
H	-1.094658	2.145477	1.947282
H	-2.547137	1.425719	2.676198
H	-0.938704	0.854649	3.161496
C	-2.926515	-1.766968	-0.838928
H	-3.210398	-2.271128	-1.763377
H	-2.094064	-2.320561	-0.3896
H	-3.754281	-1.787624	-0.124245
O	-2.197824	-0.001318	-2.275481
H	-2.015382	-0.753458	1.615302

Table S3. The electronic energy (EE) + thermal free energy correction and Boltzmann distribution of the conformers of (*R*)-**2** used after optimization and frequency.

conformer	EE + thermal free energy correction (Hartree)	Boltzmann distribution
(*R*)-**2**_01	-737.793619	2%
(*R*)-**2**_02	-737.792119	8%
(*R*)-**2**_03	-737.792114	8%
(*R*)-**2**_04	-737.794537	1%

(*R*)-**2**_05	-737.794544	1%
(*R*)-**2**_06	-737.790627	39%
(*R*)-**2**_07	-737.790625	39%
(*R*)-**2**_08	-737.794534	1%
(*R*)-**2**_09	-737.793411	2%

Table S4. The electronic energy (EE) + thermal free energy correction and Boltzmann distribution of the conformers of (*S*)-**2** used after optimization and frequency.

conformer	EE + thermal free energy correction (Hartree)	Boltzmann distribution
(*S*)-**2**_01	-737.790736	21%
(*S*)-**2**_02	-737.790736	21%
(*S*)-**2**_03	-737.792119	5%
(*S*)-**2**_04	-737.792114	5%
(*S*)-**2**_05	-737.790627	23%
(*S*)-**2**_06	-737.790625	23%
(*S*)-**2**_07	-737.793412	1%

Table S5. Related frequencies of the conformers of (*R*)-**2** after optimization and frequency.

(*R*)-**2**_01		(*R*)-**2**_02		(*R*)-**2**_03		(*R*)-**2**_04	
Frequency	Infrared	Frequency	Infrared	Frequency	Infrared	Frequency	Infrared
12.27	3.8480	27.73	2.9501	27.74	3.0024	20.23	3.4746
37.89	0.1662	32.94	8.1372	32.93	8.0482	32.56	7.6761
59.19	7.0835	51.46	5.6722	51.46	5.7186	72.49	7.6923
95.35	0.2767	70.50	2.5895	70.51	2.5864	92.29	2.3175
113.66	295.1708	112.49	274.3226	113.09	273.9858	133.75	2.0486
136.67	6.3475	152.73	31.0855	152.75	31.5203	146.66	301.7153
143.96	1.1485	171.01	1.4447	171.00	1.4577	165.31	2.8488
173.72	2.4377	184.11	2.7554	184.12	2.7615	172.81	6.7941
177.00	2.4208	189.23	0.9195	189.25	0.9197	182.90	0.6862
218.48	17.4592	203.39	14.3079	203.40	14.3777	220.30	23.3818
248.43	0.4120	247.67	1.1535	247.68	1.1389	243.22	0.6977
250.04	1.4376	277.93	4.2406	277.96	4.2605	249.19	3.3280
284.91	7.3091	282.45	3.0972	282.49	3.0979	283.77	5.2614

291.55	3.7742	315.84	2.3811	315.86	2.3813	292.57	3.0930
380.75	2.1832	381.02	5.7793	381.01	5.7763	382.60	4.8039
412.33	6.7174	412.29	2.2517	412.30	2.2576	426.15	4.2940
445.89	0.5709	446.67	0.2261	446.67	0.2284	451.00	1.1664
495.00	8.3403	502.25	17.9936	502.25	17.9804	494.58	8.6093
532.61	14.3602	531.29	11.7042	531.31	11.7346	532.67	16.3774
552.47	4.5670	552.75	1.8871	552.78	1.8260	550.64	3.0223
565.19	5.4326	565.00	9.5482	565.01	9.5489	566.62	10.5634
577.47	10.9678	581.35	5.2277	581.35	5.2584	580.61	7.5552
616.04	0.9964	589.71	1.1921	589.71	1.1980	610.11	2.0563
630.49	1.2617	629.24	1.3088	629.24	1.3083	630.90	1.7473
668.06	3.5077	671.25	40.9452	671.25	40.9518	668.05	4.9413
670.91	43.8968	692.07	2.3501	692.07	2.3541	671.02	44.2893
690.79	1.6846	704.48	5.3871	704.48	5.3883	690.92	2.4568
775.71	7.8297	776.21	6.9924	776.21	6.9968	777.38	7.8644
783.46	12.5438	789.46	12.7478	789.47	12.7416	783.32	13.1758
816.26	1.5536	823.57	5.6629	823.56	5.6658	801.35	4.4930
895.35	8.2108	869.42	3.7135	869.43	3.7133	892.48	4.3182
900.53	4.9890	898.82	5.1776	898.83	5.1776	899.29	4.7544
911.23	5.5382	929.92	7.3743	929.92	7.3682	919.97	4.4005
934.74	4.3500	931.47	6.8489	931.48	6.8545	937.52	4.9773
957.16	6.1848	957.88	5.4200	957.88	5.4214	957.68	6.1176
977.82	5.9343	983.15	22.4586	983.15	22.4577	985.12	4.1347
996.13	17.6607	993.95	2.3271	993.96	2.3240	996.16	27.1065
1012.94	2.3474	1016.04	16.4600	1016.05	16.4573	1014.90	1.3921
1042.42	0.5916	1041.80	1.6188	1041.80	1.6154	1045.25	1.0619
1089.35	30.5453	1094.69	19.3990	1094.70	19.3956	1094.23	26.3687
1133.94	5.4856	1125.27	6.6561	1125.27	6.6507	1133.07	1.4518
1143.96	26.3977	1137.99	27.6356	1138.00	27.6331	1139.64	35.1350
1185.14	105.7744	1187.25	172.3200	1187.26	172.3520	1188.34	131.5027
1191.45	122.6890	1205.04	105.1925	1205.04	105.1683	1198.96	45.8418
1212.53	79.8568	1229.74	10.4434	1229.73	10.4444	1213.76	86.5781
1244.31	84.0017	1252.18	82.7224	1252.18	82.5270	1244.11	73.7672
1259.12	20.3474	1254.51	63.4003	1254.51	63.5214	1263.00	46.2777
1294.19	104.0959	1296.70	64.3225	1296.71	64.3474	1298.98	92.4640
1303.04	98.1984	1302.61	89.7605	1302.60	89.7778	1303.57	100.4172
1324.43	26.6915	1328.28	179.9283	1328.28	179.9074	1318.94	66.9227
1382.56	10.5537	1373.09	15.9821	1373.10	15.9950	1382.78	14.9895
1395.50	76.4381	1397.73	39.5106	1397.73	39.5100	1400.51	58.2306
1401.35	22.7184	1400.21	29.0835	1400.21	29.0620	1402.29	56.3755
1406.52	89.5093	1413.82	100.1926	1413.82	100.2187	1407.38	71.5928
1428.39	2.8452	1430.76	8.3664	1430.75	8.3676	1427.32	2.4593
1455.25	71.4252	1458.28	137.2443	1458.28	137.1732	1456.85	71.5813

1463.22	14.5076	1467.94	6.9245	1467.93	6.9370	1469.60	29.4628
1475.17	8.5950	1475.69	54.4446	1475.68	54.3576	1478.62	12.1548
1476.41	90.1851	1479.82	20.4985	1479.81	20.4473	1481.66	165.9567
1482.12	109.6222	1482.57	103.1632	1482.58	103.3298	1483.00	17.0020
1487.30	12.5325	1487.05	24.9112	1487.05	24.9348	1489.14	15.3200
1500.39	26.9225	1502.32	34.5804	1502.32	34.5841	1499.82	20.8632
1507.33	6.5027	1518.23	5.4816	1518.24	5.4862	1505.72	7.1879
1538.32	12.2123	1538.54	18.1611	1538.53	18.1544	1537.76	12.7595
1587.08	112.0999	1585.25	106.4193	1585.24	106.4103	1589.61	112.4592
1628.94	23.3645	1629.74	24.1083	1629.72	24.1311	1629.68	23.3165
1672.86	1378.9734	1672.56	1414.1756	1672.54	1414.0670	1672.90	1356.7313
1796.83	239.3774	1791.74	278.3108	1791.77	278.3352	1789.53	250.6777
3049.18	1.3944	3041.38	3.5514	3041.38	3.5602	3037.24	15.0503
3061.99	17.7259	3063.57	16.2729	3063.55	16.2726	3060.70	15.3377
3073.19	17.0363	3079.74	7.0934	3079.72	7.0923	3069.53	19.2415
3110.86	7.1881	3096.60	4.9725	3096.56	4.9824	3110.85	1.5714
3121.94	10.9330	3105.87	6.3214	3105.86	6.3226	3120.47	12.1072
3130.35	1.4903	3138.51	23.4970	3138.49	23.5074	3129.38	1.5725
3144.17	31.7420	3144.68	15.9042	3144.67	15.8920	3143.92	36.1812
3145.60	22.5496	3153.89	11.1592	3153.88	11.1565	3145.36	18.9107
3168.36	16.0475	3165.25	13.5772	3165.22	13.5781	3162.28	15.7910
3223.96	34.4553	3224.21	33.6151	3224.20	33.6155	3225.28	33.3519
3226.64	3.7280	3248.19	1.3291	3248.17	1.3287	3228.19	4.3049
3614.24	244.0965	3615.02	249.2986	3614.97	249.2193	3612.95	240.2260
3750.94	107.5110	3751.65	107.0163	3751.60	107.0008	3749.09	106.4821

(*R*)-**2**_05		(*R*)-**2**_06		(*R*)-**2**_07	
Frequency	Infrared	Frequency	Infrared	Frequency	Infrared
20.07	3.4573	27.73	0.5692	27.72	0.5697
32.55	7.6967	69.80	1.2314	69.80	1.2369
72.46	7.6914	82.41	0.9835	82.41	0.9884
92.30	2.3160	103.46	5.1455	103.45	5.1909
133.79	1.9889	115.02	282.1273	115.22	282.0314
146.80	301.7694	158.50	15.0861	158.51	15.1736
165.33	2.8359	166.93	11.5454	166.93	11.5620
172.80	6.8023	184.98	7.1756	184.98	7.1680
182.79	0.6822	200.49	0.1030	200.49	0.1033
220.29	23.4352	212.12	17.4781	212.12	17.4720
243.31	0.6952	236.73	0.7904	236.73	0.7909
249.19	3.3248	261.88	1.6566	261.89	1.6589
283.77	5.2517	284.09	8.0209	284.10	8.0238
292.57	3.0958	296.90	2.7621	296.90	2.7611

382.61	4.8104	378.28	4.6239	378.29	4.6220
426.13	4.2943	440.21	4.6732	440.21	4.6749
450.99	1.1649	449.64	5.0183	449.64	5.0202
494.59	8.6106	503.31	7.8014	503.31	7.7985
532.68	16.3640	521.29	9.8637	521.29	9.8636
550.64	3.0251	552.80	4.1954	552.79	4.1863
566.62	10.5694	569.49	7.1416	569.49	7.1514
580.60	7.5615	580.69	6.0131	580.69	6.0126
610.11	2.0565	602.28	3.7544	602.29	3.7524
630.91	1.7474	643.18	0.3888	643.18	0.3873
668.05	4.9431	671.35	40.9466	671.35	40.9298
671.01	44.2935	674.31	9.5726	674.31	9.5950
690.92	2.4488	692.57	2.8274	692.57	2.8262
777.38	7.8636	767.89	5.5613	767.89	5.5617
783.32	13.1782	775.73	7.8274	775.73	7.8269
801.36	4.4973	815.08	11.0958	815.08	11.0949
892.52	4.3216	882.25	3.1652	882.25	3.1660
899.31	4.7542	899.22	5.2731	899.22	5.2730
919.98	4.3951	921.28	7.3351	921.28	7.3352
937.50	4.9726	931.14	4.7406	931.14	4.7411
957.66	6.1114	960.20	7.5356	960.20	7.5355
985.13	4.1422	973.05	15.7327	973.05	15.7310
996.15	27.1081	1003.54	18.0594	1003.55	18.0607
1014.88	1.3902	1012.84	10.6402	1012.84	10.6423
1045.24	1.0646	1032.59	5.3510	1032.59	5.3535
1094.22	26.3730	1085.46	17.2576	1085.46	17.2537
1133.06	1.4435	1121.32	6.3809	1121.32	6.3806
1139.64	35.1614	1152.50	25.2752	1152.50	25.2767
1188.31	131.4661	1190.77	177.2715	1190.77	177.2728
1198.96	45.7920	1200.20	122.9192	1200.20	122.9225
1213.77	86.6822	1223.74	108.2953	1223.74	108.3008
1244.13	73.7796	1252.27	39.0740	1252.27	39.0623
1263.03	46.3680	1269.04	60.8544	1269.04	60.8491
1298.98	92.4291	1302.02	108.8424	1302.02	108.8425
1303.59	100.4051	1309.14	26.0942	1309.14	26.1031
1318.94	66.8698	1324.90	8.5380	1324.90	8.5352
1382.76	14.9661	1383.48	99.3165	1383.48	99.3229
1400.53	58.6524	1389.57	31.2861	1389.57	31.2776
1402.30	55.9027	1402.40	46.4389	1402.40	46.4473
1407.39	71.6317	1407.70	45.6574	1407.70	45.6514
1427.33	2.4573	1428.07	18.9634	1428.07	18.9628
1456.85	71.4910	1446.77	65.7700	1446.77	65.7714
1469.63	29.5579	1462.89	6.7097	1462.89	6.7100

1478.60	12.0817	1469.26	73.6561	1469.26	73.6420
1481.64	165.5098	1477.44	8.8669	1477.44	8.8651
1482.99	17.5466	1479.74	13.9945	1479.74	13.9942
1489.12	15.2723	1490.41	81.0952	1490.41	81.0951
1499.83	20.8384	1494.08	109.5171	1494.08	109.5342
1505.73	7.1957	1511.67	5.5203	1511.68	5.5208
1537.78	12.7125	1535.75	5.8746	1535.75	5.8759
1589.63	112.5124	1593.34	100.5029	1593.34	100.4952
1629.69	23.2832	1628.38	15.0650	1628.38	15.0642
1672.93	1356.8489	1672.15	1456.0541	1672.15	1456.0070
1789.50	250.6848	1792.46	243.4768	1792.46	243.4782
3037.14	15.0981	3045.80	2.8359	3045.79	2.8358
3060.68	15.3568	3067.12	20.2475	3067.12	20.2527
3069.51	19.2239	3070.00	16.6609	3070.00	16.6559
3110.83	1.5873	3095.48	28.6403	3095.48	28.6405
3120.46	12.1328	3121.90	16.8137	3121.91	16.8069
3129.37	1.5676	3123.05	15.1981	3123.05	15.2052
3143.90	36.1923	3139.56	32.2591	3139.56	32.2595
3145.35	18.8823	3171.60	18.4051	3171.60	18.4042
3162.29	15.7859	3187.35	4.0638	3187.35	4.0633
3225.27	33.3620	3212.36	6.2239	3212.36	6.2237
3228.20	4.3078	3224.33	32.5377	3224.33	32.5377
3612.94	240.2326	3614.69	245.6014	3614.69	245.5838
3749.07	106.4832	3751.30	106.8738	3751.29	106.8684

(*R*)-**2**_08		(*R*)-**2**_09	
Frequency	Infrared	Frequency	Infrared
11.13	7.2003	30.81	7.3770
37.82	1.1120	40.41	0.4008
53.39	1.1480	56.77	1.8979
106.47	7.0638	107.61	11.5754
119.57	226.5986	118.81	242.9903
124.41	43.9034	126.23	25.5670
159.60	16.4097	161.75	14.8790
174.79	3.6539	175.75	3.1713
178.75	5.4957	179.31	5.2348
213.78	19.1576	214.01	18.9785
239.74	0.2179	239.67	0.2260
264.88	2.8947	265.39	3.0000
283.14	6.6904	283.10	6.5250
305.20	2.2982	305.42	2.2249
397.77	7.8236	398.05	8.1031

410.15	5.2837	409.44	5.1759
463.06	0.3048	463.99	0.2873
506.18	0.0769	505.96	0.0914
526.09	11.0180	525.90	10.8861
553.66	5.8825	553.19	5.7525
568.76	9.6533	568.07	9.7134
576.65	6.7444	576.79	6.8152
595.77	7.3314	595.67	7.3649
620.75	9.4608	620.93	9.7921
646.95	0.5679	646.90	0.5590
671.18	41.2037	671.15	41.1353
691.22	3.3802	691.47	3.4028
774.95	7.8506	775.03	7.9137
786.33	11.0792	786.00	10.9988
795.37	4.3609	795.32	4.5414
885.43	5.4258	884.65	5.5088
899.94	5.0540	899.95	5.0545
930.73	1.7613	931.49	1.5800
932.48	7.3716	933.09	7.7242
959.86	7.0964	959.93	7.0973
990.05	8.1727	990.31	7.9637
995.76	6.6915	995.64	6.4340
1006.81	20.3016	1006.61	20.4663
1030.88	6.0524	1030.77	6.1741
1102.31	26.8015	1102.28	26.9891
1123.73	7.6411	1123.64	8.0183
1147.35	38.2686	1147.97	37.6765
1190.94	171.2344	1191.04	171.9819
1203.37	49.1002	1203.88	49.2959
1222.98	112.1041	1223.15	110.9419
1253.76	24.6826	1253.76	24.7266
1268.38	89.1688	1268.26	88.6154
1304.27	101.1473	1304.26	101.4922
1314.28	19.0695	1314.67	18.8603
1325.58	5.4296	1325.70	5.3365
1377.29	46.5270	1377.85	48.3802
1389.29	104.2504	1389.50	102.3726
1396.09	62.6261	1395.80	61.4586
1407.50	20.6851	1407.94	22.0681
1419.68	14.0886	1419.67	14.0222
1446.19	65.0020	1446.29	64.3698
1466.63	82.7610	1466.30	78.9316
1470.39	8.1784	1470.34	11.0517

1471.43	19.9284	1471.61	21.7222
1484.55	3.3001	1484.42	3.0933
1491.83	178.6020	1491.97	176.2138
1495.30	20.2869	1495.39	22.3284
1511.25	1.3700	1511.26	1.5204
1536.26	4.4253	1536.27	4.4757
1590.95	95.8484	1591.00	95.3137
1628.06	17.1976	1628.08	16.8765
1671.94	1417.1726	1671.94	1418.7246
1793.34	256.1403	1793.30	257.1300
3052.75	3.4717	3052.53	3.6977
3059.03	8.6701	3058.00	8.8105
3065.21	28.6808	3065.05	28.2810
3097.77	20.3480	3097.22	20.3473
3119.60	10.2566	3119.32	9.9359
3140.03	29.1447	3139.99	29.1816
3154.81	0.1580	3154.86	0.1875
3160.98	14.8651	3160.86	14.8002
3165.64	13.8219	3165.62	13.8907
3211.68	7.0355	3211.30	7.0010
3224.22	33.9401	3224.09	33.8945
3614.18	244.1215	3614.61	244.3096
3750.74	107.2554	3751.17	107.3376

Table S6. Related frequencies of the conformers of (*S*)-**2** after optimization and frequency.

(*S*)-**2**_01		(*S*)-**2**_02		(*S*)-**2**_03		(*S*)-**2**_04	
Frequency	Infrared	Frequency	Infrared	Frequency	Infrared	Frequency	Infrared
33.14	5.2011	33.14	5.2013	27.73	2.9501	27.74	3.0024
39.63	0.9871	39.63	0.9873	32.94	8.1371	32.93	8.0481
59.70	2.5123	59.71	2.5124	51.46	5.6723	51.46	5.7186
107.27	0.4386	107.28	0.4453	70.50	2.5895	70.51	2.5864
125.51	299.4658	125.49	299.4657	112.49	274.3231	113.09	273.9859
154.93	6.3896	154.93	6.3885	152.73	31.0851	152.75	31.5202
172.72	2.2059	172.72	2.2041	171.01	1.4448	171.00	1.4577
183.60	1.6620	183.60	1.6592	184.11	2.7554	184.12	2.7615
216.80	11.6428	216.80	11.6445	189.23	0.9195	189.25	0.9197
230.04	3.5035	230.04	3.5016	203.39	14.3078	203.40	14.3777
249.70	0.5484	249.70	0.5480	247.67	1.1535	247.68	1.1390
252.64	1.2791	252.64	1.2786	277.93	4.2406	277.96	4.2605
281.08	5.9998	281.08	6.0063	282.45	3.0972	282.49	3.0979
288.04	3.6357	288.03	3.6300	315.84	2.3811	315.86	2.3813

376.31	8.8928	376.31	8.8932	381.02	5.7793	381.01	5.7763
403.84	8.4834	403.83	8.4836	412.29	2.2517	412.30	2.2576
463.56	0.6957	463.56	0.6950	446.67	0.2261	446.67	0.2284
499.87	6.0847	499.87	6.0862	502.25	17.9935	502.25	17.9804
538.03	9.4495	538.03	9.4568	531.29	11.7042	531.31	11.7346
552.21	6.4172	552.21	6.3950	552.75	1.8871	552.78	1.8260
565.16	7.9081	565.16	7.9204	565.00	9.5482	565.01	9.5489
571.35	6.9941	571.35	6.9947	581.35	5.2277	581.35	5.2584
605.27	9.3258	605.27	9.3238	589.71	1.1921	589.71	1.1980
630.45	1.6198	630.45	1.6215	629.24	1.3088	629.24	1.3083
670.99	21.2405	670.98	21.2500	671.25	40.9452	671.25	40.9518
671.59	26.5440	671.59	26.5326	692.07	2.3501	692.07	2.3541
692.44	2.6187	692.44	2.6189	704.48	5.3871	704.48	5.3883
777.75	7.8339	777.75	7.8158	776.21	6.9924	776.21	6.9968
778.56	14.2301	778.56	14.2490	789.46	12.7478	789.47	12.7416
797.83	5.0495	797.83	5.0489	823.57	5.6629	823.56	5.6658
872.42	0.8216	872.43	0.8217	869.42	3.7135	869.43	3.7133
900.30	5.1026	900.30	5.1025	898.82	5.1776	898.83	5.1776
926.09	8.3636	926.09	8.3633	929.92	7.3741	929.92	7.3682
946.05	4.1843	946.05	4.1847	931.47	6.8491	931.48	6.8545
957.24	6.0354	957.24	6.0356	957.88	5.4200	957.88	5.4214
987.96	10.9732	987.95	10.9759	983.15	22.4586	983.15	22.4577
998.28	13.0196	998.28	13.0162	993.95	2.3271	993.96	2.3240
1017.69	2.1816	1017.69	2.1818	1016.04	16.4599	1016.05	16.4573
1038.80	2.2273	1038.80	2.2269	1041.80	1.6188	1041.80	1.6154
1086.83	17.6509	1086.83	17.6503	1094.69	19.3990	1094.70	19.3956
1130.32	11.8343	1130.33	11.8332	1125.27	6.6561	1125.27	6.6507
1153.72	20.7569	1153.72	20.7588	1137.99	27.6355	1138.00	27.6331
1187.39	155.8859	1187.39	155.8882	1187.25	172.3198	1187.26	172.3520
1202.27	60.2467	1202.27	60.2432	1205.04	105.1928	1205.04	105.1683
1214.63	62.5845	1214.63	62.5860	1229.74	10.4433	1229.73	10.4444
1251.18	127.8242	1251.18	127.8248	1252.18	82.7226	1252.18	82.5271
1269.86	43.4899	1269.86	43.4907	1254.51	63.4003	1254.51	63.5214
1298.61	83.1197	1298.61	83.1197	1296.70	64.3225	1296.71	64.3474
1303.61	100.9617	1303.61	100.9607	1302.61	89.7604	1302.60	89.7778
1323.38	28.8417	1323.38	28.8418	1328.28	179.9283	1328.28	179.9074
1388.32	9.5548	1388.32	9.5547	1373.09	15.9821	1373.10	15.9951
1399.16	23.2682	1399.16	23.2681	1397.73	39.5105	1397.73	39.5100
1404.26	27.0202	1404.26	27.0262	1400.21	29.0838	1400.21	29.0620
1407.11	101.0966	1407.11	101.0897	1413.82	100.1925	1413.82	100.2187
1429.69	2.9948	1429.69	2.9942	1430.76	8.3664	1430.75	8.3676
1454.91	80.6508	1454.91	80.6512	1458.28	137.2449	1458.28	137.1733
1466.31	5.7220	1466.31	5.7255	1467.94	6.9245	1467.93	6.9370

1475.35	106.1721	1475.35	106.1976	1475.69	54.4441	1475.68	54.3576
1478.70	100.0133	1478.70	100.0120	1479.82	20.4982	1479.81	20.4472
1481.94	73.8998	1481.94	73.8648	1482.57	103.1639	1482.58	103.3299
1485.98	8.3906	1485.98	8.3917	1487.05	24.9112	1487.05	24.9349
1502.84	15.0605	1502.84	15.0609	1502.32	34.5803	1502.32	34.5841
1504.62	8.1629	1504.62	8.1632	1518.23	5.4817	1518.24	5.4862
1537.60	11.3539	1537.59	11.3511	1538.54	18.1609	1538.53	18.1544
1588.94	113.8620	1588.94	113.8624	1585.25	106.4194	1585.24	106.4103
1628.91	22.7903	1628.91	22.7946	1629.74	24.1084	1629.72	24.1311
1673.01	1398.3979	1673.01	1398.4119	1672.56	1414.1753	1672.54	1414.0670
1788.42	294.8068	1788.42	294.8062	1791.74	278.3108	1791.77	278.3353
3050.05	3.3147	3050.05	3.3154	3041.38	3.5514	3041.38	3.5602
3054.27	8.8265	3054.28	8.8261	3063.57	16.2729	3063.55	16.2726
3059.80	19.9190	3059.80	19.9194	3079.74	7.0934	3079.72	7.0923
3118.96	3.8470	3118.96	3.8469	3096.60	4.9725	3096.56	4.9824
3120.90	9.6949	3120.90	9.6949	3105.87	6.3214	3105.86	6.3226
3128.52	9.3019	3128.52	9.3016	3138.51	23.4971	3138.49	23.5074
3140.09	28.7163	3140.09	28.7166	3144.68	15.9041	3144.67	15.8919
3146.13	29.2446	3146.13	29.2450	3153.89	11.1591	3153.88	11.1565
3165.03	15.5799	3165.03	15.5802	3165.25	13.5772	3165.22	13.5781
3223.50	4.8618	3223.50	4.8618	3224.21	33.6151	3224.20	33.6155
3225.07	33.4891	3225.07	33.4891	3248.19	1.3291	3248.17	1.3287
3614.48	248.3500	3614.48	248.3605	3615.02	249.2987	3614.97	249.2193
3750.97	108.2494	3750.98	108.2527	3751.65	107.0163	3751.60	107.0008

(*S*)-**2**_05		(*S*)-**2**_06		(*S*)-**2**_07	
Frequency	Infrared	Frequency	Infrared	Frequency	Infrared
27.73	0.5692	27.72	0.5697	30.76	7.3861
69.80	1.2314	69.80	1.2369	40.39	0.3988
82.41	0.9835	82.41	0.9884	56.74	1.8880
103.46	5.1455	103.45	5.1909	107.61	11.3271
115.02	282.1273	115.22	282.0313	118.91	242.7311
158.50	15.0861	158.51	15.1736	126.26	26.0198
166.93	11.5454	166.93	11.5620	161.78	14.9042
184.98	7.1756	184.98	7.1680	175.76	3.1789
200.49	0.1030	200.49	0.1033	179.32	5.2322
212.12	17.4781	212.12	17.4720	214.02	19.0034
236.73	0.7904	236.73	0.7909	239.70	0.2260
261.88	1.6566	261.89	1.6589	265.40	3.0028
284.09	8.0209	284.10	8.0238	283.11	6.5230
296.90	2.7621	296.90	2.7611	305.42	2.2258
378.28	4.6239	378.29	4.6220	398.05	8.1017

440.21	4.6732	440.21	4.6749	409.46	5.1765
449.64	5.0183	449.64	5.0202	463.98	0.2871
503.31	7.8014	503.31	7.7985	505.97	0.0915
521.29	9.8637	521.29	9.8636	525.89	10.8939
552.80	4.1954	552.79	4.1863	553.21	5.7535
569.49	7.1416	569.49	7.1514	568.09	9.7114
580.69	6.0131	580.69	6.0126	576.79	6.8133
602.28	3.7544	602.29	3.7524	595.67	7.3651
643.18	0.3888	643.18	0.3873	620.93	9.7865
671.35	40.9466	671.35	40.9298	646.90	0.5590
674.31	9.5726	674.31	9.5950	671.15	41.1306
692.57	2.8274	692.57	2.8262	691.47	3.4052
767.89	5.5613	767.89	5.5617	775.03	7.9138
775.73	7.8274	775.73	7.8269	786.00	10.9988
815.08	11.0958	815.08	11.0949	795.31	4.5433
882.25	3.1652	882.25	3.1660	884.68	5.5090
899.22	5.2731	899.22	5.2730	899.97	5.0552
921.28	7.3351	921.28	7.3352	931.49	1.5730
931.14	4.7406	931.14	4.7411	933.10	7.7281
960.20	7.5356	960.20	7.5355	959.93	7.0966
973.05	15.7327	973.05	15.7310	990.31	7.9677
1003.54	18.0594	1003.55	18.0607	995.64	6.4413
1012.84	10.6402	1012.84	10.6423	1006.61	20.4664
1032.59	5.3510	1032.59	5.3535	1030.78	6.1741
1085.46	17.2576	1085.46	17.2537	1102.27	26.9671
1121.32	6.3809	1121.32	6.3806	1123.64	8.0206
1152.50	25.2752	1152.50	25.2767	1147.97	37.6963
1190.77	177.2715	1190.77	177.2728	1191.04	171.9697
1200.20	122.9192	1200.20	122.9225	1203.87	49.3005
1223.74	108.2953	1223.74	108.3008	1223.15	110.9681
1252.27	39.0740	1252.27	39.0623	1253.76	24.7337
1269.04	60.8544	1269.04	60.8491	1268.26	88.6086
1302.02	108.8424	1302.02	108.8425	1304.25	101.4986
1309.14	26.0942	1309.14	26.1031	1314.67	18.8592
1324.90	8.5380	1324.90	8.5352	1325.69	5.3405
1383.48	99.3165	1383.48	99.3229	1377.85	48.3467
1389.57	31.2861	1389.57	31.2776	1389.50	102.4091
1402.40	46.4389	1402.40	46.4473	1395.80	61.4733
1407.70	45.6574	1407.70	45.6514	1407.94	22.0654
1428.07	18.9634	1428.07	18.9628	1419.68	14.0212
1446.77	65.7700	1446.77	65.7714	1446.29	64.3418
1462.89	6.7097	1462.89	6.7100	1466.30	79.1332
1469.26	73.6561	1469.26	73.6420	1470.34	10.9466

1477.44	8.8669	1477.44	8.8651	1471.61	21.6810
1479.74	13.9945	1479.74	13.9942	1484.43	3.0902
1490.41	81.0952	1490.41	81.0951	1491.96	176.3551
1494.08	109.5171	1494.08	109.5342	1495.39	22.1224
1511.67	5.5203	1511.68	5.5208	1511.26	1.5182
1535.75	5.8746	1535.75	5.8759	1536.26	4.4748
1593.34	100.5029	1593.34	100.4952	1590.99	95.2940
1628.38	15.0650	1628.38	15.0642	1628.08	16.8324
1672.15	1456.0540	1672.15	1456.0070	1671.93	1418.8363
1792.46	243.4768	1792.46	243.4782	1793.30	257.1300
3045.80	2.8359	3045.79	2.8358	3052.51	3.6942
3067.12	20.2475	3067.12	20.2527	3058.00	8.8143
3070.00	16.6609	3070.00	16.6559	3065.03	28.2853
3095.48	28.6403	3095.48	28.6405	3097.18	20.3434
3121.90	16.8137	3121.91	16.8069	3119.31	9.9414
3123.05	15.1981	3123.05	15.2052	3139.98	29.1822
3139.56	32.2591	3139.56	32.2595	3154.85	0.1903
3171.60	18.4051	3171.60	18.4042	3160.84	14.7956
3187.35	4.0638	3187.35	4.0633	3165.59	13.8911
3212.36	6.2239	3212.36	6.2237	3211.27	7.0002
3224.33	32.5377	3224.33	32.5377	3224.05	33.8958
3614.69	245.6015	3614.69	245.5838	3614.58	244.3212
3751.30	106.8739	3751.29	106.8684	3751.14	107.3409
